# Molecular Phylogenetic Analysis of the *AIG* Family in Vertebrates

**DOI:** 10.3390/genes12081190

**Published:** 2021-07-30

**Authors:** Yuqi Huang, Minghao Sun, Lenan Zhuang, Jin He

**Affiliations:** 1 Department of Animal Science, College of Animal Sciences, Zhejiang University, Hangzhou 310058, China; 3180100636@zju.edu.cn; 2 Department of Veterinary Medicine, College of Animal Sciences, Zhejiang University, Hangzhou 310058, China; mhsun29@zju.edu.cn

**Keywords:** AIG1, ADTRP, phylogenetics, molecular evolution

## Abstract

*Androgen-inducible genes* (*AIGs*), which can be regulated by androgen level, constitute a group of genes characterized by the presence of the AIG/FAR-17a domain in its protein sequence. Previous studies on AIGs demonstrated that one member of the gene family, *AIG1,* is involved in many biological processes in cancer cell lines and that *ADTRP* is associated with cardiovascular diseases. It has been shown that the numbers of *AIG* paralogs in humans, mice, and zebrafish are 2, 2, and 3, respectively, indicating possible gene duplication events during vertebrate evolution. Therefore, classifying subgroups of *AIGs* and identifying the homologs of each *AIG* member are important to characterize this novel gene family further. In this study, vertebrate *AIGs* were phylogenetically grouped into three major clades, *ADTRP*, *AIG1*, and *AIG-L*, with *AIG-L* also evident in an outgroup consisting of invertebrsate species. In this case, *AIG-L,* as the ancestral *AIG,* gave rise to *ADTRP* and *AIG1* after two rounds of whole-genome duplications during vertebrate evolution. Then, the *AIG* family, which was exposed to purifying forces during evolution, lost or gained some of its members in some species. For example, in eutherians, Neognathae, and Percomorphaceae, *AIG-L* was lost; in contrast, Salmonidae and Cyprinidae acquired additional *AIG* copies. In conclusion, this study provides a comprehensive molecular phylogenetic analysis of vertebrate *AIGs*, which can be employed for future functional characterization of *AIGs*.

## 1. Introduction

*Androgen-inducible gene* (*AIG*) or *FAR-17a* is a newly identified gene first characterized in the Syrian golden hamster as an androgen-responsive gene [1]. Later, human *androgen-inducible gene 1* (*hAIG1*), which was found to be a homolog of hamster *FAR-17a,* was isolated from cultured human dermal papilla cells [2]. *hAIG1* encodes a predicted integral membrane protein with expression observed across many tissues. Additionally, the expression level of *hAIG1* was upregulated by dihydrotestosterone (DHT, androgen). Then, another androgen-regulated gene, *C6ORF105* (*ADTRP*), which encodes a transmembrane protein and shows sequence similarity with *hAIG1*, was identified with the characteristic FAR-17a/AIG domain in the predicted amino acid sequence. Thus, *hAIG1* and *hADTRP* are likely to be paralogs and form an *AIG* family [3].

Functionally, an initial study first revealed that the AIG1 protein can interact with the p53-induced RING-H2 protein (Pirh2) and activate the NFAT signaling pathway. In human hepatocellular carcinoma (HCC), *hAIG1* expression was correlated with HCC patient survival rates and thus can be used as a novel biomarker for the progression of HCC [4]. Other studies also indicated that *AIG1* is involved in cancer-related processes; e.g., *AIG1* can form complexes with either nuclear factor 1/B in salivary adenoid cystic carcinoma or Golgi SNAR complex member 1 in T cell lymphoma [5,6]. Zhu et al. reported that when KBM7 mucells were treated with chlorpyrifos, *AIG1* was required for resistance to these environmental toxicants [7]. Another study by Nickel et al. further demonstrated that *AIG1*, as an endoplasmic reticulum (ER) integral membrane protein with a sex-specific expression pattern, participates in the regulation of ER Ca^2+^ levels and cell death [8].

In contrast to that of its paralog, the validation of *ADTRP* was provoked by its potential roles in cardiovascular diseases. Tissue factor pathway inhibitor (TFPI) is a vital anticoagulation factor that inhibits factor Xa and factor VIIa to prevent prothrombotic diseases, including coronary artery disease (CAD) [9,10]. Therefore, finding the genes/proteins that can elevate the expression or prevent TFPI downregulation might benefit human health. In a global meta-analysis (GAMMA) against several human microarray datasets, Lupu et al. found that a novel gene, *C6ORF105*, was positively correlated with TFPI [3]. A subsequent experimental study confirmed that *C6ORF105* colocalized with TFPI in endothelial cells and that androgen upregulated both *TFPI* and *C6ORF105*. Thus, the novel gene that can be regulated by androgen was named *androgen-dependent TFPI-regulating protein* (*ADTRP*) [3]. Later studies established a link between *ADTRP* and CAD, which is a leading cause of death worldwide induced by both genetic and environmental factors [11,12,13]. A genome-wide association study (GWAS) with a Chinese cohort revealed that the single nucleotide polymorphism (SNP), rs6903956, residing in the *ADTRP* locus was significantly associated with CAD, where a minor risk A allele further reduced *ADTRP* expression and elevated CAD risk [14]. The mechanism for this phenomenon was documented by chromatin immunoprecipitation and dual-luciferase assays, in which the GATA transcription factor preferentially bound the G allele over the A allele to upregulate *ADTRP* levels [15]. Moreover, several other groups also confirmed the GWAS results in different Chinese cohorts [16,17], and it was thus proposed that the circulating ADTRP concentration is a better marker for diagnosing CAD than TNF-6, IL-6, or hs-CRP [17]. Similar to its effects on *AIG1*, androgen modulates the expression level of *ADTRP* by directly binding the half androgen-response element within +324 bp of the *ADTRP* transcription start site (TSS) [18]. Upon binding, upregulated ADTRP protein can induce the expression of the transcription factor POU1F1, which is then recruited to the promoter region of *TFPI* and elevates its level [19]. TFPI subsequently activates the PI3K/AKT pathway to upregulate MIA3/TANGO1, which can prevent the initiation of atherosclerosis by inhibiting monocyte adhesion to endothelia and transmigration of monocytes across the endothelial wall [20]. Positive feedback regulation of *ADTRP* and *LDLR*/*CD36*/*LOX-1* in endothelial cells was revealed, and the NF-κB and AKT pathways are possibly involved [21]. Additionally, the expression of ADTRP was modulated by PPARγ in a macrophage study [22].

In addition to in vitro and association studies, the discovery of the in vivo roles of *ADTRP* and/or *AIG1* is crucial. The first experiment was thus carried out in zebrafish, in which there are two *hADTRP* homologs, *adtrp1* and *adtrp2* [23]. Knockdown of *adtrp1* reduced the expression of *tfpi*, and *adtrp1* was shown to be involved in the specification of primitive myelopoiesis and definitive hematopoiesis, while *adtrp2* knockdown by different morpholinos did not affect the aforementioned ADTRP- and/or AIG1-related processes [23], indicating the critical roles of *adtrp1*, but not *adtrp2*, in zebrafish development. Furthermore, another study showed that morpholino-mediated knockdown of zebrafish *adtrp1* resulted in vessel development defects in zebrafish embryos [24]. To further characterize the function of *ADTRP* in mammals, a global *Adtrp*-knockout mouse model was established by removing the sequence between exons 2 and 5. These biallelic knockout mice showed embryonic lethality to some degree and displayed defective vasculature [24]. These results demonstrated that *ADTRP* is a crucial factor during vascular development in both zebrafish and mammals. Interestingly, no abnormality was observed in mice with global knockout of *Aig1, Adtrp,* or both using the CRISPR/Cas9 technique. The discrepancy was likely due to the residual activity of mutant *AIG* genes with a deletion of only 13 bp compared to the wild-type allele. In these mice, only a higher concentration of fatty acid esters of hydroxy fatty acids (FAHFAs), which are signaling lipids with anti-inflammatory and antidiabetic activities [25], was detected, demonstrating that *AIG1* and *ADTRP* might be novel FAHFA hydrolases [26,27].

Genetic and molecular characterization of *AIG1* and *ADTRP* provided some basic information regarding the novel protein family. However, the evolutionary relationship between the members of the *AIG* family has yet to be determined. In the current study, vertebrate AIG-related proteins were phylogenetically investigated, and the results showed that the *AIG* family constitutes three major clades, *AIG1*, *ADTRP*, and *AIG-L*. The orthology and paralogy of vertebrate *AIGs* were clarified, which would facilitate the future selection of appropriate genes for modeling human AIGs function. Some evolutionarily conserved sites were identified, and some are the same as those indicated through experimental approaches. In summary, this study revealed some new information regarding the function of the *AIG* family from an evolutionary perspective, which may be very useful for further physiological studies of *AIGs*.

## 2. Materials and Methods

### 2.1. Data Retrieval

Sequences of human *ADTRP* (NP_001137420.1; gene ID: 84830), *AIG1* (NP_001353273.1; gene ID: 51390), mouse *Adtrp* (NP_780626.1; gene ID: 109254), *Aig1* (NP_079722.1; gene ID: 66253), and zebrafish *aigs* (XP_021336569.1; gene ID: 100537455, XP_009293152.1; gene ID: 562261, and NP_001017719.1; gene ID: 550414) were used as queries to BLAST against the non-redundant protein database consisting of chordate, hemichordate, and echinoderm species through several iterations of the Phi-BLAST algorithm [28]. To maintain the validity of the obtained hits, we selected the sequences based on the E-value (with a cutoff threshold of 0.05), conserved domains (consisting only of Far-17a/AIG1), sequence lengths, and removal of redundant/alternatively spliced sequences [29]. All the obtained sequences were named AIGs in our study. In addition, the UCSC genome browsers [30] and Ensembl database [31] were employed to obtain additional information regarding AIGs. When no *AIG* gene was predicted by searching these databases, the genome sequences based on synteny were downloaded and subjected to GenScan [32] and Augustus servers [33]. If neither prediction tool produced a positive hit of an *AIG*, it was concluded that *AIG* was lost in the species. After collection and prediction, a total of 921 entries were available (Supplementary Data 1).

To clearly present the evolutionary events in animals, we chose some representative species for further analysis: primates (*Homo sapiens* and *Macaca mulatta*), rodents (*Mus musculus* and *Rattus norvegicus*), Artiodactyla (*Bos taurus* and *Sus scrofa*), carnivores (*Canis lupus familiaris* and *Felis catus*), Lagomorpha (*Oryctolagus cuniculus*), Chiroptera (*Myotis lucifugus),* Marsupial (*Sarcophilus harrisii*), Proboscidea (*Loxodonta africana*), Monotreme (*Ornithorhynchus anatinus*); birds (*Anas platyrhynchos*, *Gallus gallus*, *Meleagris gallopavo*, and *Taeniopygia guttata*), reptiles (*Anolis carolinensis*, *Chrysemys picta bellii*, and *Gavialis gangeticus*), amphibians (*Xenopus laevis*, and *Xenopus tropicalis*), lampreys (*Petromyzon marinus*), lobe-finned fish (*Latimeria chalumnae*), 2R ray-finned fish (*Lepisosteus oculatus*), 3R ray-finned fishes (*Danio rerio* and *Takifugu rubripes*), 4R ray-finned fishes (*Oncorhynchus mykiss* and *Cyprinus carpio*), Chondrichthyes (*Rhincodon typus* and *Callorhinchus milii*), Hemichordata (*Saccoglossus kowalevskii*), Echinodermata (*Patiria miniata*, *Acanthaster planci*, *Asterias rubens*, *Strongylocentrotus purpuratus*, and *Anneissia japonica*), Cephalochordata (*Branchiostoma belcheri* and *Branchiostoma floridae*), and tunicates (*Ciona intestinalis*). Invertebrates such as Echinodermata, Hemichordata, Urochordata, and Cephalochordata were used as outgroups. The protein and gene sequences from these species were collected for subsequent analyses (Supplementary Data 1).

### 2.2. Phylogenetic and Syntenic Analyses

Multiple sequence alignment (MSA) was performed using Clustal Omega with default parameters [34]. The raw MSA results were first submitted to the Weblogo3 (Version 3.7.4) server to generate a graphical representation of alignment results [35]. Then, the aligned sequences were subjected to trimAl to remove the columns with >20% gaps (parameter -gt 0.8) [36]. Then, the trimmed MSAs were analyzed by ModelTest-NG to select the best models for phylogenetic inference based on BIC (Bayesian information criteria) values [37,38]. Maximal likelihood (ML) and Bayesian approaches were used to build phylogenetic trees using IQ-TREE (version 2.0.3) [39], RAxML-NG (version 1.0.1) [40], and MrBayes (MPI version 3.2.7) [41]. Briefly, the 922 AIG protein sequences (921 deuterostomes and a sequence from *Caenorhabditis elegans* (NP_510364.2)) were used to generate an all-protein tree using the IQ-tree with substitution matrix JTT+G4, 1000× ultrafast bootstrap [42] combined with 1000× aLRT (alternative likelihood ratio test) [43]. Then, all the available vertebrate AIG protein sequences were subjected to IQ-tree using the same parameters to test whether these sequences could be divided into ADTRP, AIG1, or AIG-L. Subsequently, the ML and Bayesian trees based on sequences from representative species were inferred using RAxML-NG and MrBayes, respectively, with a JTT+G4+F substitution matrix for protein trees. DNA sequences (coding sequences) of these species were subjected to RAxML-NG and MrBayes using HKY+G4 and SYM+I+G4 matrices, respectively. One thousand bootstrap analyses were carried out for RAxML-NG, while the parameters for MrBayes were Nruns=2, Nchains=4, Burninfrac=0.25, Diagnfreq=1000, Samplefreq=100, Stoprule=yes, and Stopval=0.01. The substitution matrices used by RAxML-NG for the mammals, birds, reptiles, amphibians, and fish trees were JTT+I+G4, JTT+G4, JTT+F+G4, JTT+G4, and JTT+G4, respectively. The obtained phylogenetic trees were processed and visualized by FigTree (version 1.4.4) and iTOL (version 6.1.2) [44,45].

### 2.3. Microsynteny Analysis

Microsynteny analysis was performed according to our previously published method [29]. In brief, we retrieved annotations of the protein-coding genes adjacent to *ADTRP*/*AIG1*/*AIG-L* from the NCBI and Ensembl databases. The analyzed species included eutherian species, marsupial (*Ornithorhynchus anatinus*), monotreme (*Sarcophilus harrisii*); fishes (*Callorhinchus milii*, *Rhincodon typus*, *Latimeria chalumnae*, *Oncorhynchus mykiss*, *Danio rerio*, *Lepisosteus oculatus*, *Cyprinus carpio*, and *Takifugu rubripes*), reptiles (*Chrysemys picta bellii*, *Gavialis gangeticus*, and *Anolis carolinensis*), amphibians (*Xenopus laevis* and *Xenopus tropicalis*), birds (*Meleagris gallopavo*, *Gallus*, *Anas platyrhynchos*, and *Taeniopygia guttata*), lampreys (*Petromyzon marinus*), and invertebrates (Echinodermata, Hemichordata, *Branchiostoma*, and *Ciona intestinalis*). For species lacking genetic information for *ADTRP*/*AIG1*/*AIG-L*, such as *Rhincodon typus*, in which only contigs include annotation of *ADTRP*/*AIG1*/*AIG-L*, the syntenic organization was inferred according to other closely related species.

### 2.4. Selective Force Analysis

The trimmed MSA results of representative proteins were converted to codon alignments by PAL2NAL [46]. To evaluate the evolutionary selection force on the *AIG* family, we calculated the nonsynonymous substitution rate to synonymous substitution rate ratio (ω, dN/dS) using the maximum likelihood approach. The CodeML program in PAML 4.9j [47] was employed to run the site model and branch-site model [48,49,50]. The codon frequency counting method parameter was set to CF2, in which codon frequencies were calculated from the average nucleotide frequencies at the three codon positions for analysis [51]. The parameter "cleandata" was set to 0 to retain alignment gaps and prevent loss of genetic information.

Firstly, site model analysis was implemented with model=0 parameter in CodeML. The parameters concerning M0, M1a, M2a, M7, M8, and M8a were evaluated, accordingly (Table S2). After that, likelihood ratio tests (LRTs) were performed to test the utilities of M1a-M2a, M7-M8, and M8a-M8 pairs, respectively.

Then, branch-site model tests were performed using either mammalian ADTRP or AIG1 clade as foreground. The null and alternative models (assuming that the ω ratio was not changed in any branches and that the ω ratio was changed only in foreground branches, respectively) evaluation were performed through likelihood ratio tests, which compare twice the log-likelihood difference of selection and neutral model (2ΔlnL) to values obtained from a χ^2^ distribution with a degree of freedom equivalent to the difference between the parameter numbers of the two models.

If the likelihood ratio tests of the site model or branch-site model were significant, the Bayes Empirical Bayes (BEB) approach [52] was performed to calculate the posterior probability for each positively selected site, which was determined by ω > 1 and posterior probability greater than 0.95.

Positively selected site identification was also performed in MrBayes to verify conclusions drawn from the results obtained with the CodeML program [9]. The SYM+I+G4 substitution matrix was used in the analysis. Thus, the parameters used were as follows: lset nucmodel = codon omegavar = ny98 nst = 6 rates = invgamma; prset revmatpr = Dirichlet (1,1,1,1,1,1) statefreqpr = fixed(equal) shapepr = uniform(0.1, 50) pinvarpr = uniform(0.1).

## 3. Results and Discussion

### 3.1. The AIG Family in Vertebrates Is Composed of Three Members

An initial preliminary phylogenetic study was performed using AIG domain-containing sequences from zebrafish, bovines, chickens, humans, mice, and rats. In this study, all analyzed tetrapods contained two *AIG* paralogs, while zebrafish had three. *zadtrp1* seemed to be an outgroup of *AIG1*s and *ADTRPs*, and *zadtrp2* was an outgroup of all AIG domain-containing genes, contrary to previous reports indicating that *zadtrp1* is an ortholog of human *ADTRP* [23,24]. This outcome prompted us to investigate how many paralogs are in vertebrate genomes and from where they originate. To this end, human, mouse, and zebrafish sequences were utilized to BLAST against a non-redundant protein database of all living organisms. However, the initial search returned hundreds, if not thousands, of hits of AIG domain-containing sequences spanning from microorganisms to humans. Moreover, no AIG domain-bearing genes were found in plants, indicating that the genes might have been replaced or eliminated by plants. Due to the vast number of *AIG*-related genes, we narrowed our scope to focus only on vertebrates, as the function of AIG domain-containing genes is not yet clear.

**Figure 1:**
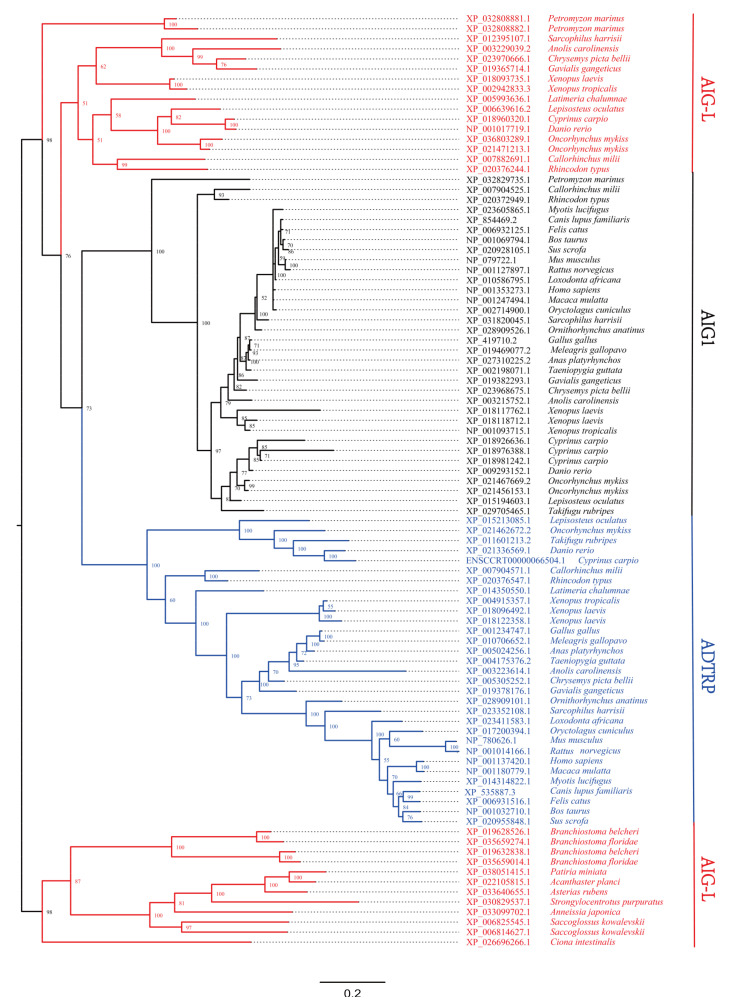
Phylogenetic analysis of the AIG protein sequences in representative species using Bayesian methods. The Bayesian posterior probabilities (×100) are shown for each node. The phylogenetic tree shows a topology with three major clades present in vertebrates. ADTRP, AIG1, and AIG-L are highlighted in blue, black, and red, respectively. Invertebrate AIG-L, which is in red, was used to generate an outgroup. Three similar phylogenetic trees generated using either Bayesian or maximum likelihood approaches are shown in Figures S1–S3.

Representative species from each class of vertebrates were selected, and AIG domain-containing proteins and genes were retrieved from the databases. Maximum likelihood (ML) and Bayesian approaches were employed to construct phylogenetic trees, with sequences from Urochordata, Cephalochordata, Hemichordata, and Echinodermata forming the outgroup. All the trees represent three major clades (Figure 1 and Figures S1–S3), which we named *ADTRP*, *AIG1*, and *AIG-L*. To extrapolate the phylogenetic results, all the retrieved AIG protein sequences with a sequence in *Caenorhabditis elegans* (NP_510364.2) were utilized to build an ML tree using IQ-TREE [39]. The result in Figure S4 indicated that, when rooting invertebrate AIGs, vertebrate AIGs could be divided into the same three major clades. In these trees, AIG-L was found to be paraphyletic, ranging from outgroup species (including the sequence from *Caenorhabditis elegans*) to mammals, indicating that AIG-L might be the ancestral AIG. Thus, to draw a reliable conclusion regarding the AIG classification in vertebrates, another phylogenetic tree was generated with all available vertebrate AIG protein sequences. The unrooted radial tree showing aLRT [43] and UF-boot [42] values (77.3/75, 98.6/100, and 98.6/100 for AIG-L, ADTRP, and AIG1, respectively) supports the supposition that the AIG family in vertebrates consists of AIG-L, AIG1, and ADTRP (Figure S5). 

**Figure 2:**
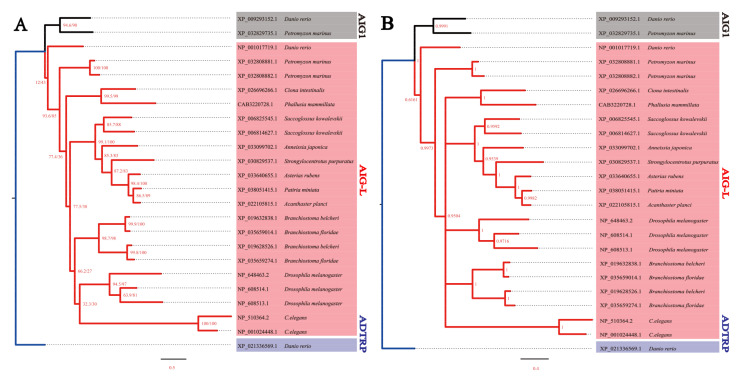
Preliminary phylogenetic classification of invertebrate AIG-Ls using ML (**A**) and Bayesian approaches (**B**). The aLRT/UF-boot values are labeled on each node in A, while the Bayesian posterior probabilities are shown for each node in B. The substitution matrices of mtInv+G4+F and JTT+G4+F were utilized for IQ-tree and MrBayes, respectively. Both trees rooted with zebrafish ADTRP. ADTRP, AIG1, and AIG-L are highlighted in blue, black, and red, respectively. Invertebrate AIG-Ls display a phylum-specific diversification pattern.

To figure out whether invertebrate species embody the same classification of AIGs, a preliminary analysis was carried out using AIG sequences from available invertebrate chordates, hemichordates, echinoderms, zebrafish, lampreys (*Petromyzon marinus*), nematode (*Caenorhabditis elegans*: NP_510364.2 and NP_001024448.1), and arthropod (*Drosophila melanogaster*: NP_608513.1, NP_608514.1, and NP_648463.2). As shown in Figure 2, when zebrafish ADTRP was used as the root, *Petromyzon marinus* and zebrafish AIG1s were grouped together, separating from AIG-Ls. Moreover, the vertebrate AIG-Ls were placed at the base of AIG-L clade, while no one-to-one orthologous relationship is noted between different phyla/subphyla. This result indicates that the diversification of AIGs might be phyla/subphyla specific, and the classification of AIG1, ADTRP, and AIG-L of vertebrates could not be applied to other phyla. Future work concerning more invertebrates will be needed to decipher the evolution of AIGs in other species.

**Figure 3:**
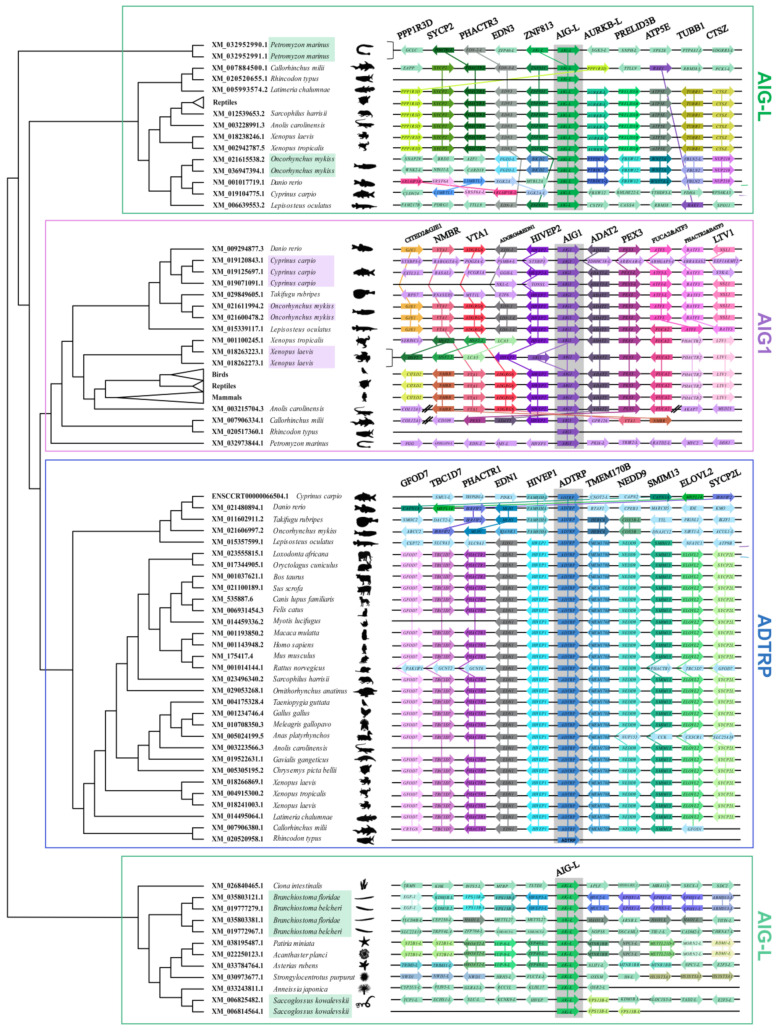
Syntenic analysis of *AIG* genes in representative species. The cladogram was adapted from Figure S2. The genes adjacent to *AIGs* (4–5 genes upstream/downstream) are shown as arrows with the arrowheads indicating the direction of orientation of the transcription of each gene.

Next, *AIGs* from *Petromyzon marinus* were first used to deduce the evolution of *AIG* genes. *Petromyzon marinus* has three copies of *AIG* genes, two of which were classified as *AIG-L* in a tandem duplication manner (Figure 3). The remaining copy was identified as *AIG1*. It is widely accepted that two rounds of whole-genome duplication (WGD) that took place 450 million years ago (Mya) gave rise to the current vertebrate genomes [53]. However, the time of duplication is still debated. There are at least three major hypotheses explaining the timing of WGD in vertebrates. In the first hypothesis, two rounds of WGD occurred after the split of cyclostomes and gnathostomes, which resulted in the formation of a single cyclostome gene with four gnathostome homologs [54,55]. Our result is apparently contradictory to this hypothesis. In our case, the cartilaginous fish contained three *AIG* copies in synteny, similar to that in *Petromyzon marinus*, where *AIG*s were adjacent to either *EDN-like* genes or *HIVEP-like* genes (*Amblyraja radiata* has relatively complete synteny information, while the genome information of *Callorhinchus milii* is poorly annotated, and no synteny could be determined for *Rhincodon typus* (Figure 3 and Supplementary Data 2)), indicating a common duplication event. The second hypothesis points out that 1R WGD occurred in the common ancestor of vertebrates, while 2R WGD occurred only in gnathostomes [56,57,58,59,60]. This mechanism is supported by recent studies on amphioxus genome evolution. Putnam et al. first sequenced the amphioxus genome and constructed ancestral chordate linkage groups, from which the orthologous genes could be identified and duplication patterns can be inferred. Additionally, through the analysis of chordate linkage group and vertebrate genomes, the 1R and 2R WGD events could be verified in terms of quadruple conserved synteny [61]. Later, through the integration of new sequencing data, Simakov et al. updated the chordate linkage groups and demonstrated that the 2R WGD, resulting from interspecies hybridization of two extinct vertebrates, occurred only in gnathostomes [62]. This hypothesis seems to be the best explanation of our results, showing that the 1R WGD produced the *AIG-L* gene and the *AIG1* gene in the common ancestor of vertebrates. Then, 2R WGD resulted in the formation of *AIG1* and *ADTRP* in gnathostomes. Thus, future detailed studies involving the location of *AIG* family in chordate linkage groups should be conducted. Besides the mentioned hypotheses, there is still a third opinion regarding the timing of 2R WGD, according to which 2R WGD occurred before the split of agnathans and gnathostomes [63,64]. In this scenario, the three *AIG* copies in Chondrichthyes would be the result of two rounds of WGD, and the *ADTRP* copy in lampreys may have been lost due to pseudogenization.

### 3.2. Tetrapod AIG Evolution

As shown in Figure 1, among mammalian and avian species, only *Sarcophilus harrisii* has a copy of the *AIG-L* gene. To determine whether *AIG-L* was preserved in mammalian genomes, another phylogenetic analysis was performed using all the available mammalian AIG protein sequences. As shown in Figure S6, almost all mammals contain two *AIG* paralogs of *AIG1* and *ADTRP*, except for two species of metatherians, *Sarcophilus harrisii* and *Phascolarctos cinereus*, which preserve *AIG-L*. As shown by the synteny analysis, *AIG-L* in all tetrapod species resides in a relatively conserved synteny group (Figure 3 and Supplementary Data 2). Thus, it is likely that most mammals lost the *AIG-L* gene during evolution, while only a few metatherians preserved a copy.

Next, all sequences from birds were also analyzed to determine whether *AIG-L* is lost in avian species. Phylogenetic groups combined with synteny pointed out that *Apteryx mantelli mantelli*, *Apteryx rowi*, *Dromaius novaehollandiae*, *Nothoprocta perdicaria*, *Struthio camelus australis*, and *Tinamus guttatus* have additional *AIG-L* copies compared to other bird species (Figure S7). All *AIG-L*-bearing birds belong to Palaeognathae, which split from Neognathae at approximately 110 Mya, indicating that the loss of *AIG-L* in Neognathae occurred after the divergence of the two major bird clades [65]. It is not clear whether the presence of an additional *AIG* copy is related to the gigantism of Palaeognathae, which needs to be further investigated. Additionally, *AIG-L* is lost in the majority of mammalian and avian species; however, synteny is maintained. Upon searches in several databases, no pseudogenes were discovered in the intervening sequence between *PRELID3B* and *ZNF831*. Thus, to determine whether the species without the *AIG-L* indeed lost the gene, Genscan [32] and Augustus [33] were used to predict the existence of *AIG-L* in a locus that is located in the intervening sequence between *ZNF831* and *PRELID3B* in *Homo sapiens*, *Mus musculus*, *Sus scrofa*, proto-/metatherian, *Gallus gallus*, *Taeniopygia guttata*, *Chrysemys picta bellii*, *Gavialis gangeticus*, *Anolis carolinensis*, *Xenopus tropicalis*, *Meleagris gallopavo*, *Anas platyrhynchos*, Palaeognathae, and *Latimeria chalumnae*. No *AIG* was found in eutherians or Neognathae, but several retrotransposon elements were identified across the regions. Although several studies have reported that retrotransposons can induce gene loss, further detailed analyses are required to determine whether there is a causative reason for the absence of *AIG-L* in eutherians, prototherians, and Neognathae and the existence of retrotransposons [66,67,68].

All reptilian species, except for *Platysternon megacephalum*, *Python bivittatus*, *Ophiophagus hannah*, and *Varanus komodoensis*, for which there is poor genome annotation, contain three *AIG* copies, indicating that *AIGs* are essential for the biology of reptiles. In contrast, analysis of amphibian species showed that of the six amphibian species with currently available genome data, Batrachia has all three major *AIG* members, while Gymnophiona lost *ADTRP* (Figures S8 and S9). In contrast to other amphibians, *Xenopus laevis* contains two *ADTRP* copies, *ADTRP.S* and *ADTRP.L*. It has been suggested that a recent tetraploidization occurred 40 Mya after the divergence of *Xenopus laevis* and *Xenopus tropicalis* [69,70], resulting in the formation of gene triplets in the two species [71]. Thus, it is reasonable that the two *Xenopus laevis ADTRP* duplicates were retained in the genome, while the incomplete rediploidization event removed one copy of *AIG1* and *AIG-L*. However, the two *AIG1s* in the current *Xenopus laevis* are likely due to a local duplication event, not whole-genome duplication (Figure 3).

### 3.3. Ray-Finned Fishes

An analysis of ray-finned fish genomes led to 277 hits of AIG sequences, most of which belonged to Percomorphaceae, Salmonidae, and Cyprinidae (Supplementary Data 1). As shown in Figure 1, the representative ray-finned fish included in this study contain various copies of *AIG* genes. Moreover, *Takifugu rubripes* (Percomorphaceae) does not have *AIG-L*; *Oncorhynchus mykiss* (Salmonidae) has two *AIG1s*, two *AIG-Ls*, and one *ADTRP*; and *Cyprinus carpio* has three *AIG1s*, 1 *ADTRP*, and 1 *AIG-L*. These results indicate that the *AIG* family underwent several different duplications, losses, and rearrangements in these teleosts, possibly due to 3R or 4R WGD events [72,73,74,75]. The other interesting inference obtained through our phylogenetic analysis suggests that the three *AIG* copies in zebrafish, originally named *adtrp1*, *adtrp2*, and *aig1*, are phylogenetically clustered with *AIG-L*, *ADTRP*, and *AIG1* clades, respectively. Therefore, they were renamed *aig-l*, *adtrp*, and *aig1*. Hence, the previous *adtrp1* is actually paralogous to *hADTRP*. According to functional studies of zebrafish and mice, the paralogs *zaig-l* and *mAdtrp* play similar roles in vascular development and can regulate TFPI expression. However, the mammalian *ADTRP* ortholog *zadtrp* does not exert a similar function [23,24], indicating no functional redundancy of *zadtrp* and *zaig-l*. Gene duplication is the driving force for speciation and adaptation [76]. Most duplicated gene copies underwent different selection forces, resulting in neofunctionalization, subfunctionalization, or pseudogenization [77,78,79]. Thus, a possible explanation for the experimental outcomes is that after duplication of ancestral *AIG*, vascular development-related function was preserved in tetrapod *ADTRPs* and ray-finned fish *AIG-Ls*.

**Figure 4:**
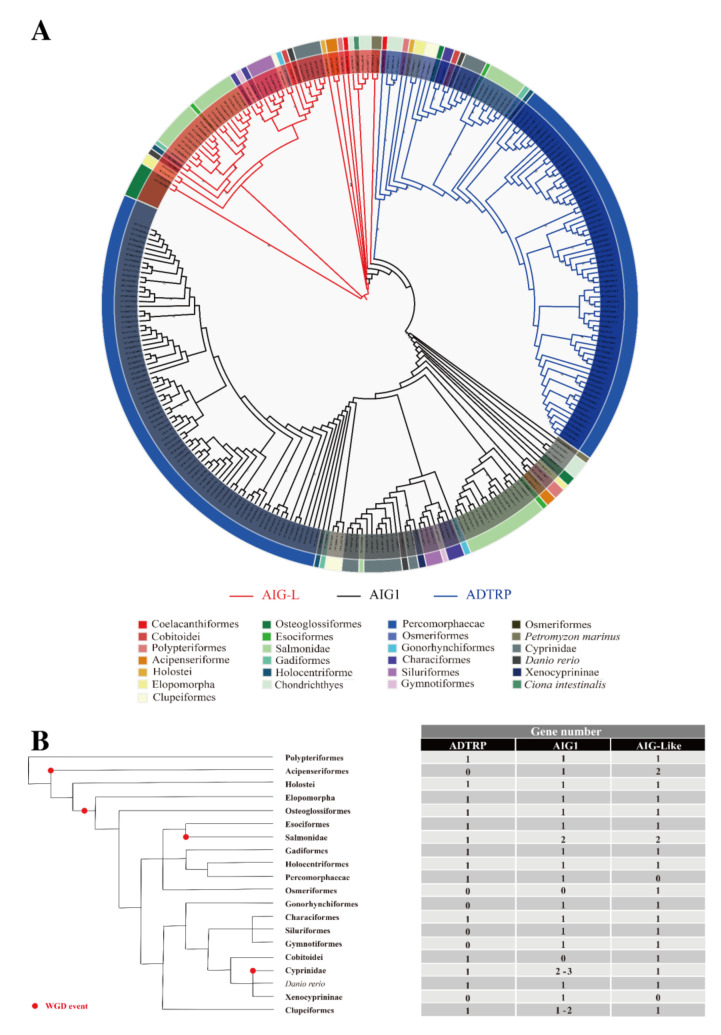
The classification of fish AIG proteins. (**A**) Phylogenetic analysis of AIG proteins in Urochordate, Chondrichthyes, and Osteichthyes using the ML approach with RAxML-NG. The branches with bootstrap values > 60 are labeled in the figure. AIG1-L, AIG1, and ADTRP branches are highlighted in red, black, and blue, respectively. Additionally, AIG-L from *Ciona intestinalis* was grouped with other AIG-Ls. (**B**) Gene numbers of each *AIG* member of each fish taxonomic unit were estimated and presented as a fish cladogram. The WGD events are denoted in the cladogram by red dots.

To obtain a more comprehensive view of *AIG* evolution in fish, another phylogenetic tree was established using Urochordate, Chondrichthyes, and Osteichthyes *AIGs* (Figure 4). This tree gave similar results to those presented in Figure 1 and Figure S4: the *AIG-L* clade is paraphyletic, while *AIG1* and *ADTRP* are monophyletic. Detailed analysis of the old or basal Actinopterygian, Polypteriformes, and Acipenseriformes showed that both species (*Acipenser ruthenus* and *Erpetoichthys calabaricus*) have four copies of *AIGs* with three potential protein-coding genes and one pseudogene (Supplementary Data 1), and another 2R ray-finned fish, spotted gar (*Lepisosteus oculatus*), has three *AIGs,* similar to those in Chondrichthyes and tetrapods. A clustering analysis revealed that *Acipenser ruthenus* has one *AIG1* and two *AIG-Ls*, and the other two basal Actinopterygian species have one *ADTRP*, one *AIG1*, and one *AIG-L*. The differences between *Acipenser ruthenus* and the two other basal fish might be explained by *Acipenser ruthenus*-specific genome duplication [80].

Approximately 320 Mya, another teleost-specific WGD, occurred after the divergence of the Holostei and Teleostei, and rediploidization resulted in most orders of teleosts maintaining three copies of *AIGs* [72,73,81], such as Anguilliformes, Elopiformes, Osteoglossiformes, Esociformes, Gadiformes, Characiformes, and Holocentriformes. There are also exceptions to *AIG* evolution in other teleost orders and families. Of the Percomorphaceae, which includes Anabantiformes, Synbranchiformes, Batrachoidiformes, Carangiformes, Pleuronectiformes, Centrarchiformes, Labriformes, Perciformes, Spariformes, Tetraodontiformes, Gobiiformes, Kurtiformes, Beloniformes, Cyprinodontiformes, Blenniiformes, Cichliformes, and Syngnathiformes in our study, only *AIG1* and *ADTRP* were maintained without the presence of *AIG-L*. Other exceptions were found in Salmonidae and Cyprinidae, in which 4R WGD occurred independently [74,82,83,84,85]. Thus, most Salmonidae species consistently contain five *AIG* copies with one *ADTRP*, two *AIG1s*, and two *AIG-Ls*, resulting from a common auto-tetraploidization event ~100 Mya [74,83,86]. On the other hand, Cyprinidae species have only one *ADTRP* (*Sinocyclocheilus anshuiensis* contains one *ADTRP* pseudogene according to synteny analysis) and one *AIG-L* and 2–3 copies of *AIG1*. Even the closely related *Carassius auratus* (goldfish) and *Cyprinus carpio* (common carp), whose common ancestor underwent WGD ~8 to 12 Mya, have different *AIG1* copies [75,87].

### 3.4. Selection Force Analysis

After gene duplications, the duplicated copies of the *AIG* genes may experience purifying selection or adaptive evolution, which make them functionally conserved or diversified, respectively. To test which evolutionary selection predominated, multiple sequence alignments of AIG protein sequences were converted to codon alignments using PAL2NAL [46]. Then, overall dN/dS values of the *AIG* family were evaluated using the Nei-Gojobori method in MEGA X [88,89] and the M0 model in CodeML [90,91]. Both approaches predicted a predominant purifying force (dN/dS < 1) acting upon the whole *AIG* family (*p*-value < 0.01 for the alternative hypothesis of dN/dS < 1 as indicated by MEGA X; ω = 0.22 indicated by the M0 model of CodeML). It is common, and possibly always true, that many amino acid residues in a given protein sequence undergo strong purifying selection, making the average dN/dS ratio less than 1 [92,93]. Thus, it is more reasonable to test selection acting upon individual amino acids [94]. Next, MrBayes and a site/branch-site model in CodeML were employed to identify potential positively selected sites. The results of MrBayes showed no positively selected sites (Table S1). For the site model implemented by CodeML, no specific site experiencing positive selection was identified either (Tables S2–S3). Thus, the branch-site model was then used to test whether these sites could be found in mammalian ADTRP or AIG1. Again, although some codons with dN/dS > 1 were identified, the Bayesian Empirical Bayes (BEB) values failed to show significance, indicating that no positively selected sites were discovered (Table S4). These results suggest that the evolutionary processes of the vertebrate AIG family were conserved.

**Figure 5:**
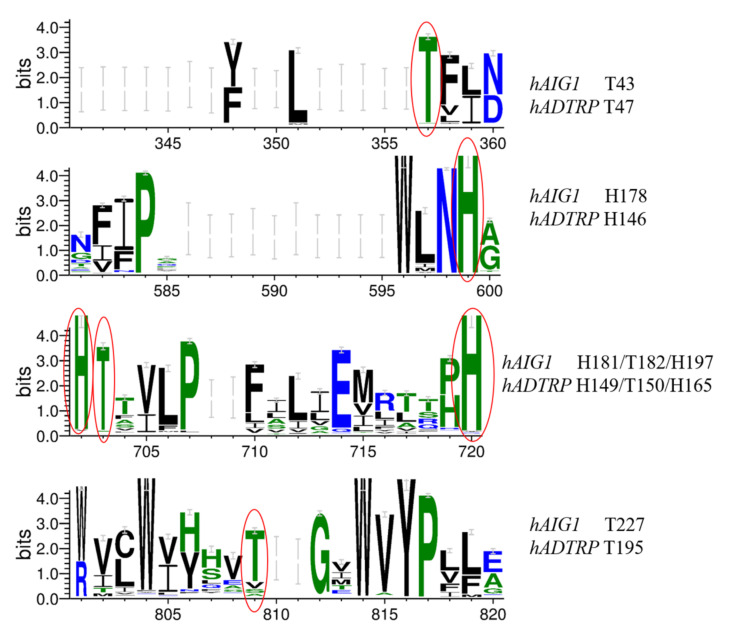
Graphical representation of the conserved amino acid sites across all available AIG protein sequences. The conserved sites are marked with red ellipses.

In two recent functional studies, *AIG1* and *ADTRP* were classified as atypical integral membrane hydrolases of FAHFAs, where genetic ablation or pharmacological inhibition of either protein could elicit an increase in endogenous FAHFA without any cardiovascular defects [26,27]. Mutation of two evolutionarily conserved amino acid residues (hAIG1 Thr43/His181 and hADTRP Thr47/His149) abolished enzyme activity [26]. In our MSA, these two residues were conserved across most vertebrate species. Four additional conserved histidine and threonine sites were also identified in hADTRP (His146/165 and Thr150/195) and hAIG1 (His178/197 and Thr182/227) (Figure 5 and Figure S10). However, experimental approaches have already proven that mutations of hAIG1 Thr182, His197, and Thr227 to alanine did not affect the hydrolase activity of hAIG1, leaving only His178 of hAIG1 to be determined to play a role in enzymatic activity [26]. Therefore, in our study, the members of the *AIG* family show a conserved evolutionary pattern and might have a conserved function.

## 4. Conclusions

Our study, for the first time, characterized the vertebrate *AIG* family from an evolutionary perspective. Through phylogenetic and syntenic analyses, vertebrate *AIGs* were categorized into three major groups, *ADTRP*, *AIG1*, and *AIG-L*. We also proved that vertebrate *AIGs* might evolve from invertebrate *AIG-L* genes with the expansion that occurred in the common ancestor of agnathans and gnathostomes. During evolution, some taxonomic units might have lost or gained *AIGs*; however, extant *AIGs* may have a conserved function because of purifying selection. Importantly, our study clearly presented human, mouse, and zebrafish *ADTRP*/*AIG* orthologs, which lays the foundation for future reliable molecular characterization of *AIGs*.

## Data Availability

All data are available online or upon request.

## Supplementary Materials

The following files are available online at https://www.mdpi.com/article/10.3390/genes12081190/s1. Figures S1-S3. Phylogenetic analysis of the AIG protein (S1) and CDS (S2 and S3) sequences in representative species using ML (S1 and S2) and Bayesian methods (S3). The bootstrap values (S1 and S2) and Bayesian posterior probabilities (S3) are shown for each node. All phylogenetic trees, except for the position of *Peteromyzon marinus* AIG-Ls in S3, show a similar topology with three major clades present in vertebrates. ADTRP, AIG1 and AIG-L are highlighted in blue, black, and red, respectively. Invertebrate AIG-L, which is in red, was used to generate an outgroup. Figure S4. Phylogenetic representation of AIG protein sequences in all available species using the ML approach. The rooted tree shows the classification of the AIGs, which was supported by high aLRT (numerators) and bootstrap values (denominators). The values for ADTRP, AIG1, and AIG-L are 98.2/100, 98.9/100, and 89.1/94, respectively. Closely related species were grouped together in collapsed triangles. The invertebrate AIGs were used as an outgroup. Figure S5. Phylogenetic representation of AIG protein sequences in all available vertebrates using the ML approach. The radial tree generated was supported by high aLRT (numerators) and bootstrap values (denominators). The values for ADTRP, AIG1, and AIG-L are 98.6/100, 98.6/100, and 77.3/75, respectively. Figures S6-S9. Phylogenetic trees generated using the ML approach for mammals (S6), birds (S7), reptiles (S8), and amphibians (S9). ADTRP, AIG1, and AIG-L are labeled by blue, black, and red, respectively. AIG-L from *Ciona intestinalis* was used as the outgroup. Figure S10. Graphical representation of the conserved amino acid sites across all available AIG protein sequences. Supplementary Data 1. The list of all AIG sequences and related information. Supplementary Data 2. The complete synteny information of the *AIGs* in this study. Table S1. Positively selected sites identified by MrBayes. Table S2. Parameter estimates for site models of AIG. Table S3. Site model tests of AIG. Table S4. Branch model generated by CodeML.
