# Supplementary material for: Molecular Phylogenetic Analysis of the AIG Family in Vertebrates
Source: Genes (Basel). 2021 Jul 30;12(8):1190. doi: 10.3390/genes12081190 (PMC8394805; doi:10.3390/genes12081190)
Supplement: Supplementary file 1 [file genes-12-01190-s001.zip › genes-1297795-Supplementary-Materials-Final/genes-1297795-Supplementary-Figures.pdf]

genes

MDPI

Communication

Molecular Phylogenetic Analysis of The *AIG* Family in Vertebrates

Yuqi Huang [1,†], Minghao Sun [2,†], Lenan Zhuang [2,*] and Jin He [1,*]

Supplementary Figures

Figures S1-S3. Phylogenetic analysis of the AIG protein (**S1**) and CDS (**S2** and **S3**) sequences in representative species using ML (**S1 and S2**) and Bayesian methods (**S3**). The bootstrap values (**S1** and **S2**) and Bayesian posterior probabilities (**S3**) are shown for each node. All phylogenetic trees, except for the position of *Peteromyzon marinus* AIG-Ls in S3, show a similar topology with three major clades present in vertebrates. ADTRP, AIG1 and AIG-L are highlighted in blue, black, and red, respectively. Invertebrate AIG-L, which is in red, was used to generate an outgroup.

Figure S1

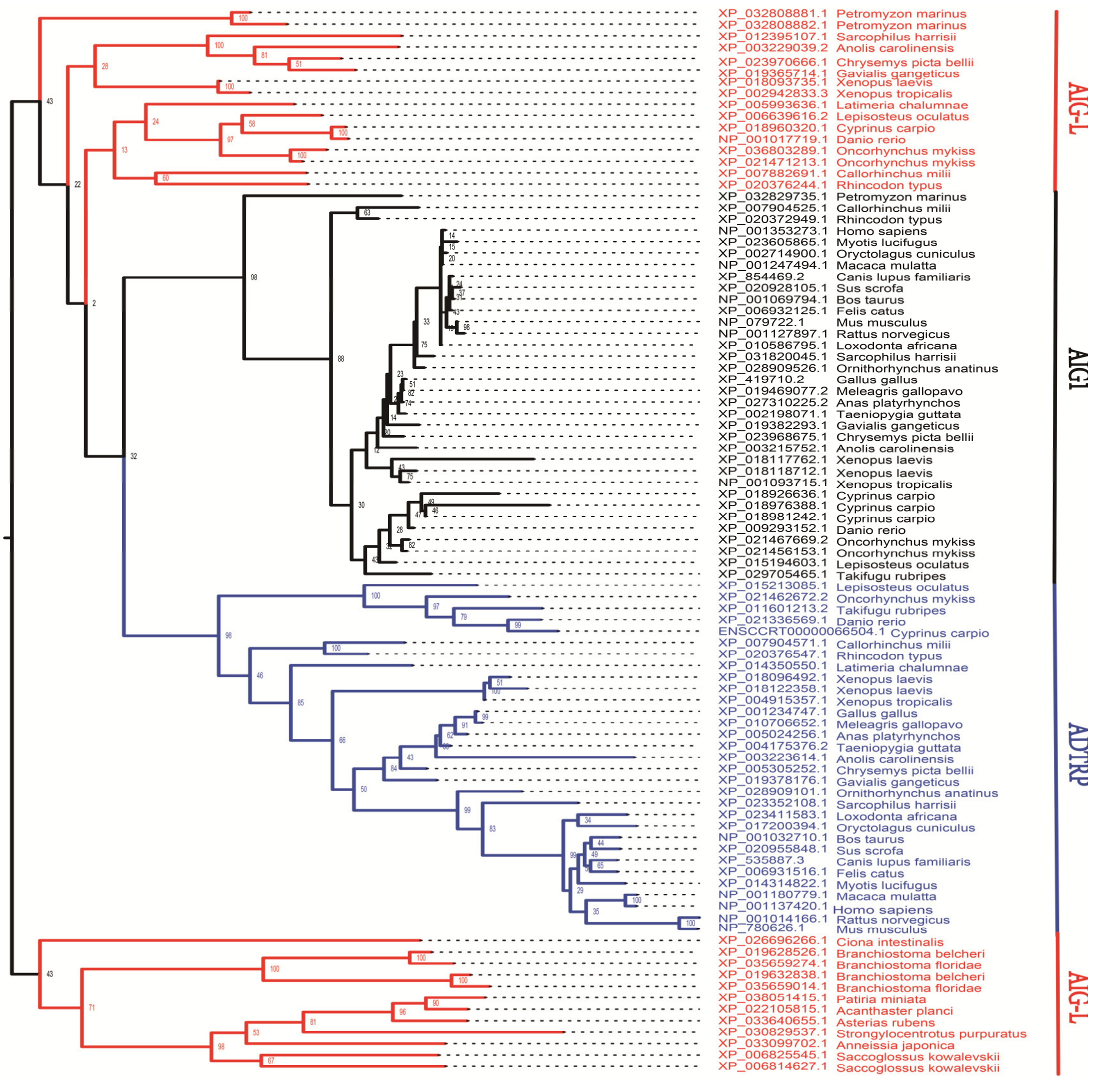

Figure S2

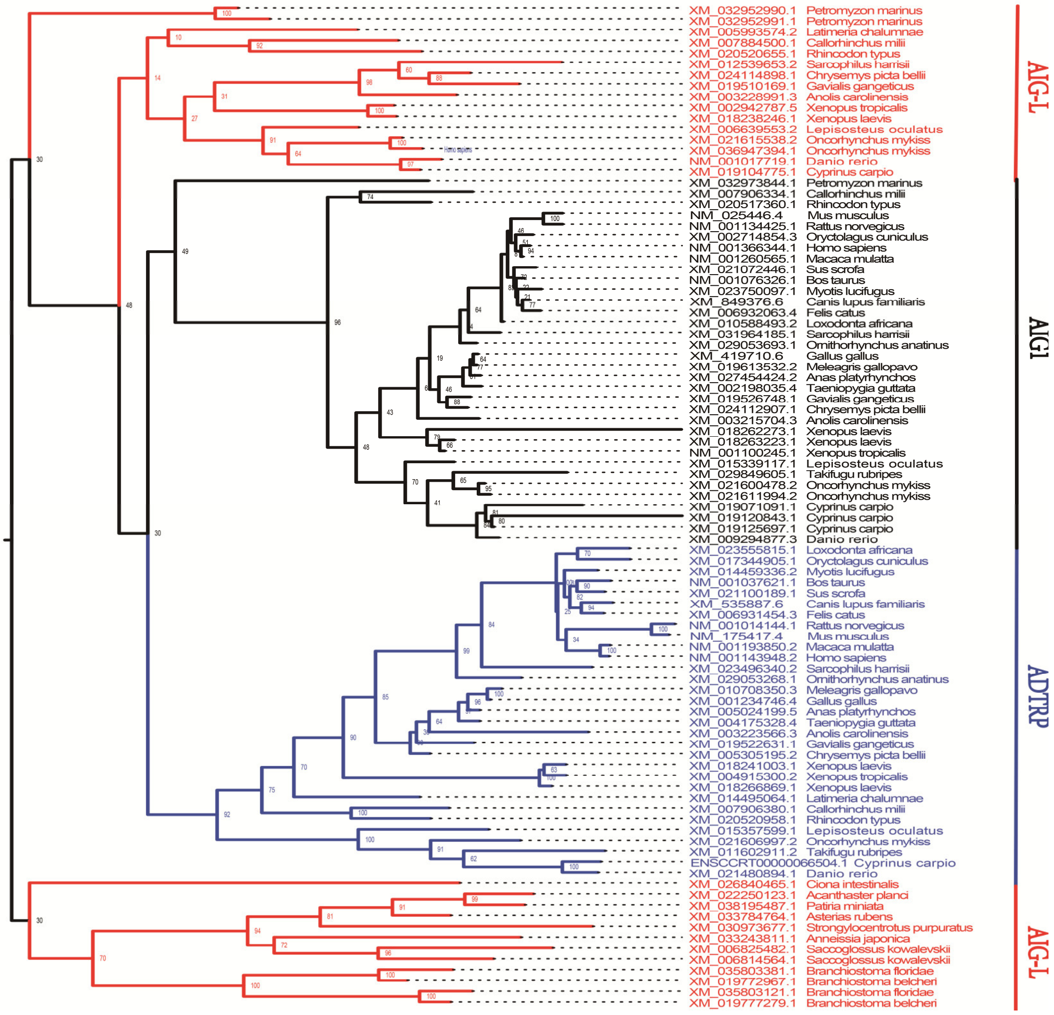

XM_032952990.1 Petromyzon marinus
XM_032952991.1 Petromyzon marinus
XM_005993574.2 Latimeria chalumnae
XM_007884500.1 Callorhinchus milii
XM_020520655.1 Rhincodon typus
XM_012539653.2 Sarcophilus harrisii
XM_024114898.1 Chrysemys picta bellii
XM_019510169.1 Gavialis gangeticus
XM_003228991.3 Anolis carolinensis
XM_002942787.5 Xenopus tropicalis
XM_018238246.1 Xenopus laevis
XM_006639553.2 Lepisosteus oculatus
XM_021615538.2 Oncorhynchus mykiss
XM_036947394.1 Oncorhynchus mykiss
NM_001017719.1 Danio rerio
XM_019104775.1 Cyprinus carpio
XM_032973844.1 Petromyzon marinus
XM_007906334.1 Callorhinchus milii
XM_020517360.1 Rhincodon typus
NM_025446.4 Mus musculus
NM_001134425.1 Rattus norvegicus
XM_002714854.3 Oryctolagus cuniculus
NM_001366344.1 Homo sapiens
NM_001260565.1 Macaca mulatta
XM_021072446.1 Sus scrofa
NM_001076326.1 Bos taurus
XM_023750097.1 Myotis lucifugus
XM_849376.6 Canis lupus familiaris
XM_006932063.4 Felis catus
XM_010588493.2 Loxodonta africana
XM_031964185.1 Sarcophilus harrisii
XM_029053693.1 Ornithorhynchus anatinus
XM_419710.6 Gallus gallus
XM_019613532.2 Meleagris gallopavo
XM_027454424.2 Anas platyrhynchos
XM_002198035.4 Taeniopygia guttata
XM_019526748.1 Gavialis gangeticus
XM_024112907.1 Chrysemys picta bellii
XM_003215704.3 Anolis carolinensis
XM_018262273.1 Xenopus laevis
XM_018263223.1 Xenopus laevis
NM_001100245.1 Xenopus tropicalis
XM_015339117.1 Lepisosteus oculatus
XM_029849605.1 Takifugu rubripes
XM_021600478.2 Oncorhynchus mykiss
XM_021611994.2 Oncorhynchus mykiss
XM_019071091.1 Cyprinus carpio
XM_019120843.1 Cyprinus carpio
XM_019125697.1 Cyprinus carpio
XM_009294877.3 Danio rerio
XM_023555815.1 Loxodonta africana
XM_017344905.1 Oryctolagus cuniculus
XM_014459336.2 Myotis lucifugus
NM_001037621.1 Bos taurus
XM_021100189.1 Sus scrofa
XM_535887.6 Canis lupus familiaris
XM_006931454.3 Felis catus
NM_001014144.1 Rattus norvegicus
NM_175417.4 Mus musculus
NM_001193850.2 Macaca mulatta
NM_001143948.2 Homo sapiens
XM_023496340.2 Sarcophilus harrisii
XM_029053268.1 Ornithorhynchus anatinus
XM_010708350.3 Meleagris gallopavo
XM_001234746.4 Gallus gallus
XM_005024199.5 Anas platyrhynchos
XM_004175328.4 Taeniopygia guttata
XM_003223566.3 Anolis carolinensis
XM_019522631.1 Gavialis gangeticus
XM_005305195.2 Chrysemys picta bellii
XM_018241003.1 Xenopus laevis
XM_004915300.2 Xenopus tropicalis
XM_018266869.1 Xenopus laevis
XM_014495064.1 Latimeria chalumnae
XM_007906380.1 Callorhinchus milii
XM_020520958.1 Rhincodon typus
XM_015357599.1 Lepisosteus oculatus
XM_021606997.2 Oncorhynchus mykiss
XM_011602911.2 Takifugu rubripes
ENSCCRT00000066504.1 Cyprinus carpio
XM_021480894.1 Danio rerio
XM_026840465.1 Ciona intestinalis
XM_022250123.1 Acanthaster planci
XM_038195487.1 Patiria miniata
XM_033784764.1 Asterias rubens
XM_030973677.1 Strongylocentrotus purpuratus
XM_033243811.1 Anneissia japonica
XM_006825482.1 Saccoglossus kowalevskii
XM_006814564.1 Saccoglossus kowalevskii
XM_035803381.1 Branchiostoma floridae
XM_019772967.1 Branchiostoma belcheri
XM_035803121.1 Branchiostoma floridae
XM_019777279.1 Branchiostoma belcheri
AIG-L
AIG1
ADTRP
AIG-L

Figure S3

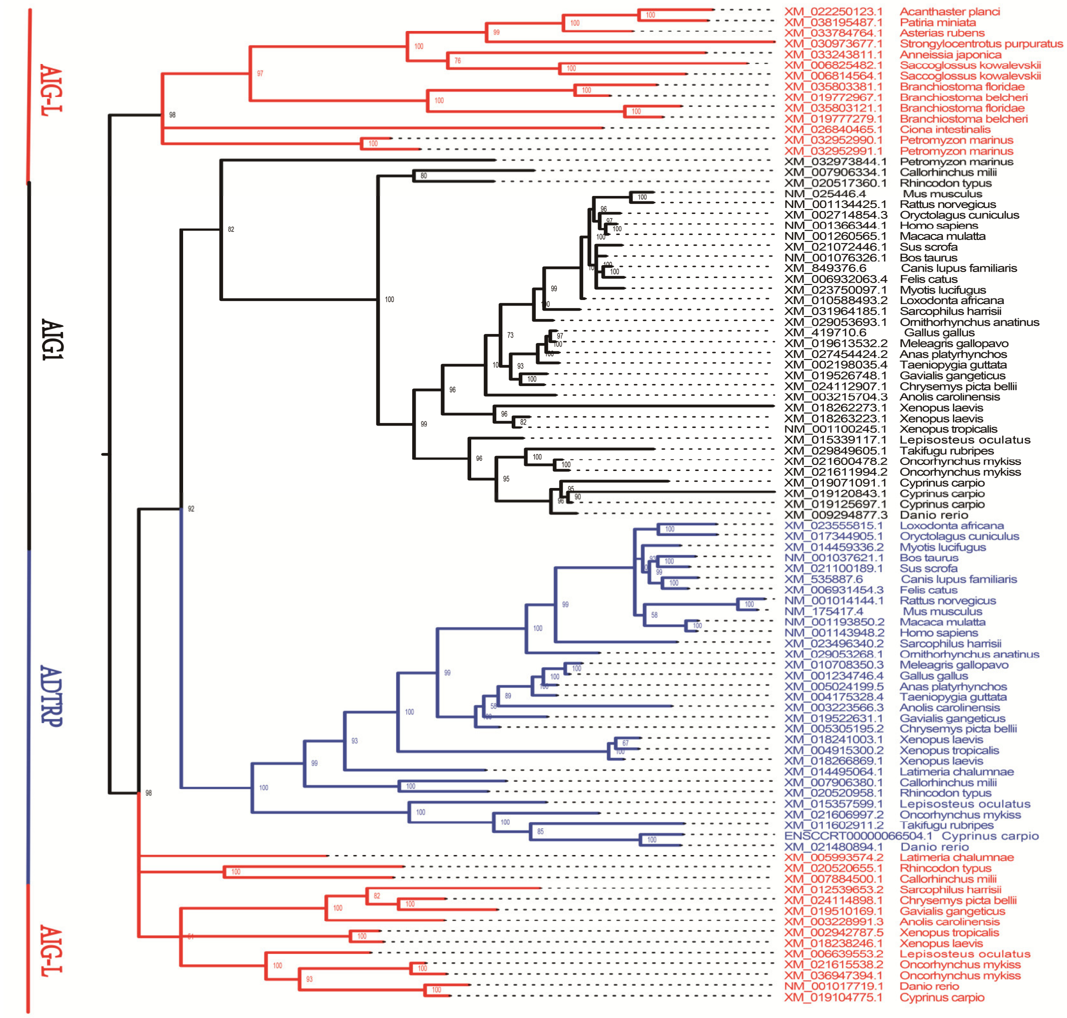
AIG-L
AIG1
ADTRP
AIG-L
XM_022250123.1 Acanthaster planci
XM_038195487.1 Patiria miniata
XM_033784764.1 Asterias rubens
XM_030973677.1 Strongylocentrotus purpuratus
XM_033243811.1 Anneissia japonica
XM_006825482.1 Saccoglossus kowalevskii
XM_006814564.1 Saccoglossus kowalevskii
XM_035803381.1 Branchiostoma floridae
XM_019772967.1 Branchiostoma belcheri
XM_035803121.1 Branchiostoma floridae
XM_019777279.1 Branchiostoma belcheri
XM_026840465.1 Ciona intestinalis
XM_032952990.1 Petromyzon marinus
XM_032952991.1 Petromyzon marinus
XM_032973844.1 Petromyzon marinus
XM_007906334.1 Callorhinchus milii
XM_020517360.1 Rhincodon typus
NM_025446.4 Mus musculus
NM_001134425.1 Rattus norvegicus
XM_002714854.3 Oryctolagus cuniculus
NM_001366344.1 Homo sapiens
NM_001260565.1 Macaca mulatta
XM_021072446.1 Sus scrofa
NM_001076326.1 Bos taurus
XM_849376.6 Canis lupus familiaris
XM_006932063.4 Felis catus
XM_023750097.1 Myotis lucifugus
XM_010588493.2 Loxodonta africana
XM_031964185.1 Sarcophilus harrisii
XM_029053693.1 Ornithorhynchus anatinus
XM_419710.6 Gallus gallus
XM_019613532.2 Meleagris gallopavo
XM_027454424.2 Anas platyrhynchos
XM_002198035.4 Taeniopygia guttata
XM_019526748.1 Gavialis gangeticus
XM_024112907.1 Chrysemys picta bellii
XM_003215704.3 Anolis carolinensis
XM_018262273.1 Xenopus laevis
XM_018263223.1 Xenopus laevis
NM_001100245.1 Xenopus tropicalis
XM_015339117.1 Lepisosteus oculatus
XM_029849605.1 Takifugu rubripes
XM_021600478.2 Oncorhynchus mykiss
XM_021611994.2 Oncorhynchus mykiss
XM_019071091.1 Cyprinus carpio
XM_019120843.1 Cyprinus carpio
XM_019125697.1 Cyprinus carpio
XM_009294877.3 Danio rerio
XM_023555815.1 Loxodonta africana
XM_017344905.1 Oryctolagus cuniculus
XM_014459336.2 Myotis lucifugus
NM_001037621.1 Bos taurus
XM_021100189.1 Sus scrofa
XM_535887.6 Canis lupus familiaris
XM_006931454.3 Felis catus
NM_001014144.1 Rattus norvegicus
NM_175417.4 Mus musculus
NM_001193850.2 Macaca mulatta
NM_001143948.2 Homo sapiens
XM_023496340.2 Sarcophilus harrisii
XM_029053268.1 Ornithorhynchus anatinus
XM_010708350.3 Meleagris gallopavo
XM_001234746.4 Gallus gallus
XM_005024199.5 Anas platyrhynchos
XM_004175328.4 Taeniopygia guttata
XM_003223566.3 Anolis carolinensis
XM_019522631.1 Gavialis gangeticus
XM_005305195.2 Chrysemys picta bellii
XM_018241003.1 Xenopus laevis
XM_004915300.2 Xenopus tropicalis
XM_018266869.1 Xenopus laevis
XM_014495064.1 Latimeria chalumnae
XM_007906380.1 Callorhinchus milii
XM_020520958.1 Rhincodon typus
XM_015357599.1 Lepisosteus oculatus
XM_021606997.2 Oncorhynchus mykiss
XM_011602911.2 Takifugu rubripes
ENSCCRT00000066504.1 Cyprinus carpio
XM_021480894.1 Danio rerio
XM_005993574.2 Latimeria chalumnae
XM_020520655.1 Rhincodon typus
XM_007884500.1 Callorhinchus milii
XM_012539653.2 Sarcophilus harrisii
XM_024114898.1 Chrysemys picta bellii
XM_019510169.1 Gavialis gangeticus
XM_003228991.3 Anolis carolinensis
XM_002942787.5 Xenopus tropicalis
XM_018238246.1 Xenopus laevis
XM_006639553.2 Lepisosteus oculatus
XM_021615538.2 Oncorhynchus mykiss
XM_036947394.1 Oncorhynchus mykiss
NM_001017719.1 Danio rerio
XM_019104775.1 Cyprinus carpio

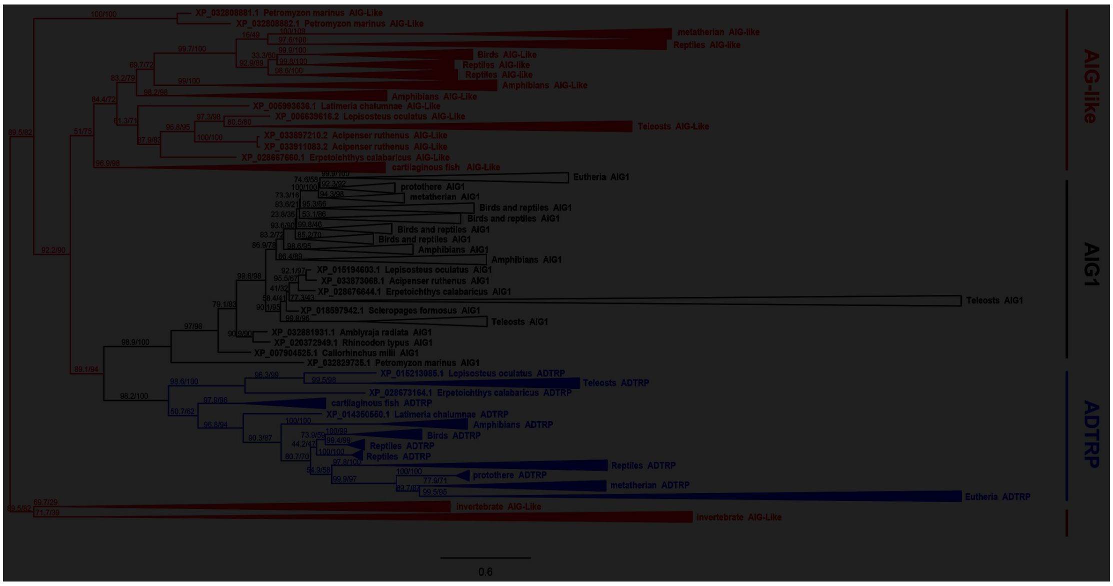

Figure S4. Phylogenetic representation of AIG protein sequences in all available species using the ML approach. The rooted tree shows the classification of the AIGs, which was supported by high aLRT (numerators) and bootstrap values (denominators). The values for ADTRP, AIG1, and AIG-L are 98.2/100, 98.9/100, and 89.1/94, respectively. Closely related species were grouped together in collapsed triangles. The invertebrate AIGs were used as outgroup.

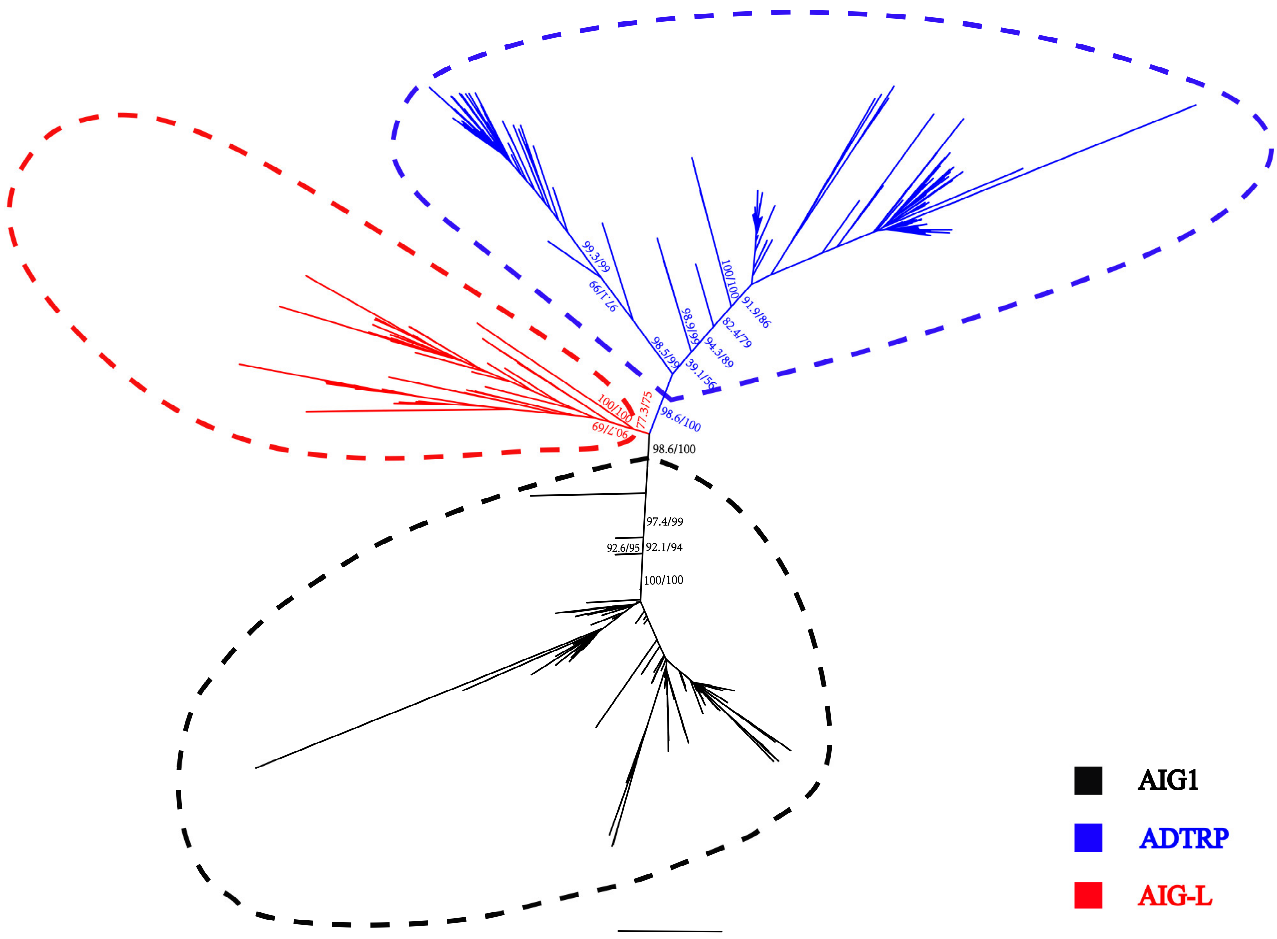

Figure S5. Phylogenetic representation of AIG protein sequences in all available vertebrates using the ML approach. The radial tree generated was supported by high aLRT (numerators) and bootstrap values (denominators). The values for ADTRP, AIG1, and AIG-L are 98.6/100, 98.6/100, and 77.3/75, respectively.

Figure S6–S9. Phylogenetic trees generated by ML approach for mammals (S6), birds (S7), reptiles (S8), and amphibians (S9). ADTRP, AIG1, and AIG-L are labeled by blue, black, and red, respectively. The AIG-L from *Ciona intestinalis* was used as the outgroup.

Figure S6

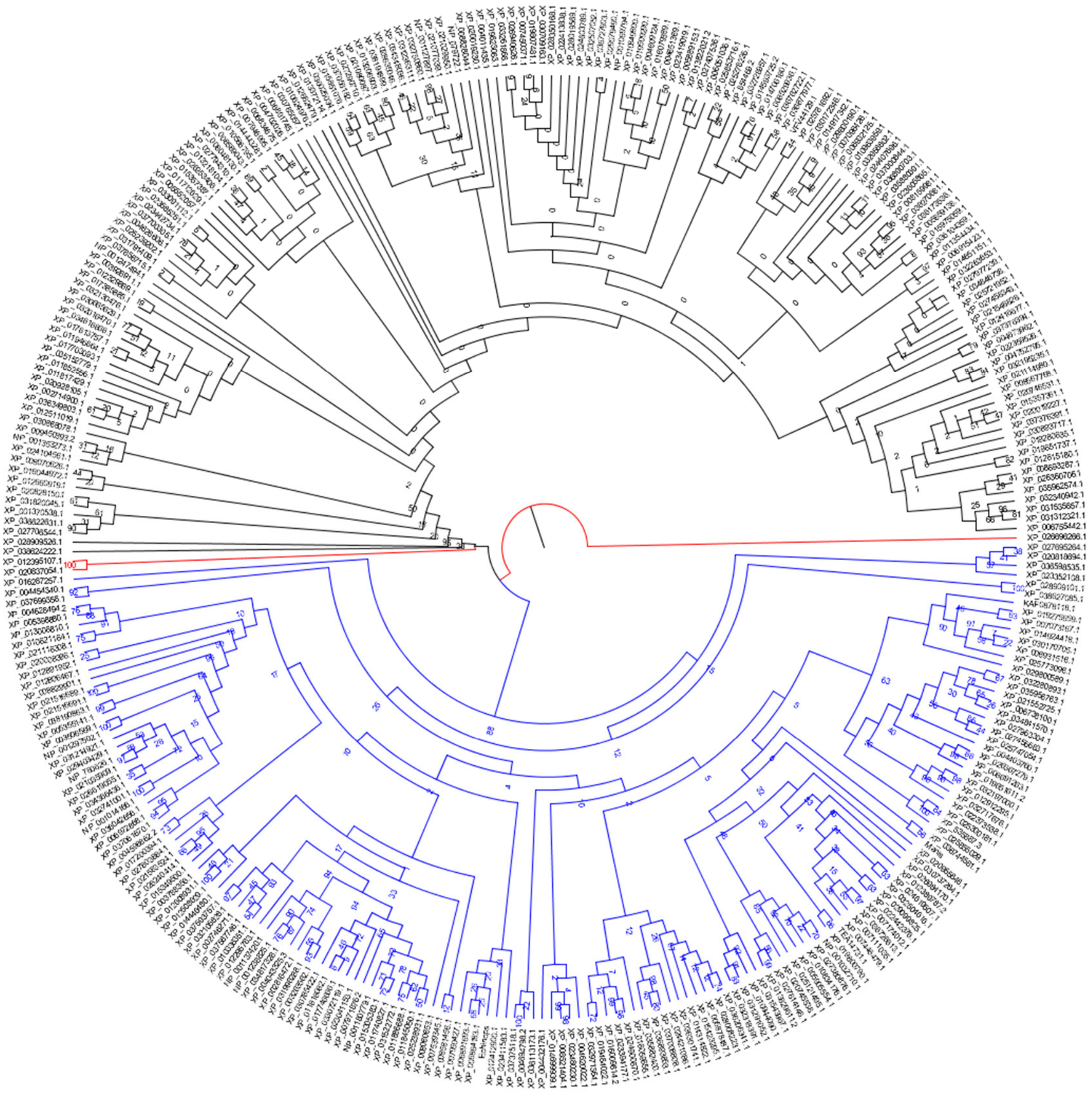

Figure S7

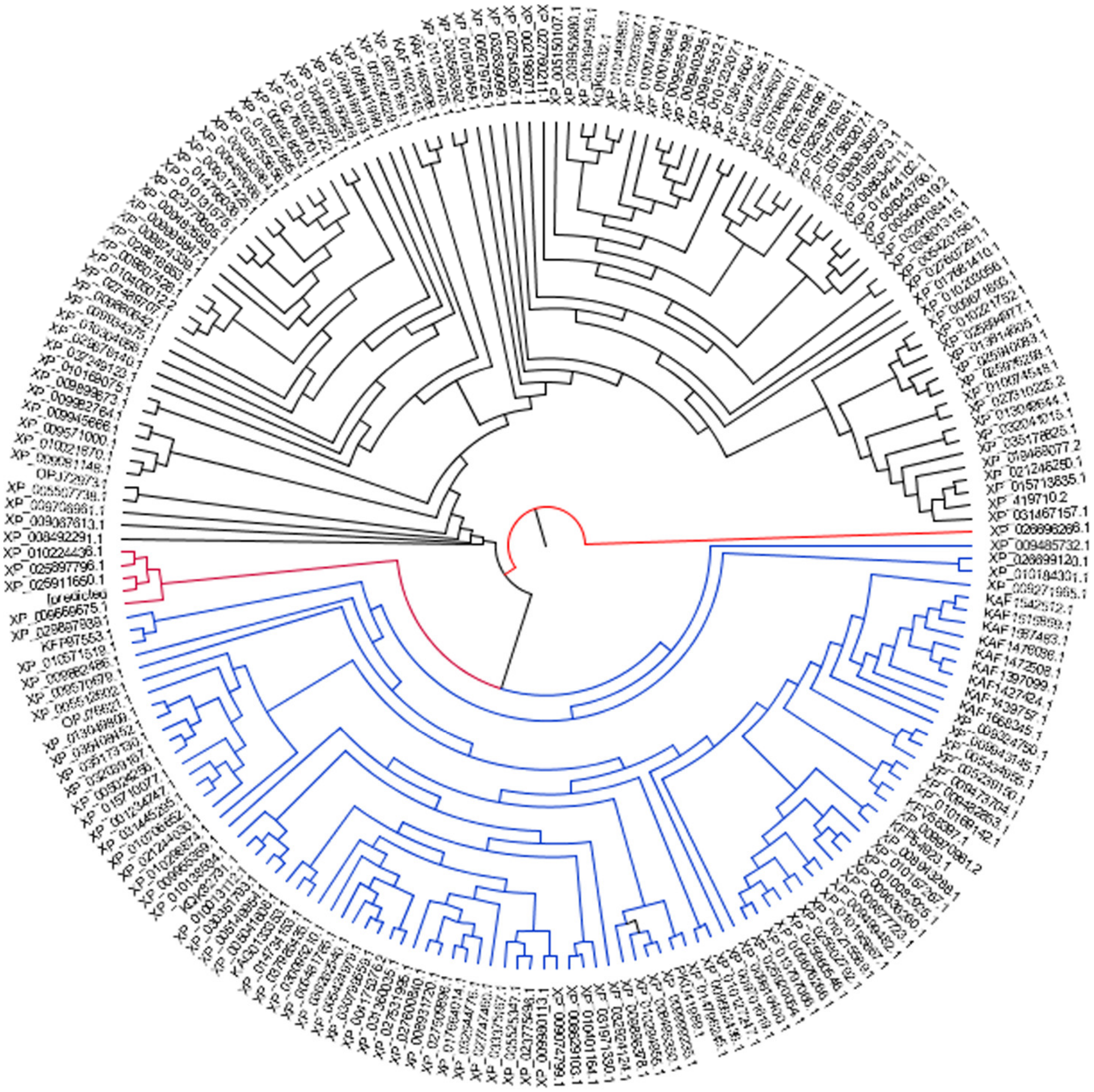

Figure S8

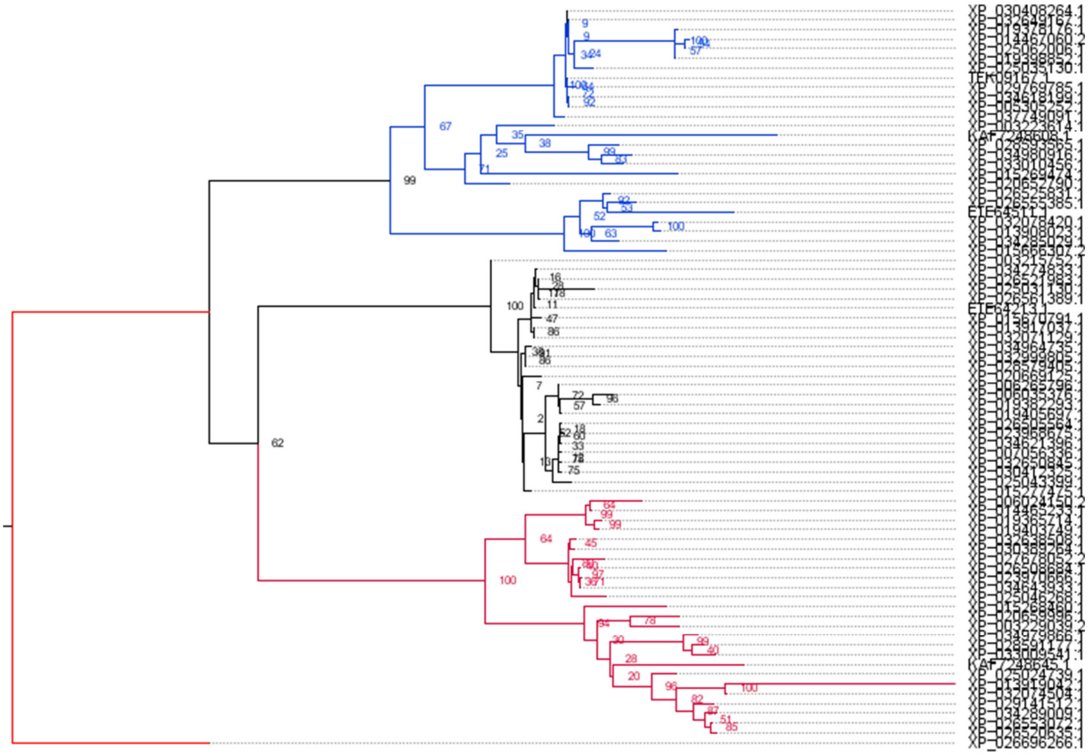

Figure S9

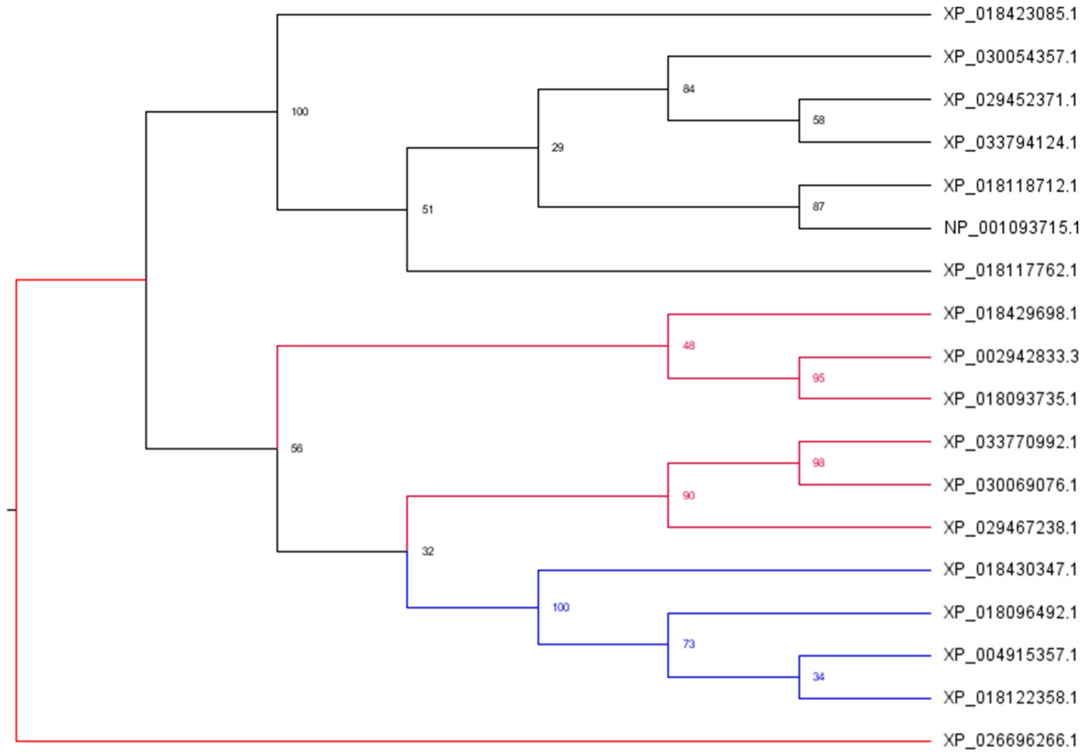
XP_018423085.1
XP_030054357.1
XP_029452371.1
XP_033794124.1
XP_018118712.1
NP_001093715.1
XP_018117762.1
XP_018429698.1
XP_002942833.3
XP_018093735.1
XP_033770992.1
XP_030069076.1
XP_029467238.1
XP_018430347.1
XP_018096492.1
XP_004915357.1
XP_018122358.1
XP_026696266.1

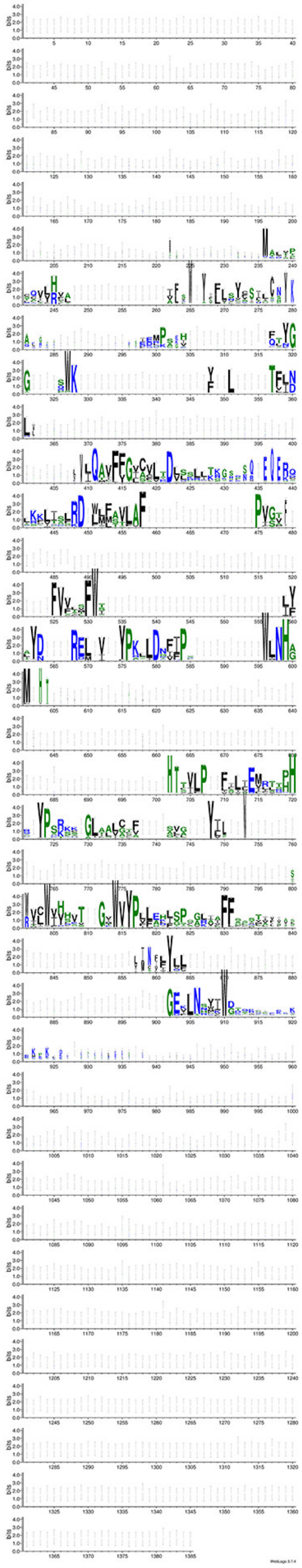

Figure S10. Graphical representation of the conserved amino acid sites across all available AIG protein sequences.
