# Supplementary material for: Molecular Phylogenetic Analysis of the AIG Family in Vertebrates
Source: Genes (Basel). 2021 Jul 30;12(8):1190. doi: 10.3390/genes12081190 (PMC8394805; doi:10.3390/genes12081190)
Supplement: Supplementary file 1 [file genes-12-01190-s001.zip › genes-1297795-Supplementary-Materials-Final/Supplementa Data 2.pdf]

AIG1

Fish

Lampetra japonicum

Petromyzon marinus XP_032808881.1、XP_032808882.1

-<-laminin subunit alpha-5-L-<--<-stathmin-2-L-<-->-RTEL1->-->-glutathione S-transferase A4-L->--<-MAP kinase-interacting serine/threonine-protein kinase 1-L-<-->-ERMP1->-->-RIOK1->-->-bone morphogenetic protein 7-L->--<-GCLC-<--<-PHACTR1-L-<--<-EDN-2-L-<--<-ZFP40-L-<-**-<-AIG1-<-->-AIG1->-**-<-SGK3-L-<-->-SNPH-L->-->-VPS28->-->-PTP4A1-L->-->-ADGRB3-L->-->-COL22A1->-->-elongation of very long chain fatty acids protein 5-L->--<-synaptonemal complex protein 2-L-<-

Chondrichthyes

Summary：

-<-REPS1-<-->-ABRACL->-->-HECA->--<-TXLNB-<--<-CITED2-<--<-NMBR-<-->-GJE1->-->-VTA1->-->-ADGRG6->--<-EDN-2-<--<-HIVEP2-<-->-**AIG1**->--<-ADAT2-<-->-PEX3->-->-CD109->--<-COL12A1-<-->-MYCT1->-->-VIP-L->-->-VIP-L->-->-VIP-L->--<-FBXO5-<--<-MTRF1L-<-

Amblyraja radiata XP_032881931.1

-<-REPS1-<-->-ABRACL->-->-HECA->--<-TXLNB-<--<-CITED2-<--<-NMBR-<-->-GJE1->-->-VTA1->-->-ADGRG6->--<-EDN-2-L-<--<-HIVEP2-<-->-**AIG1**->--<-ADAT2-<-->-PEX3->-->-CD109->--<-COL12A1-<-->-MYCT1->-->-VIP-L->-->-VIP-L->-->-VIP-L->--<-FBXO5-<--<-MTRF1L-<-

Callorhinchus milii XP_007904525.1

->-MTRF1L->-->-FBXO5->--<-VIP-<--<-MYCT1-<-->-COL12A1->-//-<-PEX3-<-->-ADAT2->--<-HIVEP2-<-->-**AIG1**->--<-GPR126-<--<-VTA1-<-->-NMBR->-

spotted gar XP_015194603.1

-<-NMBR-<-->-GJE1/CX23-L->-->-VTA1->-->-ADGRG6->--<-EDN1-L-<--<-HIVEP2-<-->-**AIG1**->--<-ADAT2-<-->-PEX3->--<-FUCA2-<-->-ATF3->--<-BATF3-<--<-NSL1-<-->-TATDN3->--<-myosin regulatory light chain 2, skeletal muscle isoform-L-<-->-FLVCR1->-->-VASH2->-

Polypteriformes XP_028667660.1

->-ANGEL2->--**<-VASH2-<--<-FLVCR1-<--<-TATDN3-<--<-TATDN3-<-->-NSL1->-->-BATF3->--<-ATF3-<-**//-<-REPS1-<-->-ABRACL->-->-HECA->--<-TXLNB-<--<-CITED2-<--**<-NMBR-<-->-GJE1->-->-VTA1->--**>-AIG1->--**<-ADAT2-<-->-PEX3->--<-FUCA2-<--**>-IGF2R->-->- solute carrier family 22 member 2-L->--<-CEP170-<-->-SDCCAG8->--<-AKT3-<-->-ZBTB18->-->-ADSS->--<-TTBK1-<--<-two pore calcium channel protein 1-L-<-->-NPHP1->-->-HAAO->-//-

Salmoniformes

Salvelinus alpinus XP_023829517.1

-<-SCARA3-<-->-CLU->--<-REPS1-<-->-HECA->--<-TXLNB-L-<--<-CITED2-<--<-NMBR-L-<-->-GJE1->-->-VTA1->-->-ADGRG6->--<-EDN-1-L-<--<-HIVEP2-<-->-AIG1->--<-ADAT2-<-->-PEX3->-->-ATF3->--<-BATF3-<--<-NSL1-<-->-TATDN3->-->-FLVCR1->-->-VASH2->--<-ANGEL2-<--<-GALNT14-L-<-->-EHD3-L->-->-STUM->--<-ITPKB-L-<-

Salvelinus alpinus XP_023990972.1

->-ADGRG6-L->--<-EDN-1-L-<-->-**AIG1**->-->-PEX3-L->-->-ATF3-L->--<-BATF3-<-->-FLVCR1-L->-->-VASH2-L->--<-GALNT14-L-<-->-EHD3->-->--STUM->-

Salmo trutta XP_029607070.1

->-CLU-L->--<-REPS1-<-->-ABRACL->-->-HECA-L->--<-TXLNB-L-<--<-CITED2-<--<-NMBR-L-<-->-GJE1-L->-->-VTA1-L->-->-ADGRG6-L->--<-EDN-1-L-<--<-HIVEP2-<-->-AIG1->-->-PEX3-L->-->-ATF3-L->--<-BATF3-<-->-FLVCR1-L->-->-tubulinyl-Tyr carboxypeptidase2-L->--<-ANGEL2-L-<--<-GALNT14-L-<-->-EHD3-L->-->--STUM->-

Salmo trutta XP_029589533.1

-<-SCARA3-L-<-->-CLU-L->--<-REPS1-L-<-->-HECA->--<-TXLNB-L-<--<-CITED2-L-<--<-NMBR-L-<-->-GJE1->-->-VTA1-L->-->-ADGRG6-L->--<-EDN-1-L-<--<-HIVEP2-L-<-->-AIG1->--<-ADAT2-<-->-PEX3->-->-ATF3->--<-BATF3-<--<-NSL1-<-->-TATDN3->-->-FLVCR1->-->-VASH2->--<-ANGEL2-<--<-GALNT14-L-<-->-EHD3-L->-->-STUM->-

Salmo salar XP_014060691.1

->-CLU-L->--<-REPS1-L-<-->-ABRACL->-->-HECA->--<-TXLNB-L-<--<-CITED2-<--<-NMBR-L-<-->-GJE1-L->-->-VTA1-L->-->-ADGRG6-L->--<-EDN-1-L-<--<-HIVEP2-<-->-**AIG1**->-->-PEX3->-->-ATF3-L->--<-BATF3-<-->-FLVCR1-L->-->-VASH2-L->--<-GALNT14-L-<-->-EHD3->--<-FAM127C-L-<-->-STUM->-

Salmo salar XP_013999977.1

-<-TXLNB-L-<--<-CITED2-L-<--<-NMBR-L-<-->-GJE1-L->-->-VTA1-L->-->-ADGRG6-L->--<-EDN-1-L-<-->-pollen-specific leucine-rich repeat extensin-L->--<-HIVEP2-L-<-->-AIG1->--<-ADAT2-<-->-PEX3->-->-ATF3->--<-BATF3-<--<-NSL1-<-->-TATDN3->-->-FLVCR1->-->-VASH2->--<-ANGEL2-<--<-GALNT14-L-<-->-EHD3-L->-->-STUM->-

Oncorhynchus tshawytscha XP_024275910.1

-<-SCARA3-L-<-->-CLU-L->--<-REPS1-L-<-->-HECA->--<-TXLNB-<--<-CITED2-L-<--<-NMBR-L-<-->-GJE1-L->-->-VTA1->-->-ADGRG6->--<-EDN-1-L-<--<-HIVEP2-<-->-AIG1->--<-ADAT2-<-->-PEX3->-->-ATF3->--<-BATF3-<--<-NSL1-<-->-TATDN3->-->-FLVCR1->-->-VASH2-L->--<-ANGEL2-<--<-GALNT14-<-

Oncorhynchus tshawytscha XP_024234448.1

-<-ITPKB-L-<--<-ITPKB-L-<-->-EHD1R-L->-->-EHD3R-L->--<-GALNT14-L-<--<-GALNT14-L-<-->-VASH2-L->-->-FLVCR1-L->-->-ATF3-L->--<-BATF3-<-->-AIG1->--<-HIVEP2-L-<-->-ADGRG6-L->-->-ADGRG6-L->-->-integumentary mucin A.1-L->-->-ADGRG6-L->--<-VTA1-<-->-NMBR-L->--<-CITED2R-L-<--<-TXLNB-<--<-SCARA3-<-

Oncorhynchus nerka XP_029532576.1

->-ABRACL-L->-->-HECA-L->--<-TXLNB-L-<--<-CITED2-L-<--<-NMBR-L-<-->-GJE1-L->-->-VTA1->-->-ADGRG6-L->--<-EDN-1-<--<-HIVEP2-L-<-->-AIG1->-->-ATF3->--<-BATF3-<-->-FLVCR1->-->-tubulinyl-Tyr carboxypeptidase 2-L->--<-GALNT14-<-->-EHD3->-->-STUM->-

Oncorhynchus kisutch XP_020352141.1

-<-SCARA3-L-<-->-CLU-L->--<-REPS1-L-<-->-ABRACL-L->-->-HECA->--<-TXLNB-<--<-CITED2-<--<-NMBR-<-->-GJE1->-->-VTA1->-->-ADGRG6->--<-HIVEP2-<-->-AIG1->--<-ADAT2-<-->-PEX3->-->-ATF3->--<-BATF3-<--<-NSL1-<-->-TATDN3->-->-FLVCR1->-->-VASH2->--<-ANGEL2-<--<-GALNT14-L-<-->-EHD3-L->-->-STUM->-

Oncorhynchus kisutch XP_020310436.1

-<-REPS1-L-<-->-ABRACL->-->-HECA->--<-TXLNB-<--<-CITED2-L-<--<-NMBR-<-->-GJE1->-->-VTA1->-->-ADGRG6->--<-EDN-1-L-<--<-HIVEP2-<-->-AIG1->-->-ATF3->--<-BATF3-<-->-FLVCR1->-->-tubulinyl-Tyr carboxypeptidase 2-L->--<-GALNT14-L-<-->-EHD3-L->-->-STUM->-

Oncorhynchus keta XP_035650065.1

-<-SCARA3-L-<-->-CLU-L->--<-REPS1-L-<-->-ABRACL-L->-->-HECA->--<-TXLNB-<--<-CITED2-<--<-NMBR-<-->-GJE1->-->-VTA1->-->-ADGRG6->--<-EDN-

1-<--<-HIVEP2-<-->-AIG1->--<-ADAT2-<-->-PEX3->-->-ATF3->--<-BATF3-<--<-NSL1-<-->-TATDN3->-->-FLVCR1->-->-VASH2->--<-ANGEL2-<--<-GALNT14-<-->-EHD3->-

Oncorhynchus keta XP_035630814.1

-<-REPS1-<-->-ABRACL->-->-HECA->--<-TXLNB-L-<--<-CITED2-L-<--<-NMBR-L-<-->-GJE1-L->-->-VTA1->-->-ADGRG6-L->--<-EDN-1-L-<--<-HIVEP2-L-<-->-AIG1->-->-ATF3-L->--<-BATF3-<-->-FLVCR1-L->-->-tubulinyl-Tyr carboxypeptidase 2-L->--<-GALNT14-L-<-->-EHD3-L->-->--STUM>-

Oncorhynchus mykiss(rainbow trout) XP_021456153.1

-<-SCARA3-<-->-CLU->--<-REPS1-<-->-HECA->--<-TXLNB-<--<-CITED2-<--<-NMBR-<-->-GJE1->-->-VTA1->-->-ADGRG6->--<-EDN-1-L-<--<-HIVEP2-<-->-AIG1->-->-ATF3->--<-BATF3-<-->-FLVCR1->-->- tubulinyl-Tyr carboxypeptidase 2->--<-GALNT14-<-->-EHD3->-->-STUM->--<-ITPKB-<-

Oncorhynchus mykiss(rainbow trout) XP_021467669.2

-<-SCARA3-<-->-CLU->--<-REPS1-<-->-ABRACL-L->-->-HECA->--<-TXLNB-<--<-CITED2-<--<-NMBR-<-->-GJE1->-->-VTA1->-->-ADGRG6->--<-EDN-1-<--<-HIVEP2-<-->-AIG1->--<-ADAT2-<-->-PEX3->-->-ATF3->--<-BATF3-<--<-NSL1-<-->-TATDN3->-->-FLVCR1->-->-VASH2->--<-ANGEL2-<--<-GALNT14-<-->-EHD3->-->-STUM->--<-ITPKB-<-

Cyprinina

Sinocyclocheilus rhinocerous XP_016378447.1

-<-NMBR-L-<-->-GJE1-L->-->-GJE1-L->-->-VTA1->-->-ADGRG6-L->--<-EDN-1-L-<--<-HIVEP2-L-<-->-AIG1->--<-ADAT2-L-<-->-PEX3-L->-->-ATF3->--<-BATF3-<--<-NSL1-<-->-TATDN3->-->-FLVCR1->-->-VASH2->--<-ANGEL2-<-->-GALNT14-L->-->-EHD3-L->-

Sinocyclocheilus rhinocerous XP_016392951.1

->-ABRACL-L->-->-HECA->--<-TXLNB-L-<--<-CITED2-L-<--<-NMBR-L-<-->-GJE1-L->-->-VTA1-L->-->-VTA1-L->-->-ADGRG6-L->--<-EDN-2-L-<--<-HIVEP2-<-->-AIG1->--<-ADAT2-<-->-PEX3-L->-->-ATF3-L->--<-BATF3-<-->-TATDN3->--<-spermatogenesis-associated protein 45-L-<-->-FLVCR1-L->-->-FLVCR1-L->-->-VASH2-L->--<-ANGEL2-L-<-->-GALNT14-L->-

Sinocyclocheilus anshuiensis XP_016324805.1

->-ABRACL->-->-HECA->--<-TXLNB-L-<--<-CITED2-L-<--<-NMBR-L-<-->-GJE1-L->-->-VTA1->-->-ADGRG6-L->--<-EDN-1-L-<--<-HIVEP2-L-<-->-AIG1->--<-ADAT2-L-<-->-PEX3-L->-->-ATF3-L->--<-BATF3-<--<-NSL1-<-->-FLVCR1-L->-->-VASH2-L->--<-ANGEL2-L-<-->-GALNT14-L->-

Sinocyclocheilus anshuiensis XP_016342905.1

-<-PTRHD1-<--<-mitochondrial uncoupling protein 4-L-<--<-REPS1-L-<-->-ABRACL-L->-->-HECA->--<-TXLNB-L-<--<-CITED2-L-<--<-NMBR-L-<-->-GJE1-L->-->-VTA1->-->-ADGRG6-L->--<-EDN-2-L-<--<-HIVEP2-L-<-->-AIG1->--<-ADAT2-<-->-PEX3-L->-

Carassius auratus(goldfish) XP_026146528.1

-<-PTRHD1-<-->-ABRACL-L->-->-HECA->--<-TXLNB-L-<--<-CITED2-<-->-P2X purinoceptor 7-L->--<-NMBR-<-->-GJE1->-->-VTA1->-->-ADGRG6->--<-EDN-1-L-<--<-HIVEP2-L-<-->-AIG1->--<-ADAT2-<-->-PEX3->-->-ATF3->--<-BATF3-<--<-NSL1-<-->-TATDN3->--<-spermatogenesis-associated protein 45-L-<-->-FLVCR1-L->-->-VASH2-L->--<-ANGEL2-L-<-->-EHD3-L->-

Carassius auratus(goldfish) XP_026070453.1

-<-NMBR-L-<-->-GJE1-L->-->-VTA1-L->-->-ADGRG6-L->--<-EDN-1-L-<--<-HIVEP2-<-->-AIG1->-->-PEX3-L->-->-ATF3-L->--<-BATF3-<--<-NSL1-L-<-->-FLVCR1-L->-->-VASH2-L->--<-ANGEL2-L-<-->-GALNT14-L->-->-EHD3-L->-->-C-C motif chemokine 3-L->-->-STUM->-

Cyprinus carpio(common carp) XP_018976388.1

-<-digestive cysteine proteinase 1-L-<--<-pre-mRNA-splicing factor SPF27-<--<-DENN domain-containing protein 2C-L-<--<-fibronectin type III domain-containing protein 7-L-<-->-STXBP-L3->-->-RABGGTA-L->-->-POGZA-L->--<-PSMB4-L-<-->-STXBP3->-->-**AIG1**->-->-palmitoyltransferase ZDHHC18-L->-->-ARID1AB-L->-->-ARHGAP5-L->--<-abraxas2-<-->-eef1akmt2->--<-NHSL1A-<-->- intersectin-2-L->--<-LPIN1-L-<--<-GREB1-L-<--<-XK-related protein 6-L-<-

Cyprinus carpio(common carp) XP_018981242.1

->-sodium/potassium/calcium exchanger 4-L->--<-TMEM121AB-L-<-->-complement C1q tumor necrosis factor-related protein 6-L->-->-CBLN7-L->-->-HMBOX1-L->--<-exostosin-L 3-<-->-RASAL1-L->--<-affinity immunoglobulin gamma Fc receptor I-L-<--<-GGH-L-<--<-HIVEP2-L<-->-**AIG1**->--<-ADAT2-L-<-->-PEX3-L->-->-ATF3-L->--<-BATF3-<-->-trifunctional enzyme subunit beta, mitochondrial-L->--<-SYK-L-<--<-PR domain zinc finger protein 1-L-<-->-GAREML-L->--<-HADHAA-<-->-HADHB->-

Cyprinus carpio(common carp) XP_018926636.1

->-NKL-L->--<-TONSL-L protein-<-->-AIG1->--<-ADAT2-L-<-->-PEX3-L->-->-ATF3-L->--<-BATF3-<--<-NSL1-<-->-TATDN3->-->-FLVCR1-L->-->-VASH2-L->--<-ANGEL2-L-<-->-GALNT14-L->-

Sinocyclocheilus grahami XP_016091109.1 、XP_016091091.1

->-ABRACL->-->-HECA->--<-TXLNB-L-<--<-CITED2-L-<--<-NMBR-L-<-->-GJE1-L->-->-VTA1-L->-->-VTA1-L->-->-ADGRG6-L->--<-EDN-1-L-<--<-HIVEP2-<-->-

AIG1->-->-AIG1->--<-ADAT2-<-->-PEX3-L->-->-ATF3-L->--<-BATF3-<--<-NSL1-<-->-TATDN3->--<-spermatogenesis-associated protein 45-L-<-->-FLVCR1->-->-VASH2-L->--<-ANGEL2-L-<-->-GALNT14->-

Sinocyclocheilus grahami XP_016098123.1

->-ABRACL-L->-->-HECA->--<-TXLNB-L-<--<-CITED2-L-<--<-fibroin heavy chain-L-<--<-NMBR-L-<-->-GJE1-L->-->-VTA1-L->-->-ADGRG6-L->--<-EDN-2-L-<--<-HIVEP2-<-->-AIG1->--<-ADAT2-L-<-->-PEX3-L->-->-ATF3->--<-BATF3-<--<-NSL1-<-->-TATDN3->-->-FLVCR1-L->-->-VASH2->--<-ANGEL2-L-<-

Acipenseriformes XP_033873068.1

-<-REPS1-L-<-->-ABRACL->-->-HECA->--<-TXLNB-L-<--<-CITED2-L-<--<-NMBR-<-->-GJE1->-->-VTA1->-->-ADGRG6->--<-HIVEP2-L-<-->-AIG1->--<-ADAT2-<-->-PEX3->**--<-FUCA2-<--**<-zinc finger protein OZF-L-<-->-ectonucleotide pyrophosphatase/phosphodiesterase family member 3-L->-->-synaptotagmin-L protein 3->--<-ezrin-L-<--<-T-cell activation Rho GTPase-activating protein-L-<-->-fibronectin type III domain-containing protein 1-L->--<-SOD2-<-

Kurtiformes XP_029986389.1

-<-HIVEP2-<-->-AIG1->--<-ADAT2-<-->-PEX3->-->-ATF3->--<-BATF3-<--<-NSL1-<-->-TATDN3->-->-FLVCR1->-

Esociformes XP_010880894.1

->-CCDC25->--<-SCARA3-<-->-CLU->--<-REPS1-<-->-ABRACL->-->-HECA->--<-TXLNBA-<--<-CITED2-<--<-NMBR-<-->-GJE1->-->-VTA1->-->-ADGRG6->--<-HIVEP2-<-->-AIG1->--<-ADAT2-<-->-PEX3->-->-ATF3->--<-BATF3-<--<-NSL1-<-->-FLVCR1->-->-VASH2->--<-ANGEL2-<--<-GALNT14-<-

Osteoglossiformes

Summary：

-<-NMBR-<-->-GJE1->-->-VTA1->-->-ADGRG6->--<-EDN-1-L-<--<-HIVEP2-<-->-AIG1->--<-ADAT2-<-->-PEX3->-->-ATF3->--<-BATF3-<--<-NSL1-<--<-myosin regulatory light chain 2, skeletal muscle isoform-L-<-->-TATDN3->-->-FLVCR1->-->-VASH2->--<-ANGEL2-<--<-GALNT14-<-

Paramormyrops kingsleyae XP_023696715.1

-<-NMBR-<-->-GJE1->-->-VTA1->-->-ADGRG6->--<-EDN-1-L-<--<-HIVEP2-<-->-AIG1->--<-ADAT2-<-->-PEX3->-->-ATF3->--<-NSL1-<--<-myosin regulatory light chain 2, skeletal muscle isoform-L-<-->-TATDN3->-->-FLVCR1->-->-VASH2->--<-ANGEL2-<-**->-tigger transposable element-derived protein 1-L->-**-<-GALNT14-L-<-

Scleropages formosus XP_018597942.1

-<-NMBR-<-->-GJE1-L->-->-VTA1->-->-ADGRG6->--<-EDN-2-L-<--<-HIVEP2-<-->-AIG1->--<-ADAT2-<-->-PEX3->-->-ATF3->--<-BATF3-<--<-NSL1-<-**-<-myosin regulatory light chain 2, skeletal muscle isoform-L-<-**->-TATDN3->-->-FLVCR1->-->-VASH2->--<-ANGEL2-<--<-GALNT14-<-

Elopiformes XP_036406221.1

-<-SCARA3-<-->-CLU->--<-REPS1-<-->-ABRACL->-->-HECA->--<-TXLNBA-<--<-CITED2-<--<-NMBR-<-->-GJE1->-->-VTA1->-->-ADGRG6->--<-HIVEP2-<-->-AIG1->--<-ADAT2-<-->-PEX3->-->-ATF3->--<-BATF3-<--<-NSL1-<--<-myosin regulatory light chain 2, skeletal muscle isoform-L-<-->-TATDN3->--<-spermatogenesis-associated protein 45-L-<-->-FLVCR1->-->-VASH2->--<-ANGEL2-<--<-GALNT14-<-

Gonorhynchiformes XP_030627832.1

-<-FGFR1OP-<--<-MYT1LA-<--<-RNASEH1-<-->-RPS7->-->-ALLC->-->-DCDC2C->-//-<-SCARA3-<-->-CLU->--<-SLC25A27-<-->-ABRACL->-->-HECA->--<-TXLNB-<--<-CITED2-<--<-NMBR-<-->-GJE1->-->-VTA1->-->-ADGRG6->--<-HIVEP2-<-->-AIG1->--<-ADAT2-<-->-PEX3->-->-ATF3->--<-BATF3-<--<-NSL1-<-->-TATDN3->-->-FLVCR1->-->-VASH2->--<-ANGEL2-<-->-GALNT14-L->--

Danio rerio XP_009293152.1

->-ABRACL->-->-HECA->--<-TXLNB-<--<-CITED2-<--<-NMBR-<-->-GJE1->-->-VTA1->-->-ADGRG6->---si:ch211-202p1.5-<--<-HIVEP2-<---AIG1->--<-ADAT2-<-->-PEX3->-->-ATF3->--<-BATF3-<--<-NSL1-<-->-TATDN3->-->-FLVCR1->-->-VASH2->--<-ANGEL2-<-->-GALNT14->-->-EHD3->-

Anguilliformes XP_035278705.1

-<-SCARA3-<-->-CLU->--<-REPS1-<-->-ABRACL->-->-HECA->--<-TXLNBA-<--<-CITED2-<--<-NMBR-<-->-GJE1->-->-VTA1->-->-ADGRG6->---si:ch211-202p1.5-<--<-HIVEP2-<---AIG1->--<-ADAT2-<-->-PEX3->-->-ATF3->--<-BATF3-<--<-NSL1-<--

>-TATDN3->--<-myosin regulatory light chain 2, skeletal muscle isoform-L-<-->-FLVCR1->-->-VASH2->--<-ANGEL2-<--<-GALNT14-<-->-EHD3->-

Gobiiformes

Summary：

->-KCNK3->-->-SLC35F6->-->-PEX7->-->-SLC35D3->--<-TXLNB-<--<-NMBR-<-->-GJE1->-->-VTA1->-->-ADGRG6->-->-EDN-2-L->--<-HIVEP2-<-->-AIG1->--<-ADAT2-<-->-PEX3->--<-NSL1-<--<-NSL1-<-->-TATDN3->-->-FLVCR1->--<-FKBP6-<--<-TMEM244-<-->-TMEM200A->--<-EPB41L2-<-

Boleophthalmus pectinirostris XP_020774147.1

->-KCNK3->-->-SLC35F6->-->-PEX7->-->-SLC35D3->--<-TXLNB-<--<-NMBR-<-->-GJE1-L->-->-VTA1->-->-ADGRG6->-->-EDN-2-L->--<-HIVEP2-<-->-AIG1->--<-ADAT2-<-->-PEX3->--<-NSL1-<--<-NSL1-<-->-TATDN3->-->-FLVCR1-L->--<-FKBP6-<--<-TMEM244-<-->-TMEM200A->--<-EPB41L2-<-

Periophthalmus magnuspinnatus XP_033846739.1

->-KCNK3A->-->-SLC35F6->-->-PEX7->-->-SLC35D3->--<-TXLNB-<--<-NMBR-<-->-GJE1->-->-VTA1->-->-ADGRG6->-->-EDN-2-L->--<-HIVEP2-<-->-AIG1->--<-ADAT2-<-->-PEX3->--<-BATF3-<--<-NSL1-<-->-TATDN3->-->-FLVCR1->--<-FKBP6-<--<-TMEM244-<-->-TMEM200A->--<-EPB41L2-<-

Clupeiformes

Denticeps clupeoides XP_028858710.1、XP_028858227.1

-<-NMBR-<-->-GJE1->-->-VTA1->-->-ADGRG6->--<-EDN-3-L-<--<-HIVEP2-L-<-->-**AIG1**->--<-ADAT2-L-<-->-PEX3-L->-->-ATF3-L->--<-NMBR-L-<-->-GJE1-L->-->-VTA1->-->-ADGRG6-L->--<-EDN-3-L-<--<-HIVEP2-L-<**-->-AIG1->-**-<-ADAT2-L-<-->-PEX3-L->-->-ATF3->--<-BATF3-<--<-NSL1-<-<-spermatogenesis-associated protein 45-L-<-->-FLVCR1->-->-VASH2->--<-ANGEL2-<--<-GALNT14-L-<-

Clupea XP_012682776.1

->-VTA1->-->-ADGRG6->--<-HIVEP2-<-**->-ENAH->--<-LBR-<--<-CNIH3-<-->-CD2AP->--<-PTCHD4-<-->-slc5a6a->--<-transcription factor 23-L-<--<-EPHX1-<-<-SRP9-<-->-RPL7L1->-->-NCOA1->--<-METTL14-<--**>-AIG1->--<-ADAT2-<-->-PEX3->-->-ATF3->--<-NSL1-<--<-FLVCR1-<--<-ANGEL2-<-->-transcription factor EC-L->--<-FLVCR1-L-<--->-transcription factor EC-L->-

Holocentriforme XP_029903680.1

-<-HEY2-<-->-PEX7->-->-SLC36D3->--<-TXLNB-<--<-CITED2-<--<-NMBR-<-->-GJE1->-->-VTA1->-->-ADGRG6->-->-EDN-3-L->--<-HIVEP2-<-->-AIG1->--<-ADAT2-<-->-PEX3->-->-ATF3->--<-NSL1-<-->-TATDN3->--<-spermatogenesis-associated protein 45-L-<-->-FLVCR1-L->-//->-VASH2->--<-ANGEL2-<--<-GALNT14-<--<-ZFAND3-<--<-BTBD9-<-

Gadiformes XP_030200388.1

->-PSMC6->--<-VASH2-<-//-<-HEY2-<-->-PEX7->-->-SLC35D3->--<-TXLNB-<--<-NMBR-<-->-GJE1->-->-VTA1->-->-ADGRG6->-->-EDN-1-L->--<-HIVEP2-<-->-AIG1->--<-ADAT2-<-->-PEX3->-->-ATF3->--<-NSL1-<--<-spermatogenesis-associated protein 45-L-<-->-FLVCR1->--<-T-cell activation Rho GTPase-activating protein-L-<--<-opsin-5-L-<--<-PPP2R5A-<-//-<-NMBR-<-->-GJE1->-->-VTA1->-->-ADGRG6->-

Batrachoidiformes XP_034017752.1

-<-TMEM242-<-->-BUB1->-->-invertedformin-2-L->--<-NMBR-<-->-GJE1/CX23->-->-VTA1->-->-ADGRG6->-->-EDN-2->--<-HIVEP2-<-->-AIG1->--<-ADAT2-<-->-PEX3->-->-ATF3->--<-BATF3-<--<-NSL1-<-->-ASAP2B->-->-CPSF3->--<-ADAM17B-<-->-TAF1B->-//-<-RPS7-<-->-RNASEH1->-->-40S ribosomal protein S7-L->-->-ribonuclease H1-L->-->-MYT1LA->-//->-BTBD9->-->-ZFAND3->-->-GALNT14->-->-ANGEL2->-->-FLVCR1->-

Gymnotiformes XP_026875146.1

-<-SCARA3-<-->-ABRACL->-->-HECA->--<-TXLNBA-<--<-CITED2-<--<-NMBR-<-->-VTA1->-->-ADGRG6->--<-HIVEP2-<-->-AIG1->--<-ADAT2-<-->-PEX3->-->-zona pellucida sperm-binding protein 1-L->-->- zona pellucida sperm-binding protein 1-L->-->- zona pellucida sperm-binding protein 1-L->-->- zona pellucida sperm-binding protein 1-L->--<-proline-rich receptor-L protein kinase PERK8-<--<-zona pellucida sperm-binding protein 1-L-<--<-zona pellucida sperm-binding protein 1-L-<--<-zona pellucida sperm-binding protein 1-L-<--<-zona pellucida sperm-binding protein 1-L-<-->-ATF3->--<-BATF3-<--<-NSL1-<-->-FLVCR1->-->-VASH2->--<-ANGEL2-<--<-GALNT14-

Characiformes

Astyanax mexicanus XP_007245527.1

->-GALNT14->-->-ANGEL2->--<-VASH2-<--<-FLVCR1-<-->-spermatogenesis-associated protein 45->--<-TATDN3-<-->-NSL1->-->-BATF3->--<-mucin-17-L-<--<-ATF3-<--<-zona pellucida sperm-binding protein 3-L-<--<-HIVEP2-<-->-AIG1->--<-ADAT2-<-->-PEX3->-->-EDN-1-L->--<-ADGRG6-<--<-VTA1-<--<-GJE1-L-<-->-NMBR->-->-CITED2->-->-TXLNB->--<-HECA-<--<-ABRACL-<-->-SLC25A27->-

Colossoma macropomum XP_036438525.1

->-GALNT14->-->-ANGEL2->--<-VASH2-<--<-FLVCR1-<-->-spermatogenesis-associated protein 45-L->--<-TATDN3-<-->-NSL1->-->-BATF3->--<-ATF3-<--<-zona pellucida sperm-binding protein 3-L-<--<-zona pellucida sperm-binding protein 3-L-<--<-zona pellucida sperm-binding protein 3-L-<--<-zona pellucida sperm-binding protein 3-L-<--<-zona pellucida sperm-binding protein 3-L-<--<-HIVEP2-<-->-AIG1->--<-ADAT2-<-->-PEX3->-->-EDN-1->--<-ADGRG6-<--<-VTA1-<--<-GJE1-<-->-NMBR->-->-CITED2->-->-TXLNB->--<-HECA-<--<-ABRACL-<-->-SLC25A27->-

Pygocentrus nattereri XP_017556492.1

->-EPHX1->--<-zona pellucida sperm-binding protein 3-L-<--<-zona pellucida sperm-binding protein 3-L-<--<-zona pellucida sperm-binding protein 3-L-<--<-zona pellucida sperm-binding protein 3-L-<--<-zona pellucida sperm-binding protein 3-L-<--<-zona pellucida sperm-binding protein 3-L-<--<-zona pellucida sperm-binding protein 3-L-<--<-zona pellucida sperm-binding protein 3-L-<--<-zona pellucida sperm-binding protein 3-L-<--<-HIVEP2-<-->-AIG1->--<-ADAT2-<-->-PEX3->-->-EDN-1->--<-ADGRG6-<--<-VTA1-<--<-LOC111191365-<--<-GJE1-<-->-NMBR->-->-CITED2->-->-TXLNB->--<-HECA-<--<-ABRACL-<-->-SLC25A27->-//->-ATF3->--<-BATF3-<--<-NSL1-<-->-TATDN3->--<-spermatogenesis-associated protein 45-L-<-->-FLVCR1->-->-VASH2->--<-ANGEL2-<--<-GALNT14-<-

Carangaria incertae sedis XP_018538476.1

-<-HIVEP2-<-->-AIG1->--<-ADAT2-<-->-PEX3->-->-ATF3->--<-BATF3-<--<-NSL1-<-->-TATDN3->--<-spermatogenesis-associated protein 45-L-<-->-FLVCR1->-//->-VASH2->--<-ANGEL2-L-<--<-GALNT14-L-<--<-ZFAND3-L-<--<-BTBD9-<-

Eupercaria incertae sedis

Larimichthys crocea XP_019114706.1

->-CXIH1ORF198->-->-CLEC11A->-->-SELENOL->--<-TTC13-<-->-cnksr3->--<-RPS7-<-->-RNASEH1->-->-MYT1LA->-->-E2F6->--<-HIVEP2-<-->-AIG1->--<-ADAT2-<-->-PEX3->-->-ATF3->--<-BATF3-<--<-NSL1-<-->-TATDN3->--<-

spermatogenesis-associated protein 45-L-<-->-FLVCR1->-//->-VASH2->--<-ANGEL2-<--<-GALNT14-<--<-ZFAND3-<--<-BTBD9-<-

Morone saxatilis XP_035535140.1、XP_035535141.1

->-CEBPZOS-L->--<-CEBPZ-<-->-NDUFAF7->--<-PRKD3-L-<-->-CNKSR3->--<-RPS7-<-->-RNASEH1->-->-MYT1LA->-->-E2F6->-->-NACHT, LRR and PYD domains-containing protein 3-L->--<-HIVEP2-<-**->-AIG1->-->-AIG1->-**->-PEX3->-->-ATF3->--<-BATF3-<--<-NSL1-<-->-TATDN3->->-FLVCR1->-//->-VASH2->--<-ANGEL2-<--<-GALNT14-<--<-patatin-L phospholipase domain-containing protein 4-<--<-ZFAND3-L-<--<-BTBD9-<-//-<-ADGRG6-<--<-VTA1-<--<-GJE1-<-->-NMBR->-

Ovalentaria incertae sedis

Parambassis ranga XP_028252524.1

-<-CEBPZ-<-->-NDUFAF7->--<-PRKD3-<-->-CNKSR->--<-RPS7-<-->-RNASEH1->-->-MYT1LA->-->-E2F6->--<-HIVEP2-<--->-AIG1->--<-ADAT2-<-->-PEX3->-->-ATF3->--<-BATF3-<--<-NSL1-<-->-TATDN3->---<-spermatogenesis-associated protein 45-L-<-->-FLVCR1->-//->-VASH2->--<-ANGEL2-L-<--<-GALNT14-L-<--<-ZFAND3-<--<-BTBD9-<-

Amphiprion ocellaris XP_023123531.1

-<-VASH2-<-//-<-IPCEF1-<--<-RPS7-<-->-RNASEH1->-->-MYT1LA->--<-HIVEP2-<-->-AIG1->--<-ADAT2-<-->-PEX3->-->-ATF3->--<-BATF3-<--<-NSHaplochromis burtoniL1-<-->-TATDN3->--<-spermatogenesis-associated protein 45-L-<-->-FLVCR1->-

Cichliformes

Pundamilia nyererei XP_005732761.1

-<-ADGRG6-<--<-VTA1-<--<-GJE1-<-->-NMBR->-//-<-CEBPZ-<-->-NDUFAF7->--<-PRKD3-<-->-CNKSR3->-->-IPCEF1-L->--<-RPS7-<-->-RNASEH1->-->-MYT1LA->-->-E2F6->--<-HIVEP2-<-->-AIG1->--<-ADAT2-<-->-PEX3->-->-ATF3->--<-BATF3-<--<-NSL1-<-->-TATDN3->--<-spermatogenesis-associated protein 45-L-<-->-FLVCR1->-

Oreochromis niloticus XP_005452457.1

-<-ADGRG6-<--<-VTA1-<--<-GJE1-<-->-NMBR->-//-<-CEBPZ-<-->-NDUFAF7->--<-PRKD3-<-->-CNKSR3->--<-RPS7-<-->-RNASEH1->-->-MYT1LA->-->-E2F6->--<-HIVEP2-<-->-AIG1/PEX3->--<-ADAT2-<-->-ATF3->--<-BATF3-<--<-NSL1-<-->-

TATDN3->--<-spermatogenesis-associated protein 45-<-->-FLVCR1->-//->-VASH2->--<-ANGEL2-<--<-GALNT14-<--<-ZFAND3-<--<-BTBD9-<-

Neolamprologus brichardi XP_006793576.1

-<-ADGRG6-<--<-VTA1-<--<-GJE1-<-->-NMBR->-//-<-CEBPZ-<-->-NDUFAF7->--<-PRKD3-<-->-CNKSR3->--<-RPS7-<-->-RNASEH1->-->-MYT1LA->-->-E2F6->--<-HIVEP2-<-->-AIG1->--<-ADAT2-<-->-PEX3->-->-ATF3->--<-BATF3-<--<-NSL1-<-->-TATDN3->-->-FLVCR1->-//->-VASH2->--<-ANGEL2-<--<-GALNT14-<--<-ZFAND3-<--<-BTBD9-<-

Maylandia zebra XP_004568499.1

-<-ADGRG6-<--<-VTA1-<--<-GJE1-<-->-NMBR->-//-<-CEBPZ-<-->-NDUFAF7->--<-PRKD3-<-->-CNKSR3->--<-RPS7-<-->-RNASEH1->-->-MYT1LA->-->-E2F6->--<-HIVEP2-<-->-AIG1->--<-ADAT2-<-->-PEX3->-->-ATF3->--<-BATF3-<--<-NSL1-<-->-TATDN3->-->-FLVCR1->-//->-VASH2->--<-ANGEL2-<--<-GALNT14-<--<-ZFAND3-<--<-BTBD9-<-

Haplochromis burtoni XP_005929958.1

-<-CEBPZ-<-->-NDUFAF7->--<-PRKD3-<-->-CNKSR3->**-->-IPCEF1->--**<-RPS7-<-->-RNASEH1->-->-MYT1LA->-->-E2F6->--<-HIVEP2-<-->-AIG1->--<-ADAT2-<-->-PEX3->-->-ATF3->--<-BATF3-<--<-NSL1-<-->-TATDN3->-->-FLVCR1->-//->-VASH2->--<-ANGEL2-<--<-GALNT14-<--<-ZFAND3-L-<--<-BTBD9-<-

Archocentrus centrarchus XP_030577334.1

-<-ADGRG6-<--<-VTA1-<--<-GJE1-<-->-NMBR->-//-<-CEBPZ-<-->-NDUFAF7->--<-PRKD3-<-->-CNKSR3->--<-RPS7-<-->-RNASEH1->-->-MYT1LA->-->-E2F6->**--<-pleckstrin homology domain-containing family F member 1-L-<-->-NLRC3-L->-->-NLRC3-L->--**<-HIVEP2-<-->-AIG1->--<-ADAT2-<-->-PEX3->-->-ATF3->--<-BATF3-<--<-NSL1-<-->-TATDN3->--<-spermatogenesis-associated protein 45-L-<-->-FLVCR1->-//->-VASH2->--<-ANGEL2-<--<-GALNT14-L-<--<-ZFAND3-L-<--<-BTBD9-<-

Astatotilapia calliptera XP_025998124.1

-<-ADGRG6-<--<-VTA1-<--<-GJE1-<-->-NMBR->-//-<-CEBPZ-<-->-NDUFAF7->--<-PRKD3-<-->-CNKSR3->--<-RPS7-<-->-RNASEH1->-->-MYT1L->-->-E2F6->**-->-FLO11-L->--**<-HIVEP2-<-->-AIG1->--<-ADAT2-<-->-PEX3->-->-ATF3->--<-BATF3-<--<-NSL1-<-->-TATDN3->--<-spermatogenesis-associated protein 45-L-<-->-FLVCR1->-//->-VASH2->--<-ANGEL2-<--<-GALNT14-L-<--<-ZFAND3-L-<--<-BTBD9-<-

Spariformes

Sparus aurata XP_030262001.1

-<-CEBPZ-<-->-NDUFAF7->--<-PRKD3-<-->-CNKSR3->-->-piggyBac transposable element-derived protein 3-L->--<-RPS7-<-->-RNASEH1->-->-MYT1LA->-->-E2F6->--<-HIVEP2-<-->-AIG1->--<-ADAT2-<-->-PEX3->-->-ATF3->--<-BATF3-<--<-NSL1-<-->-TATDN3->--<-spermatogenesis-associated protein 45-L-<-->-FLVCR1->-//->-VASH2->--<-ANGEL2-<--<-GALNT14-L-<--<-ZFAND3-<--<-BTBD9-<-//-<-NMBR-<--<-GJE1-<-->-VTA1->-->-ADGRG6->-

Acanthopagrus latus XP_036943018.1

-<-CEBPZ-<-->-NDUFAF7->--<-PRKD3-L-<-->-CNKSR3->-->-IPCEF1->--<-RPS7-<-->-RNASEH1->-->-MYT1LA->-->-E2F6->--<-HIVEP2-<-->-AIG1->--<-ADAT2-<-->-PEX3->-->-ATF3->--<-BATF3-<--<-NSL1-<-->-TATDN3->--<-spermatogenesis-associated protein 45-L-<-->-FLVCR1->-//->-VASH2->--<-ANGEL2-<--<-GALNT14-<--<-ZFAND3-<--<-BTBD9-<-//-<-NMBR-<--<-GJE1-<-->-VTA1->-->-ADGRG6->-

Pleuronectiformes

Scophthalmus maximus XP_035474152.1

-<-ADGRG6-<--<-VTA1-<--<-GJE1-<-->-NMBR->-//-<-CEBPZ-<-->-NDUFAF7->--<-PRKD3-L-<-->-CNKSR3->--<-RPS7-<-->-RNASEH1->-->-MYT1LA->-->-E2F6->--<-HIVEP2-<-->-AIG1->--<-ADAT2-<-->-PEX3->-->-ATF3->--<-BATF3-<--<-NSL1-<--<-spermatogenesis-associated protein 45-L-<-->-FLVCR1->-//->-VASH2->--<-ANGEL2-<--<-GALNT14-<--<-ZFAND3-L-<--<-BTBD9-<-

Hippoglossus stenolepis XP_034999777.1

-<-VASH2-<-//-<-ADGRG6-<--<-VTA1-<--<-GJE1-<-->-NMBR->-//-<-CEBPZ-<-->-NDUFAF7->--<-PRKD3-L-<-->-CNKSR3->--<-RPS7-<-->-RNASEH1->-->-MYT1LA->-->-E2F6->--<-HIVEP2-<-->-AIG1->--<-ADAT2-<-->-PEX3->-->-ATF3->--<-BATF3-<--<-NSL1-<-->-TATDN3->--<-spermatogenesis-associated protein 45-L-<-->-FLVCR1->-//-<-ANGEL2-<---<-GALNT14-<--<-ZFAND3-<--<-BTBD9-<-

Cynoglossus semilaevis XP_024912588.1

-<-VASH2-<-//-<-CEBPZ-<-->-NDUFAF7->--<-PRKD3-<-->-CNKSR3->-->-IPCEF1->--<-RPS7-<-->-RNASEH1->-->-MYT1LA->--<-HIVEP2-<-->-AIG1->--<-ADAT2-<-->-PEX3->-->-ATF3->--<-BATF3-<--<-NSL1-<-->-FLVCR1->-//-<-NMBR-<-->-VTA1->-->-ADGRG6->-

Hippoglossus hippoglossus XP_034436433.1

-<-ADGRG6-<--<-VTA1-<--<-GJE1-<-->-NMBR->-//-<-CEBPZ-<-->-NDUFAF7->--<-PRKD3-<-->-CNKSR3->--<-RPS7-<-->-RNASEH1->-->-MYT1LA->-->-E2F6->--<-HIVEP2-<-->-AIG1->--<-ADAT2-<-->-PEX3->-->-ATF3->--<-BATF3-<--<-NSL1-<--

>-TATDN3->--<-spermatogenesis-associated protein 45-L-<-->-FLVCR1->-//-<-VASH2-<--<-ANGEL2-<--<-GALNT14-<--<-ZFAND3-L-<--<-BTBD9-<-

Syngnathiformes

Hippocampus comes XP_019727029.1

-<-CEBPZ-<-->-NDUFAF7->--<-PRKD3-L-<--<-CNKSR3-<--<-RPS7-<--<-E2F6-<--<-MYT1LA-<--<-RNASEH1-<--<-HIVEP2-<-->-AIG1->--<-ADAT2-<-->-PEX3->-->-ATF3->--<-NSL1-<--<-spermatogenesis-associated protein 45-L-<-->-FLVCR1->-//-->-VASH2->--<-ANGEL2-<-->-phospholipid-metabolizing enzyme A-C1-L->--<-GALNT14-<--<-ZFAND3-L-<--<-BTBD9-<-

Syngnathus acus XP_037101098.1

-<-ARMC1L-<-->-MTFR2->--<-MYB-<-->-HBS1L->-->-ALDH8A1->-->-SGK1->-->-AIG1->--<-ADAT2-<-->-PEX3->-->-ATF3->--<-NSL1-<-->-FLVCR1->-//->-VASH2->--<-ANGEL2-<-->-phospholipase A and acyltransferase 2-L->--<-GALNT14-<--<-ZFAND3-<--<-BTBD9-<-//-<-NMBR-<-->-GJE1->-->-VTA1->-->-ADGRG6->-

Synbranchiformes

Summary：

-<-CEBPZ-<-->-NDUFAF7->--<-PRKD3-<-->-CNKSR3->--<-RPS7-<-->-RNASEH1->-->-MYT1LA->-->-E2F6->--<-HIVEP2-<-->-AIG1->--<-ADAT2-<-->-PEX3->-->-ATF3->--<-NSL1-<-->-TATDN3->--<-spermatogenesis-associated protein 45-L-<-->-FLVCR1->-//->-BTBD9->-->-ZFAND3->-->-GALNT14->-->-ANGEL2->--<-VASH2-<-

Mastacembelus armatus XP_026175198.1

-<-CEBPZ-<-->-NDUFAF7->--<-PRKD3-<-->-CNKSR3->--<-RPS7-<-->-RNASEH1->-->-MYT1LA->-->-E2F6->--<-HIVEP2-<-->-AIG1->--<-ADAT2-<-->-PEX3->-->-ATF3->--<-NSL1-<-->-TATDN3->--<-spermatogenesis-associated protein 45-L-<-->-FLVCR1->-//->-BTBD9->-->-ZFAND3-L->-->-GALNT14->-->-ANGEL2->--<-VASH2-<-

Monopterus albus XP_020442290.1

->-E2F6->--<-HIVEP2-<-->-AIG1->--<-ADAT2-<-->-PEX3->-->-ATF3->--<-BATF3-<--<-NSL1-<--<-spermatogenesis-associated protein 45-L-<-->-FLVCR1->-

Anabantiforms

Summary：

<-ADGRG6-<--<-VTA1-<--<-GJE1-<-->-NMBR->-//->-CD109->-->-LOC113149654->--<-COL12A1B-<-->-CNKSR3->--<-RPS7-<-->-RNASEH1->-->-MYT1LA->-->-E2F6->-->-NLRC3-L->--<-HIVEP2-<-->-AIG1->--<-ADAT2-<-->-PEX3->-->-ATF3->--<-BATF3-<--<-NSL1-<-->-TATDN3->--<-spermatogenesis-associated protein 45-L-<-->-FLVCR1->-//->-VASH2->--<-ANGEL2-<--<-GALNT14-<--<-ZFAND3-<--<-BTBD9-<-

Anabas testudineus XP_026197907.1

-<-ADGRG6-<--<-VTA1-<--<-GJE1-<-->-NMBR->-//-<-COL12A1B-<-->-CNKSR3-L->--<-RPS7-<-->-RNASEH1->-->-MYT1LA->-->-E2F6->-->-NLRC3-L->--<-HIVEP2-<-->-AIG1->--<-ADAT2-<-->-PEX3->-->-ATF3->--<-BATF3-<--<-NSL1-<-->-TATDN3->--<-spermatogenesis-associated protein 45-L-<-->-FLVCR1->-//->-VASH2->--<-ANGEL2-<--<-GALNT14-<--<-ZFAND3-<--<-BTBD9-<-

Betta splendens XP_028997379.1

-<-ADGRG6-<--<-VTA1-<--<-GJE1-<-->-NMBR->-//-<-COL12A1B-<-->-CNKSR3-L->-->-IPCEF1->--<-RPS7-<-->-RNASEH1->-->-MYT1L->-->-LOC114849205->--<-HIVEP2-<-->-AIG1->--<-ADAT2-<-->-PEX3->-->-ATF3->--<-BATF3-<-->-FLVCR1-L->--<-NSL1-<-->-FLVCR1-L->-//->-VASH2->--<-ANGEL2-L-<--<-GALNT14-L-<--<-ZFAND3-<--<-BTBD9-<-

Centrarchiformes XP_038582511.1

-<-ADGRG6-<--<-VTA1-<--<-GJE1-<-->-NMBR->-//-<-CEBPZ-<-->-NDUFAF7->--<-PRKD3-L-<-->-CNKSR3->--<-RPS7-<-->-RNASEH1->-->-MYT1LA->--<-piggyBac transposable element-derived protein 4-L-<--<-RNA polymerase-associated protein LEO1-L-<-->-E2F6->--<-HIVEP2-<-->-AIG1->--<-ADAT2-<-->-PEX3->-->-ATF3->--<-BATF3-<--<-NSL1-<-->-TATDN3->--<-spermatogenesis-associated protein 45-L-<-->-FLVCR1->-//->-VASH2->--<-ANGEL2-<--<-GALNT14-<--<-ZFAND3-<--<-BTBD9-<-

Carangiforms

Echeneis naucrates XP_029352737.1

-<-NMBR-<-->-GJE1->-->-VTA1->-->-NACHT, LRR and PYD domains-containing protein 3-L->-->-NACHT, LRR and PYD domains-containing protein 3-L->-->-TXLNB-L->--<-solute carrier family 35 member D3-L-<--<-peroxisomal targeting signal 2 receptor-L-<-->-ADGRG6->-//-<-CEBPZ-<-->-NDUFAF7->--<-PRKD3-<-->-CNKSR3->--<-RPS7-<-->-RNASEH1->-->-MYT1LA->--<-HIVEP2-<-->-AIG1->--<-ADAT2-<-->-PEX3->-->-ATF3->--<-BATF3-<--<-NSL1-<-->-TATDN3->--<-spermatogenesis-associated protein 45-L-<-->-FLVCR1->-//->-BTBD9->-->-ZFAND3-L->-->-GALNT14-L->-->-ANGEL2->--<-VASH2-<-

Seriola dumerili XP_022614659.1

->-CNKSR3-L->--<-RPS7-<-->-RNASEH1->-->-MYT1LA->->-E2F6->--<-HIVEP2-<-->-AIG1->--<-ADAT2-<-->-PEX3->-->-ATF3->--<-BATF3-<--<-NSL1-<-->-TATDN3->--<-spermatogenesis-associated protein 45-L-<-->-FLVCR1->-//->-VASH2->--<-ANGEL2-<--<-GALNT14-L-<--<-ZFAND3-<--<-BTBD9-<-

Seriola lalandi dorsalis XP_023253279.1

->-calmodulin-binding transcription activator 2-L->--<-H-2 class II histocompatibility antigen, I-A beta chain-L-<--<-HLA class II histocompatibility antigen, DRB1-3 chain-L-<--<-HLA class II histocompatibility antigen, DRB1-3 chain-L-<--<-PLOD3-<--<-HIVEP2-<-->-AIG1->--<-ADAT2-<-->-PEX3->-->-ATF3->--<-NSL1-<-->-TATDN3->--<-spermatogenesis-associated protein 45-L-<-->-FLVCR1->-

Tetraodontiformes

Takifugu rubripes(torafugu) XP_029705465.1

->-VASH2->--<-ANGEL2-<--<-GALNT14-<--<-ZFAND3-<--<-BTBD9-<-//-<-ADGRG6-<--<-VTA1-<-->-GJE1->-->-NMBR->-//-<-CEBPZ-<-->-NDUFAF7->--<-PRKD3-<--<-CNKSR3-<--<-RPS7-<-->-RNASEH1->-->-MYT1LA->-->-E2F6->--<-HIVEP2-<-->-AIG1->--<-ADAT2-<-->-PEX3->-->-ATF3-L->--<-NSL1-<-->-FLVCR1-L->-->-cytochrome P450 2K1-L->-->-ATF3->--<-NSL1-<-->-FLVCR1->-

Blenniiforms

Gouania willdenowi XP_028296322.1

->-PEX7->-->-SLC35D3->--<-TXLNB-<--<-FLVCR1-<-->-spermatogenesis-associated protein 45-L->-->-NSL1->--<-ATF3-<--<-PEX3-<-->-ADAT2->--<-HIVEP2-<-->-AIG1->--<--E2F6-<--<-MYT1LA-<--<-RNASEH1-<-->-RPS7->-->-NDUFAF7->--<-PRKD3-<-->-CNKSR3-L->-->-CEBPZ->--<-CEBPZOS-<-//-<-GALNT14-<--<-ZFAND3-<--<-BTBD9-<-

Salarias fasciatus XP_029966628.1

-<-ADGRG6-<--<-VTA1-<--<-GJE1-<-->-NMBR->-//-<-CEBPZ-<-->-NDUFAF7->--<-PRKD3-<--<-CNKSR3->--<-RPS7-<-->-RNASEH1->-->-MYT1LA->-->-E2F6->--<-HIVEP2-<-->-AIG1->--<-ADAT2-<-->-PEX3->-->-ATF3->--<-BATF3-<--<-NSL1-<-->-TATDN3->--<-spermatogenesis-associated protein 45-L-<-->-FLVCR1->-

Perciformes

Sebastes umbrosus XP_037607091.1

-<-ADGRG6-<--<-VTA1-<--<-GJE1-<-->-NMBR->-//-<-CEBPZ-<-->-NDUFAF7->--<-PRKD3-L-<-->-fatty acyl-CoA hydrolase precursor, medium chain-L->--<-CNKSR3-<--<-RPS7-<-->-RNASEH1->-->-MYT1LA->-->-E2F6->--<-HIVEP2-<-->-AIG1->--<-ADAT2-<-->-PEX3->-->-ATF3->--<-BATF3-<--<-NSL1-<-->-TATDN3->--<-spermatogenesis-associated protein 45-L-<-->-FLVCR1->-//->-BTBD9->-->-ZFAND3-L->-->-GALNT14->-->-ANGEL2->--<-VASH2-<-

Sander lucioperca XP_031163752.1

//-<-CNKSR3-<--<-RPS7-<-->-RNASEH1->-->-MYT1LA->-->-E2F6->--<-HIVEP2-<-->-AIG1->-->-cytochrome P450 2K1-L->-->-cytochrome P450 2K1-L->-->-cytochrome P450 2K1-L->-->-cytochrome P450 2K1-L->--<-ADAT2-<-->-PEX3->-->-ATF3->--<-BATF3-<--<-NSL1-<-->-TATDN3->--<-spermatogenesis-associated protein 45-L-<-->-FLVCR1->-//->-VASH2->--<-ANGEL2-<--<-GALNT14-<--<-ZFAND3-L-<--<-BTBD9-<-//-<-ADGRG6-<--<-VTA1-<--<-GJE1-<-->-NMBR->-

Pungitius pungitius XP_037305987.1

-<-NMBR-<-->-GJE1->-->-VTA1->-->-ADGRG6->-//-<-CFAP61-<-->-NDUFAF7->--<-PRKD3-<--<-IPCEF1-<--<-CNKSR3-<--<-RPS7-<-->-RNASEH1->-->-MYT1LA->-->-E2F6->--<-HIVEP2-<-->-AIG1->--<-ADAT2-<-->-PEX3->-->-ATF3->--<-NSL1-<-->-FLVCR1->-//->-VASH2->--<-ANGEL2-<--<-GALNT14-<--<-ZFAND3-L-<--<-BTBD9-<-

Perca flavescens XP_028461466.1

-<-ADGRG6-<--<-VTA1-<--<-GJE1-<-->-NMBR->-//-<-CEBPZ-<-->-NDUFAF7->--<-PRKD3-<--<-CNKSR3-<--<-RPS7-<-->-RNASEH1->-->-MYT1LA->-->-E2F6->--<-HIVEP2-<-->-AIG1->--<-ADAT2-<-->-PEX3->-->-ATF3->--<-BATF3-<--<-NSL1-<-->-TATDN3->-->-FLVCR1->-//->-VASH2->--<-ANGEL2-<--<-GALNT14-L-<--<-ZFAND3-<--<-BTBD9-<-

Etheostoma spectabile XP_032398681.1

-<-ADGRG6-<--<-VTA1-<--<-GJE1-<-->-NMBR->-//-<-CEBPZ-<-->-NDUFAF7->--<-PRKD3-<--<-IPCEF1-<--<-CNKSR3-<--<-RPS7-<-->-RNASEH1->-->-MYT1LA->-->-E2F6->--<-HIVEP2-<-->-AIG1->--<-ADAT2-<-->-PEX3->-->-ATF3->--<-BATF3-<--<-NSL1-<-->-TATDN3->--<-ANG-<--<-spermatogenesis-associated protein 45-L-<-->-FLVCR1->-//->-VASH2->--<-ANGEL2-L-<--<-GALNT14-<--<-ZFAND3-L-<--<-BTBD9-<-

Etheostoma cragini XP_034756660.1、XP_034756659.1

-<-ADGRG6-<--<-VTA1-<--<-GJE1-<-->-NMBR->-//-<-CEBPZ-<-->-NDUFAF7->--<-PRKD3-<--<--LIPCEF1-<--<-CNKSR3-L-<--<-RPS7-<-->-RNASEH1->-->-MYT1LA->-->-E2F6->--<-HIVEP2-<-->-AIG1->-->-AIG1->--<-ADAT2-<-->-PEX3->-->-ATF3-

>--<-BATF3-<--<-NSL1-<--<-ribonuclease-L-<-->-TATDN3->--<-spermatogenesis-associated protein 45-L-<-->-FLVCR1->-//->-VASH2->--<-LOC117961015-<--<-GALNT14-<--<-ZFAND3-L-<--<-BTBD9-<-

Cyclopterus lumpus XP_034384212.1

-<-ADGRG6-<--<-VTA1-<--<-GJE1-<-->-NMBR->-//->-NDUFAF7-L->--<-PRKD3-L-<-->-CNKSR3-L->--<-RPS7-<-->-RNASEH1->-->-MYT1LA->--<-procyclic form-specific polypeptide B1-alpha-L-<-->-E2F6->--<-HIVEP2-<-->-AIG1->--<-ADAT2-<-->-PEX3->-->-ATF3->--<-NSL1-<--<-spermatogenesis-associated protein 45-L-<-->-FLVCR1->-//->-VASH2->--<-ANGEL2-<--<-GALNT14-<--<-ZFAND3-L-<--<-BTBD9-<-

Epinephelus lanceolatus XP_033504063.1

-<-CEBPZ-<-->-NDUFAF7->--<-PRKD3-L-<-->-CNKSR3->--<-RPS7-<-->-RNASEH1->-->-MYT1LA->-->-E2F6->--<-HIVEP2-<-->-AIG1->--<-ADAT2-<-->-PEX3->-->-ATF3->--<-BATF3-<--<-NSL1-<-->-TATDN3->--<-spermatogenesis-associated protein 45-L-<-->-FLVCR1->-//->-VASH2->--<-ANGEL2-<--<-GALNT14-<--<-ZFAND3-L-<--<-BTBD9-<-//-<-ADGRG6-<--<-VTA1-<--<-GJE1-<-->-NMBR->-

Anarrhichthys ocellatus(wolf-eel) XP_031722072.1

-<-CEBPZ-<-->-NDUFAF7->--<-PRKD3-<--<-CNKSR3-<--<-RPS7-<-->-RNASEH1->-->-MYT1LA->-->-E2F6->-->-AIG1->--<-ADAT2-<-->-PEX3->-->-ATF3->--<-BATF3-<--<-NSL1-<-->-TATDN3->---<-spermatogenesis-associated protein 45-L-<-->-FLVCR1->--<-ANGEL2-<--<-GALNT14-L-<--<-ZFAND3-L-<--<-BTBD9-<-

Cottoperca gobio XP_029281681.1

->-BTBD9->-->-ZFAND3->-->-GALNT14-L->-->-ANGEL2->--<-VASH2-<-->-cytochrome P450 2K1-L->-->-cytochrome P450 2K1-L->--<-cytochrome P450 2K1-L-<--<-cytochrome P450 2K1-L-<-->-cytochrome P450 2K1-L->-->-cytochrome P450 2K1-L->--<-LOC115003546-<-->-cytochrome P450 2K3-L->-->-AIG1->-->-HIVEP2->--<--E2F6-<--<-MYT1LA-<--<-RNASEH1-<-->-RPS7->-->-CNKSR3->-->-interactor protein for cytohesin exchange factors 1-L->-->-PRKD3->--<-NDUFAF7-<-->-CEBPZ->--<-CEBPZOS-<-

Beloniformes

Oryzias latipes XP_023808847.1

->-general transcription factor II-I repeat domain-containing protein 2B-L->-->-FEZF1->-->-AIG1->--<-ADAT2-<-->-PEX3->-->-ATF3->-<-NSL1-<-->-FLVCR1->-->-ribonucleoside-diphosphate reductase subunit M2->--<-galectin-8-<--<-mRNA-ribonucleoside-diphosphate reductase subunit M2-L-<-->-TMEM242->-//->-HIVEP2->--<--E2F6-<--<-MYT1LA-<--<-RNASEH1-<-->-RPS7->-->-tripartite motif-containing

protein 16->--<-CNKSR3-<-->-PRKD3->--<-NDUFAF7-<-//-<-ADGRG6-<--<-VTA1-<--<-GJE1-<-->-NMBR->-

Oryzias melastigma XP_024146963.1

-<-RNASEH1-<-->-RPS7->-->-UFL1->--<-MYT1LA-<--<-CNKSR3-<-->-PRKD3->--<-NDUFAF7-<-//->-GNPAT->--<-VRTN-<--<-mitochondrial basic amino acids transporter-<-->-AIG1->--<-ADAT2-<-->-PEX3->-->-ATF3->--<-BATF3-<--<-NSL1-<-->-FLVCR1->--<-galectin-8-<--<-mRNA-ribonucleoside-diphosphate reductase subunit M2-L-<-->-TMEM242->-//-<-ADGRG6-<--<-VTA1-<--<-GJE1-<-->-NMBR->-

Cyprinodontiformes

Xiphophorus hellerii 、 Xiphophorus maculatus 、 Xiphophorus couchianus XP_027895452.1、XP_032440869.1、XP_005815438.1

->-BTBD9->-->-ZFAND3->-->-GALNT14-L->-->-ANGEL2->--<-VASH2-<-//-<-NMBR-<-->-GJE1->-->-VTA1->-->-ADGRG6->//->-TNFAIP2A-L->-->-MAP/microtubule affinity-regulating kinase 3-L->--<-creatine kinase B-type-<--<-photoreceptor outer segment membrane glycoprotein 2-L-<--<-NLRC12/3-L-<-->-CDC42BPB->--<-GPR132P-<--<-VIPAS39-<-->-AHSA1-L->--<-HIVEP2-<-->-AIG1->--<-ADAT2-<-->-PEX3->-->-ATF3->--<-BATF3-<--<-NSL1-<-->-TATDN3->-->-FLVCR1-L->-

Cyprinodon tularosa、Cyprinodon variegatus XP_038132908.1 、XP_015259359.1

->-AIG1->--<-ADAT2-<-->-PEX3->-->-ATF3->--<-BATF3-<--<-NSL1-<--<-spermatogenesis-associated protein 45-L-<-->-FLVCR1->-

Fundulus heteroclitus XP_012729814.1

->-BTBD9->-->-ZFAND3->-->-GALNT14->-->-ANGEL2->--<-VASH2-<-//->-TNFAIP2-L->-->-MAP/microtubule affinity-regulating kinase 3->--<-creatine kinase B-type-<--<-photoreceptor outer segment membrane glycoprotein 2-<--<-NLRC12-<-->-CDC42BPB->--<-GPR132P-<--<-VIPAS39-<-->-AHSA1->--<-HIVEP2-<-->-AIG1->--<-ADAT2-<-->-PEX3->-->-ATF3->--<-BATF3-<--<-NSL1-<-->-TATDN3->--<-spermatogenesis-associated protein 45-L-<-->-FLVCR1->-//-<-MYT1LA-<--<-RNASEH1-<-->-RPS7->--<-CNKSR2-<-->-NDUFAF7->--<-PRKD3-<-->-CNKSR3->-->-CEBPZ->--<-CEBPZOS-<-

Poecilia reticulata XP_008395278.1

->-trichohyalin-L->-->-MAP/microtubule affinity-regulating kinase 3-L->--<-creatine kinase B-type-<--<-photoreceptor outer segment membrane glycoprotein 2-L-<--<-NLRC3-L-<-->-CDC42BPB->--<-GPR132P-<--<-VIPAS39-<-->-AHSA1->-->-AIG1->--<-ADAT2-<-->-PEX3->-->-ATF3->--<-BATF3-<--<-NSL1-<-->-TATDN3->--<-

spermatogenesis-associated protein 45-L-<-->-FLVCR1->-//-<-NMBR-<-->-GJE1-L->--
>-VTA1->-->-ADGRG6->//->-BTBD9->-->-ZFAND3-L->-->-GALNT14->-->-
ANGEL2->--<-VASH2-<-

Poecilia formosa XP_016517610.1

->-TNFAIP2A-L->-->-MAP/microtubule affinity-regulating kinase 3-L->--<-creatine kinase B-type-<--<-photoreceptor outer segment membrane glycoprotein 2-L-<--<-NLRC12-L-<-->-CDC42BPB->--<-GPR132P-<--<-VIPAS39-<-->-AHSA1->--<-HIVEP2-<-->-AIG1->--<-ADAT2-<-->-PEX3->-->-ATF3->-->-BATF3->-->-TATDN3->--<-spermatogenesis-associated protein 45-L-<-->-FLVCR1->-

Poecilia latipinna XP_014902138.1

->-TNFAIP2A->-->-MAP/microtubule affinity-regulating kinase 3->--<-creatine kinase B-type-L-<--<-photoreceptor outer segment membrane glycoprotein 2-L-<--<-NLRC12-L-<-->-CDC42BPB->--<-GPR132P-<--<-VIPAS39-<-->-AHSA1->--<-HIVEP2-<-->-AIG1->--<-ADAT2-<-->-PEX3->--<-ribonuclease H1-L-<-->-RPS7->-->-ATF3->--<-BATF3-<--<-NSL1-<-->-TATDN3->--<-spermatogenesis-associated protein 45-L-<-->-FLVCR1-L->-

Poecilia mexicana XP_014836853.1

->-TNFAIP2A-L->-->-MAP/microtubule affinity-regulating kinase 3-L->--<-creatine kinase B-type-<--<-photoreceptor outer segment membrane glycoprotein 2-L-<--<-NLRC3-L-<-->-CDC42BPB-L->-->-CDC42BPB-L->--<-GPR132P-<--<-VIPAS39-<-->-AHSA1->--<-HIVEP2-<-->-AIG1->--<-ADAT2-<-->-PEX3->-->-ATF3->--<-BATF3-<--<-NSL1-<-->-TATDN3->--<-spermatogenesis-associated protein 45-L-<-->-FLVCR1-L->-

Kryptolebias marmoratus XP_017282868.1

-<-MYT1LA-<--<-RNASEH1-<--<-CEBPZ-<--<-CNKSR3-<--<-NDUFAF7-<-//-<-NMBR-<-->-GJE1->-->-VTA1->-->-ADGRG6->-//->-TNFAIP2A->-->-MAP/microtubule affinity-regulating kinase 3->--<-creatine kinase B-type-<--<-photoreceptor outer segment membrane glycoprotein 2-<--<-NLRC3-<--<-NLRC12-L-<-->-CDC42BPB->--<-GPR132P-<--<-VIPAS39-<-->-AHSA1->--<-HIVEP2-<-->-AIG1->--<-ADAT2-<-->-PEX3->-->-ATF3->--<-NSL1-<-->-TATDN3->-->-FLVCR1->-//->-VASH2->--<-ANGEL2-<--<-GALNT14-<--<-ZFAND3-<--<-BTBD9-<-

Nematolebias whitei XP_037541896.1

-<-CEBPZ-<-->-NDUFAF7->--<-E2F6-<--<-MYT1L-<--<-MYT1L-<--<-RNASEH1-<-->-RPS7->-//-<-NMBR-<-->-GJE1->-->-VTA1->-->-ADGRG6->-//->-TNFAIP2A->-->-MAP/microtubule affinity-regulating kinase 3-L->--<-creatine kinase B-type-L-<--<-LBH-<-->-CDC42BPB->--<-GPR132P-<--<-VIPAS39-<-->-AHSA1->--<-HIVEP2-<-->-AIG1->--<-ADAT2-<-->-PEX3->-->-ATF3->--<-NSL1-<-->-TATDN3->-->-FLVCR1->-//->-VASH2->--<-ANGEL2-<--<-GALNT14-<--<-ZFAND3-<--<-BTBD9-<-

Siluriformes

Ictalurus punctatus XP_017348268.1

->-ABRACL->-->-HECA->--<-TXLNB-<-->-VTA1->-->-ADGRG6->--<-EDN-2-L-<--<-EHD3-<--<-ANGEL2-<--<-GALNT14-L-<--<-FLVCR1-<-->-spermatogenesis-associated protein 45-L->-->-BATF3->-->-NSL1->-//->-SLC30A3->--<-GTF3C2-<-->-phospholipase D1-L->--<-ZNF512-L-<-->-leucine-rich repeat flightless-interacting protein 2-L->--<-STUM-<--<-ITPKB-L-<-->-ADCK3->--<-HIVEP2-<-->-AIG1->--<-ADAT2-<-->-PEX3->-->-ATF3->-->-SMARCE1->-->-kinase C beta type->-->-voltage-dependent calcium channel gamma-3 subunit-L->-->-RBBP6->--<-TNRC6A-<--<-acyl carrier protein, mitochondrial-L-<-->-RPUSD1->--<-DCTN5-<--<-GNG13-<-->-TRAF7->-

Pangasianodon hypophthalmus XP_026773238.1

->-SLC30A3->--<-GTF3C2-<--<-ZNF512-<-->-leucine-rich repeat flightless-interacting protein 2-L->--<-STUM-<--<-ITPKB-<-->-COQ8AA->--<-HIVEP2-<-->-AIG1->--<-ADAT2-<-->-PEX3->-->-ATF3->--<-BATF3-<--<-NSL1-<--<-spermatogenesis-associated protein 45-L-<-->-FLVCR1->-->-GALNT14->-->-ANGEL2->-->-EHD3->-->-EDN-2->--<-ADGRG6-<--<-VTA1-<-->-TXLNB->--<-HECA-<--<-ABRACL-<-

Tachysurus fulvidraco XP_026993771.1

-<-uridine-cytidine kinase-L 1-<-->-zinc transporter 2-L->--<-GTF3C2-<--<-ZNF512-L-<-->-STUM->--<-ITPKB-<-->-COQ8AA->-->-AIG1->--<-ADAT2-<-->-PEX3->-->-ATF3->--<-BATF3-<--<-NSL1-<-->-FLVCR1-L->-->-GALNT14->-->-ANGEL2->-->-EHD3->-->-EDN-1-L->--<-ADGRG6-<--<-VTA1-<-->-TXLNB->--<-HECA-<--<-ABRACL-<-

---

Tetrapod

mammals

Primates

->-ECT2L->--<-REPS1-<-->-ABRACL->-->-HECA->--<-TXLNB-<--<-CITED2-<--<-NMBR-<-->-VTA1->-->-ADGRG6->--<-HIVEP2-<-->-AIG1->--<-ADAT2-<-->-PEX3->--<-FUCA2-<-->-PHACTR2->-->-LTV1->-ZC2HC1B->--<-PLAGL1-<--<-SF3B5-<-->-STX11->-->-UTRN->-

Tupaia

chinensis->-ECT2L->--<-REPS1-<-->-ABRACL->-->-HECA->--<-TXLNB-<--<-CITED2-<--<-NMBR-<-->-VTA1->-->-ADGRG6->--<-HIVEP2-<-->-AIG1->-another scaffold -<-ADAT2-<-->-PEX3->--<-FUCA2-<-->-PHACTR2->-->-LTV1->-ZC2HC1B->--<-PLAGL1-<--<-SF3B5-<-->-STX11->-->-UTRN->-

Lagomorpha

->-ECT2L->--<-REPS1-<-->-ABRACL->-->-HECA->--<-TXLNB-<--<-CITED2-<--<-NMBR-<-->-VTA1->-->-ADGRG6->--<-HIVEP2-<-->-AIG1->--<-ADAT2-<-->-PEX3->--<-FUCA2-<-->-PHACTR2->-->-LTV1->-ZC2HC1B->--<-PLAGL1-<--<-SF3B5-<-->-STX11->-->-UTRN->-

Rodentia

->-ECT2L->--<-REPS1-<-->-ABRACL->-->-HECA->--<-TXLNB-<--<-CITED2-<--<-NMBR-<-->-VTA1->-->-ADGRG6->--<-HIVEP2-<-->-AIG1->--<-ADAT2-<-->-PEX3->--<-FUCA2-<-->-PHACTR2->-->-LTV1->-ZC2HC1B->--<-PLAGL1-<--<-SF3B5-<-->-STX11->-->-UTRN->-

Carnivora

Ailuropoda melanoleuca, Canis lupus familiaris, Felis catus

->-ECT2L->--<-REPS1-<-->-ABRACL->-->-HECA->--<-TXLNB-<--<-CITED2-<--<-NMBR-<-->-VTA1->-->-ADGRG6->--<-HIVEP2-<-->-AIG1->--<-ADAT2-<-->-PEX3->--<-FUCA2-<-->-PHACTR2->-->-LTV1->-ZC2HC1B->--<-PLAGL1-<--<-SF3B5-<-->-STX11->-->-UTRN->-

Chiroptera

->-ECT2L->--<-REPS1-<-->-ABRACL->-->-HECA->--<-TXLNB-<--<-CITED2-<--<-NMBR-<-->-VTA1->-->-ADGRG6->--<-HIVEP2-<-->-AIG1->--<-ADAT2-<-->-PEX3->--<-FUCA2-<-->-PHACTR2->-->-LTV1->-ZC2HC1B->--<-PLAGL1-<--<-SF3B5-<-->-STX11->-->-UTRN->-

eulipotyphla

Erinaceus europaeus(western European hedgehog)

-<-NMBR-<-->-VTA1->-->-ADGRG6->--<-HIVEP2-<-->-AIG1->-//--<-ADIPO2-<--<-WNT5B-<--<-ERC1-<-

Condylura cristata(star-nosed mole), Sorex araneus(European shrew)

->-ECT2L->--<-REPS1-<-->-ABRACL->-->-HECA->--<-TXLNB-<--<-CITED2-<--<-NMBR-<-->-VTA1->-->-ADGRG6->--<-HIVEP2-<-->-AIG1->--<-ADAT2-<-->-PEX3->--<-FUCA2-<-->-PHACTR2->-->-LTV1->-ZC2HC1B->--<-PLAGL1-<--<-SF3B5-<-->-STX11->-->-UTRN->-

Xenarthra

->-ECT2L->--<-REPS1-<-->-ABRACL->-->-HECA->--<-TXLNB-<--<-CITED2-<--<-NMBR-<-->-VTA1->-->-ADGRG6->--<-HIVEP2-<-->-AIG1->--<-ADAT2-<-->-PEX3->--<-FUCA2-<-->-PHACTR2->-->-LTV1->-ZC2HC1B->--<-PLAGL1-<--<-SF3B5-<-->-STX11->-->-UTRN->-

Artiodactyla

->-ECT2L->--<-REPS1-<-->-ABRACL->-->-HECA->--<-TXLNB-<--<-CITED2-<--<-NMBR-<-->-VTA1->-->-ADGRG6->--<-HIVEP2-<-->-AIG1->--<-ADAT2-<-->-PEX3->--<-FUCA2-<-->-PHACTR2->-->-LTV1->-ZC2HC1B->--<-PLAGL1-<--<-SF3B5-<-->-STX11->-->-UTRN->-

Afrotheria

->-ECT2L->--<-REPS1-<-->-ABRACL->-->-HECA->--<-TXLNB-<--<-CITED2-<--<-NMBR-<-->-VTA1->-->-ADGRG6->--<-HIVEP2-<-->-AIG1->--<-ADAT2-<-->-PEX3->--<-FUCA2-<-->-PHACTR2->-->-LTV1->-ZC2HC1B->--<-PLAGL1-<--<-SF3B5-<-->-STX11->-->-UTRN->-

Notostraca

（Manis javanica(Malayan pangolin)）

->-NDUFS4->--<-ARL15-<-->-HSPB3->-->-HECA->--<-TXLNB-<--<-CITED2-<--<-NMBR-<-->-VTA1->-->-ADGRG6->--<-HIVEP2-<-->-AIG1->--<-ADAT2-<-->-PEX3->--<-FUCA2-<-->-PHACTR2->-->-LTV1->-ZC2HC1B->--<-PLAGL1-<--<-SF3B5-<-->-STX11->-->-UTRN->-

Manis pentadactyla(Chinese pangolin)

->-ECT2L->--<-REPS1-<-->-ABRACL->-->-HECA->--<-TXLNB-<--<-CITED2-<--<-NMBR-<-->-VTA1->-->-ADGRG6->--<-HIVEP2-<-->-AIG1->--<-ADAT2-<-->-

PEX3->--<-FUCA2-<-->-PHACTR2->-->-LTV1->-ZC2HC1B->--<-PLAGL1-<--<-SF3B5-<-->-STX11->-->-UTRN->-

Ornithorhynchus anatinus

->-ECT2L->--<-REPS1-<-->-ABRACL->-->-HECA->--<-TXLNB-<--<-CITED2-<--<-NMBR-<-->-VTA1->-->-ADGRG6->--<-HIVEP2-<-->-AIG1->--<-ADAT2-<-->-PEX3->--<-FUCA2-<-->-PHACTR2->-->-LTV1->-ZC2HC1B->--<-PLAGL1-<--<-SF3B5-<-->-STX11->-->-UTRN->-

Metatheria

->-ECT2L->--<-REPS1-<-->-ABRACL->-->-HECA->--<-TXLNB-<--<-CITED2-<--<-NMBR-<-->-VTA1->-->-ADGRG6->--<-HIVEP2-<-->-AIG1->--<-ADAT2-<-->-PEX3->--<-FUCA2-<-->-PHACTR2->-->-LTV1->-ZC2HC1B->--<-PLAGL1-<--<-SF3B5-<-->-STX11->-->-UTRN->-

Reptiles

Lepidosauria

Anolis carolinensis (green anole)

->-FILIP1->-->-TMEM30A->-->-Col12a1->-//-<-NMBR-<-->-VTA1->-->-ADGRG6->--<-HIVEP2-<-->-AIG1->--<-ADAT2-<-->-PEX3->--<-FUCA2-<-//->-AKAP7->--<-MED23-<-->-ENPP3->-

Gekko japonicus

->-NCAM1->-->-TTC12->-//->-ADGRG6->--<-HIVEP2-<-->-AIG1->--<-ADAT2-<-->-PEX3->--<-FUCA2-<-->-PHACTR2->-->-LTV1->-ZC2HC1B->--<-PLAGL1-<-//-<-SFB5-<-STX11->-->-UTRN->-

Lacerta agilis Podarcis muralis Pogona vitticeps Zootoca vivipara Notechis scutatus Pantherophis guttatus Protobothrops mucrosquamatus Pseudonaja textilis Thamnophis elegans

->-ARFGEF3->--<-NHSL1-<-->-CCDC28A->--<-REPS1-<-->-ABRACL->-->-HECA->--<-TXLNB-<--<-CITED2-<--<-NMBR-<-->-VTA1->-->-ADGRG6->--<-HIVEP2-<-->-AIG1->--<-ADAT2-<-->-PEX3->--<-FUCA2-<-->-PHACTR2->-->-LTV1->-ZC2HC1B->--<-PLAGL1-<-//-<-SFB5-<-STX11->-->-UTRN->-

Birds

->-ARFGEF3->-->-SMIM28->--<-NHSL1-<-->-CCDC28A->--<-REPS1-<-->-ABRACL->-->-HECA->--<-TXLNB-<--<-CITED2-<--<-NMBR-<-->-VTA1->-->-

ADGRG6->--<-HIVEP2-<-->-AIG1->--<-ADAT2-<-->-PEX3->--<-FUCA2-<-->-PHACTR2->-->-LTV1->-ZC2HC1B->--<-PLAGL1-<--<-RSPH3-<--<-TAGAP-<-

Repetiles

Crocodylia

Alligatoridae Alligator mississippiensis (American alligator)

-<-CFHR1-<-//-<-NMBR-<-->-VTA1->-->-ADGRG6->--<-HIVEP2-<-->-AIG1->--<-ADAT2-<-->-PEX3->--<-FUCA2-<-->-PHACTR2->-->-LTV1->-ZC2HC1B->--<-PLAGL1-<-//-<-SFB5-<-STX11->-->-UTRN->-

Longirostres （Crocodylus porosus Gavialis gangeticus） Alligatoridae(Alligator sinensis) Testudines

->-ARFGEF3->--<-NHSL1-<-->-CCDC28A->--<-REPS1-<-->-ABRACL->-->-HECA->--<-TXLNB-<--<-CITED2-<--<-NMBR-<-->-VTA1->-->-ADGRG6->--<-HIVEP2-<-->-AIG1->--<-ADAT2-<-->-PEX3->--<-FUCA2-<-->-PHACTR2->-->-LTV1->-ZC2HC1B->--<-PLAGL1-<-//-<-SFB5-<-STX11->-->-UTRN->-

Testudines

Chrysemys picta (Painted turtle)

SMIM28->--<-NHSL1-<-->-CCDC28A->--<-REPS1-<-->-ABRACL->-->-HECA->--<-TXLNB-<--<-CITED2-<--<-NMBR-<-->-VTA1->-->-ADGRG6->--<-HIVEP2-<-->-AIG1->--<-ADAT2-<-->-PEX3->--<-FUCA2-<-->-PHACTR2->-->-LTV1->-ZC2HC1B->--<-PLAGL1-<-//-<-SFB5

Amphibian

Xenopus tropicalis Xenopus laevis Nanorana parkeri

->-SERINC1.1->--<-HSF2-<--<-HSF2.2-<-->-LCA5->--<-HIVEP2-<-->-AIG1->--<-ADAT2-<-->-PEX3->--<-FUCA2-<-->-PHACTR2->-->-LTV1->-//->-TAGAP->-->-RSPH3->-->-EZR->-

Gymnophiona

->-MAP3K5->-->-MAP7->--<-Vacuolar protein 8-Like-<-->-VTA1->-->-ADGRG6->--<-HIVEP2-<-->-AIG1->--<-ADAT2-<-->-PEX3->--<-FUCA2-<-->-PHACTR2->-->-LTV1->-->-ZC2HC1B->-//->--<-HINT3-<--<-NCOA7-<--<-HEY2-<_

ADTRP

Fish

coelacanth XP_014350550.1

-->-ADTRP->--<-TMEM170B-<--<-LOC106705504-<-

Chondrichthyes

Callorhinchus milii XP_007904571.1

->-PRKAG2->-->-RHEB->-->-CRYGN->-->-TBC1D7->--<-PHACTR1-<--<-EDN1-<--<-HIVEP1-<-->-ADTRP->-->-TMEM170B->-->-NEDD9->--<-SMIM13-<--<-GFOD1-<-

Amblyraja radiata XP_032870910.1

-<-TMEM108-<--<-tRNA selenocysteine 1-associated protein 1-L-<-->-PAKLIP1->-->-TMEM14C->--<-MAK-<--<-GCM2-<--<-ELOVL2-<-->-TBC1 domain family member 7->--<-PHACTR1-L-<--<-HIVEP1-<-->-ADTRP->--<-trafficking kinesin-binding protein 1-L-<-->-ULK4->--<-CTNNB1-<--<-KIAA1143-<-->-KIF15->-->-TMEM42->--<-ZDHHC-<-->-EXOSC7->--<-CDCP1-<-->-FBXL7->-

Spotted gar

spotted gar XP_015213085.1

-<-NKD2-<--<-TRIP13-<-->-BRD9->-->-ZDHHC11->-->-TPPP->--<-CEP72-<-->-SLC9A3->--<-SLC9A3-<--<-EDN1-<--<-HIVEP1-<-->-ADTRP->--<-TMEM170B-<-->-NEDD9->--<-SMIM13-<--<-NFATC1-<--<-ATP9B-<--<-SALL3-<--<-TRAPPC9-<-->-CHRAC1->--<-AGO2-<-

Polypteriformes XP_028673164.1

->-fucolectin-L->--<-ZDHHC11-<--<-BRD9-<-->-TRIP13->-->-NKD2->--<-EDN1-<--<-HIVEP1-<-->-ADTRP->--<-TMEM170B-<-->-NEDD9->--<-SMIM13-<-->-zinc fingers and homeoboxes protein 1-L->-->-C8orf76-L->-->-fibrous sheath CABYR-binding protein-L->--<-RIDA-<-->-FBXO43->-->-regulator of G-protein signaling 22-L->-->-regulator of G-protein signaling 22-L->-

Eupercaria incertae sedis 目 XP_035519679.1

-<-PLPP4-<-->-CDH23->--<-C10orf105-L-<--<-VSIR-<--<-eif3l-<-->-SMOC2->-->-DACT2->-->-IRRFIP2->--<-MLH1-<-->-FAM83HA->-->-ADTRP->-->-HERC4->--<-COX5B-<-->-biotinidase-L->--<-TTL-<-->-FIGNL1->--<-IKZF1-<--<-SPATA48-<--<-VWC-<--<-poly(rC)-binding protein 3-L-<-->-MOCOS->-

Spariformes

Acanthopagrus latus XP_036978538.1

-<-PLPP4-<-->-CDH23->--<-C10orf105-L-<--<-VSIR-<--<-eif3l-<-->-SMOC2->-->-DACT2->-->-IRRFIP2->--<-MLH1-<-->-FAM83HA->->-ADTRP->-->-HERC4->--<-COX5B-<--<-TTL-<-->-FIGNL1->--<-IKZF1-<--<-SPATA48-<--<-VWC2-<--<-poly(rC)-binding protein 3-L-<-->-MOCOS->-

Sparus aurata XP_030297631.1

-<-PLPP4-<-->-CDH23->--<-C10orf105-<--<-VSIR-<--<-eif3l-<-->-SMOC2->-->-DACT2-L->-->-IRRFIP2->--<-MLH1-<-->-FAM83HA-L->-->-ADTRP->-->-HERC4->--<-COX5B-L-<--<-TTL-<-->-FIGNL1->--<-IKZF1-<--<-SPATA48-<--<-VWC2-<--<-poly(rC)-binding protein 3-L-<-->-MOCOS->-

Cichliformes

Haplochromis burtoni、*Pundamilia nyererei XP_005927155.2*、*XP_005742530.1*

-<-PPAPDC1A-<-->-CDH23->--<-C10orf105-<--<-VSIR-<--<-eif3l-<-->-SMOC2->-->-DACT2->-->-IRRFIP2->--<-MLH1-<-->-FAM83HA-L->-->-ADTRP->-->-HERC4->--<-COX5B-L-<--<-DNAJC12-<-->-SIRT1->-->-CACUL1->-->-MYPN-L->-->-ATOH7->-->-PKD2L1->--<-TIAL1-<-->-BAG3->-->-INPP5F->-

Oreochromis niloticus XP_005473908.1

-<-PLPP4-<-->-CDH23->--<-C10orf105-<--<-VSIR-<--<-eif3l-<-->-SMOC2->-->-DACT2->-->-IRRFIP2->--<-MLH1-<-->-FAM83HA->-->-ADTRP->-->-HERC4->--<-COX5B-<--<-DNAJC12-<-->-SIRT1->-->-CACUL1->-->-MYPN->-->-ATOH7->-->-PKD2L1->--<-TIAL1-<-->-BAG3->-->-INPP5F->-

Oreochromis aureus XP_031593389.1

-<-PLPP4-<-->-CDH23->--<-C10orf105-<--<-VSIR-<--<-eif3l-<-->-SMOC2->-->-DACT2->-->-IRRFIP2->--<-MLH1-<-->-FAM83HA->-->-ADTRP->-->-HERC4->--<-COX5B-<--<-homeodomain-interacting protein kinase 3-L-<--<-DNAJC12-<-->-SIRT1->-->-CACUL1->-->-MYPN->-->-ATOH7->-->-PKD2L1->--<-TIAL1-<-->-BAG3->-->-INPP5F->-

Maylandia zebra、*Neolamprologus brichardi XP_012776887.2*、*XP_035767095.1*

-<-PLPP4-<-->-CDH23->--<-C10orf105-L-<--<-VSIR-<--<-eif3l-<-->-SMOC2->-->-DACT2->-->-IRRFIP2->--<-MLH1-<-->-FAM83HA->-->-ADTRP->-->-HERC4->--<-COX5B-<--<-DNAJC12-<-->-SIRT1->-->-CACUL1->-->-MYPN->-->-ATOH7->-->-PKD2L1->--<-TIAL1-<-->-BAG3->-->-INPP5F->-

Pleuronectiformes

Hippoglossus hippoglossus XP_034463855.1

-<-PLPP4-<-->-CDH23->--<-VSIR-<--<-eif3l-<-->-SMOC2->-->-DACT2->-->-IRRFIP2->--<-MLH1-<-->-FAM83HA->-->-ADTRP->-->-HERC4->--<-COX5B-<--<-DNAJC12-<-->-SIRT1->-->-CACUL1->-->-MYPN->-->-ATOH7->-->-PKD2L1->--<-TIAL1-<-->-BAG3->-->-INPP5F->-

Hippoglossus stenolepis XP_035028051.1

-<-PLPP4-<-->-cadherin-23-L->-->-cadherin-23-L->-->-CDH23->--<-VSIR-<--<-eif3l-<-->-SMOC2->-->-DACT2->-->-IRRFIP2->--<-MLH1-<-->-FAM83HA->-->-ADTRP->-->-HERC4->--<-COX5B-<--<-DNAJC12-<-->-SIRT1->-->-CACUL1->-->-MYPN->-->-ATOH7->-->-PKD2L1->--<-TIAL1-<-->-BAG3->-->-INPP5F->-

Scophthalmus maximus XP_035461622.1

-<-PLPP4-<-->-CDH23->--<-C10orf105-L-<--<-VSIR-<--<-eif3l-<-->-SMOC2->-->-DACT2->-->-IRRFIP2->--<-MLH1-<-->-FAM83HA->-->-ADTRP->-->-HERC4->--<-COX5B-<--<-DNAJC12-<-->-SIRT1->-->-CACUL1->-->-MYPN->-->-ATOH7->-->-PKD2L1->--<-TIAL1-<-->-BAG3->-->-INPP5F->-

Cynoglossus semilaevis XP_008325807.1

-<-PLPP4-<-->-gamma-B2-L->-->-CDH23->--<-C10orf105-<--<-VSIR-<--<-eif3l-<-->-SMOC2->-->-DACT2-L->-->-IRRFIP2->--<-MLH1-<-->-FAM83HA-L->-->-ADTRP->-->-HERC4->--<-COX5B-L-<--<-DNAJC12-<-->-SIRT1->--<-MICU3B-L-<-->-FGF20B-L->--<-HES7-L-<-->-HES7->--<-PFDN6-<-->-MMGT1->--<-INTS6L-<-->-MOSPD1->-

Paralichthys olivaceus XP_019945632.1

-<-PLPP4-<-->-CDH23->--<-C10orf105-L-<-->-eukaryotic translation initiation factor 1->--<-VSIR-<--<-eif3l-<-->-SMOC2->-->-DACT2-L->-->-IRRFIP2->--<-MLH1-<-->-FAM83HA-L->-->-ADTRP->-->-HERC4->--<-COX5B-L-<--<-DNAJC12-<-->-SIRT1->-->-CACUL1->-->-MYPN->-->-ATOH7->-->-PKD2L1->--<-TIAL1-<-->-BAG3->-->-INPP5F->-

Ovalentaria incertae sedis**目**

Amphiprion ocellaris XP_023132901.2

->-DACT2->-->-IRRFIP2->--<-MLH1-<-->-FAM83HA->-->-ADTRP->-->-HERC4->--
<-COX5B-<--<-DNAJC12-<-->-SIRT1->-->-CACUL1->-->-MYPN->-->-ATOH7->-->-
PKD2L1->--<-TIAL1-<-->-BAG3->-->-INPP5F->-

Anabantiforms

Anabas testudineus XP_026210052.1

-<-PLPP4-<-->-CDH23->--<-C10orf105-<--<-VSIR-<--<-eif3l-<-->-SMOC2->-->-
DACT2->-->-IRRFIP2->--<-MLH1-<-->-FAM83HA->-->-ADTRP->-->-HERC4->--<-
COX5B-<--<-DNAJC12-<-->-SIRT1->-->-CACUL1->-->-MYPN->-->-ATOH7->-->-
PKD2L1->--<-TIAL1-<-->-BAG3->-->-INPP5F->-

Betta splendens XP_029031623.1

-<-PLPP4-<-->-CDH23->--<-C10orf105-<--<-VSIR-<--<-eif3l-<-->-SMOC2->-->-
DACT2-L->-->-IRRFIP2->--<-MLH1-<-->-FAM83HA-L->-->-ADTRP->-->-HERC4-
>--<-COX5B-L-<--<-TTL-<-->-FIGNL1->--<-IKZF1-<--<-SPATA48-<--<-VWC2-<--
<-poly(rC)-binding protein 3-L-<-->-MOCOS->-

Gobiiformes

Periophthalmus magnuspinnatus XP_033835178.1

-<-PLPP4-<-->-CDH23->--<-VSIR-<--<-eif3l-<-->-SMOC2->-->-DACT2->-->-
IRRFIP2->--<-MLH1-<-->-FAM83HA->-->-ADTRP->-->-HERC4->--<-COX5B-<-->-
SGPL1->--<-PCBD1-<-->-heparan-alpha-glucosaminide N-acetyltransferase->-->-RET-
>-->-csgalnact2->--<-SEC23IP-<-->-MCMBP->--<-INPP5F-<--<-BAG3-<-->-TIAL1->-
-<-PKD2L1-<--<-ATOH7-<--<-MYPN-L-<--<-CACUL1-<--<-SIRT1-<-->-DNAJC12-
>-

Boleophthalmus pectinirostris XP_020782910.1

-<--DNAJC12-<-->-SIRT1->-->-CACUL1->-->-MYPN->-->-ATOH7->-->-PKD2L1->--
<-TIAL1-<-->-BAG3->-->-INPP5F->--<-MCMBP-<-->-SEC23IP->--<-csgalnact2-<--<-
RET-<--<-heparan-alpha-glucosaminide N-acetyltransferase-L-<-->-SGPL1->--<-
PCBD1-<-->-ADTRP->-->-HERC4->--<-COX5B-L-<-

Perciformes

Cyclopterus lumpus XP_034408410.1

-<-PLPP4-<-->-CDH23->--<-C10orf105-L-<--<-VSIR-<--<-eif3l-<-->-SMOC2->-->-DACT2->-->-IRRFIP2->--<-MLH1-<-->-FAM83HA->-->-ADTRP->-->-HERC4->--<-COX5B-L-<--<-L-threonine ammonia-lyase-<--<-serine/threonine-protein kinase 32C-L-<-->-leucine-rich repeat-containing protein 27-L->-->-PWWP2B->-->-inositol polyphosphate-5-phosphatase A->--<-NKX6.2-<-->-VALOPA->--<-cilia- and flagella-associated protein 46-<-

Epinephelus lanceolatus XP_033468680.1

-<-PLPP4-<-->-CDH23->--<-C10orf105-L-<--<-VSIR-<--<-eif3l-<-->-SMOC2->-->-DACT2->-->-IRRFIP2->--<-MLH1-<-->-FAM83HA->-->-ADTRP->-->-HERC4->--<-COX5B-<--<-TTL-<-->-FIGNL1->--<-IKZF1-<--<-SPATA48-<--<-VWC2-<--<-poly(rC)-binding protein 3-L-<-->-MOCOS->-

Etheostoma cragini、*Sebastes umbrosus XP_034754734.1*、*XP_037638162.1*

-<-PLPP4-<-->-CDH23->--<-C10orf105-L-<--<-VSIR-<--<-eif3l-<-->-SMOC2->-->-DACT2->-->-IRRFIP2->--<-MLH1-<-->-FAM83HA->-->-ADTRP->-->-HERC4->--<-COX5B-<--<-DNAJC12-<-->-SIRT1->-->-CACUL1->-->-MYPN->-->-ATOH7->-->-PKD2L1->--<-TIAL1-<-->-BAG3->-->-INPP5F->-

Sander lucioperca XP_035848411.1

-<-PLPP4-<-->-CDH23->--<-C10orf105-<--<-VSIR-<--<-eif3l-<-->-SMOC2->-->-DACT2->-->-IRRFIP2->--<-MLH1-<-->-FAM83HA->-->-ADTRP->-->-HERC4->--<-COX5B-<--<-DNAJC12-<-->-SIRT1->-->-CACUL1->-->-MYPN->-->-ATOH7->-->-PKD2L1->--<-TIAL1-<-->-BAG3->-->-INPP5F->-

Gymnodraco acuticeps XP_034080655.1

-<-PLPP4-<-->-CDH23->--<-C10orf105-L-<--<-VSIR-<--<-eif3l-<-->-SMOC2->-->-DACT2->-->-IRRFIP2->--<-MLH1-<-->-FAM83HA->-->-ADTRP->-->-HERC4->--<-COX5B-<--<-DNAJC12-<-->-SIRT1->-->-CACUL1->-->-MYPN->-->-ATOH7->-->-PKD2L1->--<-TIAL1-<-->-BAG3->-->-INPP5F->-

Pseudochaenichthys georgianus XP_033956067.1

-<-PLPP4-<-->-CDH23->--<-C10orf105-L-<--<-VSIR-<--<-eif3l-<-->-SMOC2->-->-DACT2->-->-IRRFIP2->--<-MLH1-<-->-FAM83HA->-->-ADTRP->-->-HERC4->--<-COX5B-<-->-FAM151B-L->--<-dihydrofolate reductase-L-<--<-DNAJC12-<-->-SIRT1->-->-CACUL1->-->-MYPN->-->-ATOH7->-->-PKD2L1->--<-TIAL1-<-->-BAG3->-->-INPP5F->-

Pungitius pungitius XP_037321329.1

-<-PLPP4-<-->-CDH23->--<-C10orf105-L-<--<-VSIR-<--<-eif3l-<-->-SMOC2->-->-DACT2->-->-IRRFIP2->--<-MLH1-<-->-FAM83HA->-->-ADTRP->-->-HERC4->--<-

DNAJC12-<-->-SIRT1->-->-CACUL1->-->-MYPN->-->-ATOH7->-->-PKD2L1->--<-TIAL1-<-->-BAG3->-->-INPP5F->-

Trematomus bernacchii XP_034008522.1

-<-PLPP4-<-->-CDH23->--<-C10orf105-L-<--<-VSIR-<--<-eif3l-<-->-SMOC2->-->-DACT2->-->-IRRFIP2->--<-MLH1-<-->-FAM83HA->-->-ADTRP->-->-HERC4->--<-COX5B-<--<-protein enabled homolog-<--<-cilia- and flagella-associated protein 100-L-<-->-SUN domain-containing protein 3-L->--<-DNAJC12-<-->-SIRT1->-->-CACUL1->-->-MYPN->-->-ATOH7->-->-PKD2L1->--<-TIAL1-<-->-BAG3->-->-INPP5F->-

Anarrhichthys ocellatus(wolf-eel)、Cottoperca gobio XP_031694986.1、XP_029306058.1

-<-PLPP4-<-->-CDH23->--<-C10orf105-<--<-VSIR-<--<-eif3l-<-->-SMOC2->-->-DACT2-L->-->-IRRFIP2->--<-MLH1-<-->-FAM83HA-L->-->-ADTRP->-->-HERC4->--<-COX5B-L-<--<-TTL-<-->-FIGNL1->--<-IKZF1-<--<-SPATA48-<--<-VWC2-<--<-poly(rC)-binding protein 3-L-<-->-MOCOS->-

Etheostoma spectabile XP_032379287.1

-<-PLPP4-<-->-CDH23->--<-C10orf105-<--<-VSIR-<--<-eif3l-<-->-SMOC2->-->-DACT2->-->-IRRFIP2->--<-MLH1-<-->-FAM83HA->-->-ADTRP->-->-HERC4->--<-COX5B-L-<-

Notothenia coriiceps XP_010764197.1

->-DACT2->-->-IRRFI-<-MLH1-L-<-->-MLH1-L->--<-MLH1-L-<-->-FAM83HA->-->-ADTRP->-->-HERC4->-

Batrachoidiformes

Thalassophryne amazonica XP_034040935.1

-<-zinc finger protein OZF-L-<--<-zinc finger protein 3 homolog-<-->-KIAA0753->--<-MYPN-L-<--<-MYPN-L-<--<-CACUL1-<-->-DACT2->-->-IRRFIP2->--<-MLH1-<-->-mucin-17-L->-->-ADTRP->-->-HERC4->--<-COX5B-<--<-DNAJC12-<-->-SIRT1->-->-ATOH7->-->-PKD2L1->--<-TIAL1-<-->-BAG3->-->-INPP5F->-

Esociformes

Esox lucius XP_010866473.2

-<-Hermansky-Pudlak syndrome 6-<-->-zinc finger BED domain-containing protein 4-L->-->-KCNIP2->--<-COX15-<-->-CUTC->--<-zinc finger BED domain-containing protein 1-L-<--<-ABCC2-<-->-IRRFIP2->--<-MLH1-<-->-FAM83HA->-->-ADTRP->--

>-HERC4->--<-COX5B-<--<-DNAJC12-<-->-SIRT1->-->-CACUL1->-->-MYPN->-->-ATOH7->-->-PKD2L1->--<-TIAL1-<-->-BAG3->-->-INPP5F->-

Elopiformes

Megalops cyprinoides XP_036409623.1

-<-KLHL24A-<-->-MUL1A->--<-probable G-protein coupled receptor 148-<--<-MTERF4-<--<-PASK-<--<-KLHL6-<--<-T cell receptor alpha variable 14/delta variable 4-L-<--<-T-cell receptor alpha chain V region RL-5-L-<--<-C8B-<-->-ADTRP->--<-TADA1-<-->-leucine-rich repeat-containing protein 52-L->--<-cytochrome P450 2J6-L-<--<-cytochrome P450 2J2-L-<--<-CZIB-<-->-ZTE38->-->-TMEM69->--<-actin-binding protein IPP-<--<-toll-L receptor 13-<--<-toll-L receptor 13-<-

Syngnathiformes

Hippocampus comes XP_019737598.1

-<-PLPP4-<-->-CDH23->--<-VSIR-<--<-eif3l-<-->-SMOC2->-->-DACT2-L->-->-IRRFIP2->--<-MLH1-<-->-FAM83HA-L->-->-ADTRP->-->-HERC4->--<-COX5B-L-<--<-DNAJC12-L-<--<-DNAJC12-L-<-->-SIRT1-L->-->-SIRT1-L->-->-CACUL1->-->-MYPN-L->-->-ATOH7-L->-->-PKD2L1->--<-TIAL1-<-->-BAG3->-->-INPP5F->-

Syngnathus acus XP_037128566.1

-<-DNAJC12-<-->-SIRT1->-->-CACUL1->-->-MYPN->-->-ATOH7->-->-PKD2L1->--<-TIAL1-<-->-BAG3->-->-INPP5F->-//-<-PLPP4-<-->-CDH23->--<-VSIR-<--<-eif3l-<-->-SMOC2->-->-DACT2->-->-IRRFIP2->--<-MLH1-<-->-FAM83HA->-->-ADTRP->-->-HERC4->--<-antigen peptide transporter 2-L-<-->-NDNFL->-->-DNAJC9->--<-FAM149B1-<-->-ECD->---si:ch211-248a14.8-<---P4HA1B->-->-ACTN2B->-->-RYR2B->-

Cyprinodontiformes

Xiphophorus couchianus、*Xiphophorus maculatu XP_027862196.1*、 *XP_023182702.1*

-<-PLPP4-<-->-CDH23->--<-VSIR-<--<-eif3l-<-->-SMOC2->-->-DACT2-L->-->-IRRFIP2->--<-MLH1-<-->-FAM83HA-L->-->-zinc finger protein OZF-L->-->-ADTRP->-->-HERC4->--<-COX5B-L-<--<-TTL-<-->-FIGNL1->--<-IKZF1-<--<-SPATA48-<--<-VWC2-<--<-poly(rC)-binding protein 3-L-<-->-MOCOS->-

Poecilia latipinna XP_014896437.1

-<-PLPP4-<-->-CDH23->--<-C10orf105-<--<-VSIR-<--<-eif3l-<-->-SMOC2->-->-DACT2-L->-->-IRRFIP2->--<-MLH1-<-->-FAM83HA-L->-->-zinc finger protein OZF-L->-->-ADTRP->-->-HERC4->--<-COX5B-L-<--<-TTL-<-->-FIGNL1->--<-IKZF1-<-

Poecilia mexicana XP_014848300.1

-<-PLPP4-<-->-CDH23->--<-C10orf105-<--<-VSIR-<--<-eif3l-<-->-SMOC2->-->-DACT2-L->-->-IRRFIP2->--<-MLH1-<-->-FAM83HA-L->-->-zinc finger protein OZF-L->-->-ADTRP->-->-HERC4->--<-COX5B-L-<--<-TTL-<-->-FIGNL1->--<-IKZF1-L-<-

Nothobranchius furzeri XP_015799352.1

-<-PLPP4-<-->-CDH23->--<-C10orf105-<--<-VSIR-<-->-DACT2->-->-IRRFIP2->--<-MLH1-<-->-FAM83HA-L->-->-ADTRP->-->-HERC4->--<-COX5B-L-<-->-MCMBP->--<-INPP5F-<--<-BAG3-<-->-TIAL1->--<-PKD2L1-<--<-ATOH7-<--<-MYPN-<--<-CACUL1-<--<-SIRT1-<-->-DNAJC12->-

Austrofundulus limnaeus XP_013860897.1

-<-PLPP4-<-->-CDH23->--<-C10orf105-<--<-VSIR-<--<-eif3l-<-->-SMOC2->-->-DACT2->-->-IRRFIP2->--<-MLH1-<-->-FAM83HA-L->-->-ADTRP->-->-HERC4->-->-DSEL->-->-cadherin-7-L->-->-epsin-2-L->--<-CDH7-<--<-IMPA1-L-<--<-IMPA1-<-->-ADP-ribosyl cyclase/cyclic ADP-ribose hydrolase 1-L->-->-ADP-ribosyl cyclase/cyclic ADP-ribose hydrolase 1-L->-->-TNFAIP3-interacting protein 3-L->-

Cyprinodon variegatus XP_015235773.1

-<-NDUFA6-<--<-TRIOBP-<--<-NOL12-<--<-SREBF2-<--<-CCDC134-<-->-IRRFIP2->--<-MLH1-<-->-FAM83HA-L->-->-ADTRP->-->-HERC4->--<-COX5B-L-<--<-TTL-<-->-FIGNL1->--<-IKZF1-<--<-SPATA48-<--<-VWC2-<--<-poly(rC)-binding protein 3-L-<-->-MOCOS->-

Xiphophorus hellerii XP_032408177.1

-<-PLPP4-<-->-CDH23->--<-VSIR-<--<-eif3l-<-->-SMOC2->-->-DACT2->-->-IRRFIP2->--<-MLH1-<-->-FAM83HA-L->-->-zinc finger protein OZF-L->-->-ADTRP->-->-HERC4->--<-COX5B-L-<--<-TTL-<-->-FIGNL1->--<-IKZF1-<--<-SPATA48-<--<-VWC2-<--<-poly(rC)-binding protein 3-L-<-->-MOCOS->-

Cyprinodon tularosa XP_038137360.1

-<-PLPP4-<-->-CDH23->--<-VSIR-<--<-eif3l-<-->-SMOC2->-->-DACT2->-->-IRRFIP2->--<-MLH1-<-->-FAM83HA->-->-ADTRP->-->-HERC4->--<-COX5B-<--<-TTL-<-->-FIGNL1->--<-IKZF1-<--<-VWC2-<--<-poly(rC)-binding protein 3-L-<-->-MOCOS->-

Fundulus heteroclitus XP_035981823.1

-<-PLPP4-<-->-CDH23->--<-VSIR-<--<-eif3l-<-->-SMOC2->-->-DACT2->-->-
IRRFIP2->--<-MLH1-<-->-FAM83HA->-->-ADTRP->-->-HERC4->--<-COX5B-<--<-
NACHT, LRR and PYD domains-containing protein 12-L-<--<-NLRC3-L-<-

Kryptolebias marmoratus XP_017279715.1

-<-PLPP4-<-->-CDH23->--<-VSIR-<--<-eif3l-<-->-SMOC2->-->-DACT2->-->-
IRRFIP2->--<-MLH1-<-->-FAM83HA->-->-ADTRP->-->-HERC4->--<-COX5B-<--<-
DNAJC12-<-->-SIRT1->-->-CACUL1->-->-MYPN->-->-ATOH7->-->-PKD2L1->-->-
BAG3->-->-INPP5F->-

Nematolebias whitei XP_037545558.1

-<-PLPP4-<-->-CDH23->--<-C10orf105-L-<--<-VSIR-<->-SMOC2->-->-DACT2->-->-
IRRFIP2->--<-MLH1-<-->-FAM83HA-L->-->-ADTRP->-->-eukaryotic translation
initiation factor 3 subunit L->-->-HERC4->--<-COX5B-<--<-DNAJC12-<-->-SIRT1->--
>-CACUL1->-->-MYPN->-->-ATOH7->-->-PKD2L1->-->-INPP5F->-

Poecilia reticulata XP_008428290.1

-<-PLPP4-<-->-CDH23->--<-VSIR-<--<-eif3l-<-->-SMOC2->-->-DACT2-L->-->-
IRRFIP2->--<-MLH1-<-->-FAM83HA-L->-->-gastrula zinc finger protein XlCGF57.1-
L->-->-ADTRP->-->-HERC4->--<-COX5B-L-<--<-TTL-<-->-FIGNL1->--<-IKZF1-<--
<-clg15h7orf72-<--<-VWC2-<--<-poly(rC)-binding protein 3-L-<-->-MOCOS->-

Danio rerio *XP_021336569.1*

->-HERC4->--<-COX5B2-<--<-DNAJC12-<-->-SIRT1->-->-CACUL1->-->-MYPN->-
//-<-DACT2-<--<-SMOC2-<-->-eif3l->-->-ZGC153073->--<-CDH23-<-->-CAPN2A->-
->-CAPN1A->--<-MRPL14-<-->-IRRFIP2->--<-MLH1-<-->-FAM83HA->-->-ADTRP-
>-->-BTAF1->--<-CPEB3-<-->-MARCH5->--<-IDE-<-->-KMO->--<-OPN3-<--<-
CDC42EP3-<-->-regulator of microtubule dynamics protein 2-L->--<-CYP1B1-<-->-
NANP->--<-POLRAB-<-

Labriformes

Notolabrus celidotus XP_034538167.1

-<-WDR11-<-->-CDH23->--<-eif3l-<-->-SMOC2->-->-DACT2->-->-IRRFIP2->--<-
MLH1-<-->-FAM83HA->-->-ADTRP->-->-HERC4->--<-COX5B-<-->-NID1A->-->-
LYST->-->-guanine nucleotide-binding protein G(I)/G(S)/G(O) subunit gamma-4->-->-
B3GALNT2->--<-GGPS1-<-->-ARID4B->-->-TOMM20A->-->-IRF2BP2A->-

Labrus bergylta XP_029133960.1

-<-PLPP4-<-->-CDH23->--<-C10orf105-L-<--<-VSIR-<--<-eif3l-<-->-SMOC2->-->-DACT2-L->-->-IRRFIP2->-->-IRRFIP2-L->--<-MLH1-<-->-FAM83HA-L->-->-ADTRP->-->-HERC4->--<-VPS8-<-->-zinc finger protein ZIC 4-L->-->-PCCA->--<-TM9FS2-<--<-PIEZO1-<-->-Cdt1-L->--<-APRT-L-<-->-thymidine kinase 2, mitochondrial-L->-

Characiformes

Colossoma macropomum XP_036431171.1

-<-CACUL1-<--<-SIRT1-<-->-DNAJC1->-->-COX5B->-->-gpatch11->-->-eif2ak2->--<-IRF2BP2A-<--<-TOMM20A-<--<-ARID4B-<-->-GGPS1->--<-MYPN-<-->-FAM83HA->-->-ADTRP->-->-HERC4->-->-LYST->-->-guanine nucleotide-binding protein G(I)/G(S)/G(O) subunit gamma-4->-->-B3GALNT2->--<-inhibitor of nuclear factor kappa-B kinase subunit alpha-<-->-PLAUA->-->-VCLA->--<-AP3M1-<-->-ADKA->-->-KAT6B->-

Astyanax mexicanus XP_007238580.2

->-RRBP1A->--<-CFL1L-<-->-CAPN2A-L->-->-CAPN1A-L->--<-MRPL14-<-->-IRRFIP2->--<-MLH1-<-->-FAM83HA-L->-->-ADTRP->--<-serine/threonine-protein kinase prk-2-L-<-->-HERC4->--<-IRF2BP2A-<--<-TOM20-<--<-ARID4B-<-->-GGPS1->--<-b3galnt2-<--<-GNG4-<--<-LYST-<--<-eif2ak2-<--<-gpatch11-<--<-COX5B-L-<--<-DNAJC12-<-->-SIRT1->-->-CACUL1->-->-MYPN->-

Gadiformes

Gadus morhua XP_030235332.1

-<-IDE-<-->-KMO->--<-OPN3-<--<-E3 ubiquitin-protein ligase TRIM39-L-<--<-disintegrin and metalloproteinase domain-containing protein 8-L-<--<-SPEF1-<--<-disintegrin and metalloproteinase domain-containing protein 8-L-<-->-serine/threonine-protein kinase pim-2-L->--<-IQANK1-<-->-FAM83HA-L->-->-ADTRP->-->-HERC4->--<-COX5B-L-<--<-DNAJC12-<-->-SIRT1->-->-CACUL1->-->-MYPN-L->-->-ATOH7->-->-PKD2L1->--<-TIAL1-<-->-BAG3->-->-INPP5F->-

Tetraodontiformes

Takifugu rubripes(torafugu) XP_011601213.2

-<-PLPP4-<-->-CDH23-L->--<-C10orf105-L-<--<-VSIR-<--<-eif3l-<-->-SMOC2->-->-DACT2-L->-->-IRRFIP2->--<-MLH1-<-->-FAM83HA->-->-ADTRP->-->-HERC4->--

<-COX5B-L-<--<-E3 ubiquitin-protein ligase RBBP6-L-<--<-TTL-<-->-FIGNL1->--<-IKZF1-<--<-SPATA48-<--<-VWC2-<--<-poly(rC)-binding protein 3-L-<-->-MOCOS->-

Carangiforms

Seriola dumerili XP_022599480.1

-<-PLPP4-<-->-CDH23-L->--<-VSIR-<--<-eif3l-<-->-SMOC2->-->-DACT2-L->-->-IRRFIP2->--<-MLH1-<-->-FAM83HA-L->-->-ADTRP->-->-HERC4->--<-COX5B-L-<--<-MRPS6-<-->-nucleolar and coiled-body phosphoprotein 1-L->--<-MICU1-<-->-MCU->-->-OIT3->--<-group XIIB secretory phospholipase A2-L-<--<-PDE10A-<-

Seriola lalandi dorsalis XP_023267390.1

-<-PLPP4-<-->-CDH23->--<-C10orf105-<--<-VSIR-<--<-eif3l-<-->-SMOC2->-->-DACT2->-->-IRRFIP2->--<-MLH1-<-->-FAM83HA-L->-->-FAM83HA-L->-->-ADTRP->-->-HERC4->--<-COX5B-L-<--<-MRPS6-<-->-nucleolar and coiled-body phosphoprotein 1-L->--<-MICU1-<-->-MCU->-->-OIT3->--<-group XIIB secretory phospholipase A2-L-<--<-PDE10A-<-

Echeneis naucrates XP_029377291.1

-<-PLPP4-<-->-CDH23-L->--<-VSIR-<--<-eif3l-<-->-SMOC2->-->-DACT2->-->-IRRFIP2->--<-MLH1-<-->-FAM83HA-L->-->-ADTRP->-->-HERC4->--<-COX5B-L-<--<-MRPS6-<-->-nucleolar and coiled-body phosphoprotein 1-L->--<-MICU1-<-->-MCU->-->-OIT3->--<-group XIIB secretory phospholipase A2-L-<--<-PDE10A-<-

Blenniiforms

Gouania willdenowi XP_028324785.1

-<-PLPP4-<-->-CDH23->--<-VSIR-<--<-eif3l-<-->-SMOC2->-->-DACT2->-->-IRRFIP2->--<-MLH1-<-->-FAM83HA-L->-->-ADTRP->-->-HERC4->--<-COX5B-L-<--<-DNAJC12-<-->-SIRT1->-->-E3 ubiquitin/ISG15 ligase TRIM25-L->--<-hyaluronan-binding protein 2-L-<--<- probable E3 ubiquitin-protein ligase TRIML1-<--<-ABLIM1-<-->-FAM160B1->--<-SRF-<--<-PTK7-<-

Salarias fasciatus XP_029962558.1

-<-PLPP4-<-->-CDH23->--<-C10orf105-<--<-VSIR-<--<-eif3l-<-->-SMOC2->-->-DACT2->-->-IRRFIP2->--<-MLH1-<-->-FAM83HA-L->-->-ADTRP->-->-HERC4->--<-COX5B-<-->-TEX30->-->-UGP2B-L->--<-PELI1B-<--<-ABCH1-L-<-->-LGALSL->-->-aftiphilin-L->-->-ACTR2->-

Anguilliformes

Anguilla anguilla(European eel) XP_035279916.1

->-shisa-L-2A->-->-TUT4->-->-LRR receptor-L serine/threonine-protein kinase RCH1->--<-transmembrane protein 125-L-<--<-doublesex- and mab-3-related transcription factor 1-L-<-->-lipoprotein receptor-related protein 8-L->--<-KN motif and ankyrin repeat domain-containing protein 1-L-<-->-zona pellucida sperm-binding protein 4-L->-->-zona pellucida sperm-binding protein 4-L->-->-angiopoietin-related protein 3-L->-->-ADTRP->-->-phosphoglucomutase-1-L->-->-forkhead box protein D3-L->-->-inactive tyrosine-protein kinase transmembrane receptor ROR1-L->-->-toll-L receptor 13->-->-toll-L receptor 13->-->-toll-L receptor 12->-->-LOC118230359->-->-inactive tyrosine-protein kinase transmembrane receptor ROR1-L->-->-toll-L receptor 12->-->-ribonucleoprotein PTB-binding 2-L->--<-tyrosine-protein kinase JAK1-L

Cichliformes

Archocentrus centrarchus, Astatotilapia calliptera XP_030604338.1, XP_026046157.1

-<-PLPP4-<-->-CDH23->--<-C10orf105-<--<-VSIR-<--<-eif3l-<-->-SMOC2->-->-DACT2-L->-->-IRRFIP2->--<-MLH1-<-->-FAM83HA-L->-->-ADTRP->-->-HERC4->--<-COX5B-L-<--<-DNAJC12-<-->-SIRT1->-->-CACUL1->-->-MYPN->-->-ATOH7->-->-PKD2L1->--<-TIAL1-<-->-BAG3->-->-INPP5F->-

Holocentriforme

Myripristis murdjan XP_029926230.1

-<-PLPP4-<-->-CDH23->--<-C10orf105-<--<-VSIR-<--<-eif3l-<-->-SMOC2->-->-DACT2-L->-->-IRRFIP2->--<-MLH1-<-->-FAM83HA-L->-->-ADTRP->-->-HERC4->--<-COX5B-L-<--<-DNAJC12-<-->-SIRT1->-->-CACUL1->-->-MYPN->-->-ATOH7->-->-PKD2L1->--<-TIAL1-<-->-BAG3->-->-INPP5F->-

Beloniformes

Oryzias latipes XP_023819042.1

-<-PLPP4-<-->-CDH23->--<-C10orf105-<--<-VSIR-<--<-eif3l-<-->-SMOC2->-->-DACT2->-->-IRRFIP2->--<-MLH1-<-->-FAM83HA->-->-ADTRP->-->-HERC4->--<-COX5B-<--<-DNAJC12-<-->-SIRT1->-->-CACUL1->-->-MYPN->-->-ATOH7->-->-PKD2L1->--<-TIAL1-<-->-BAG3->-->-INPP5F->-

Synbranchiformes

Mastacembelus armatus XP_026164922.1

-<-PLPP4-<-->-CDH23->--<-C10orf105-L-<--<-VSIR-<--<-eif3l-<-->-SMOC2->-->-DACT2-L->-->-IRRFIP2->--<-MLH1-<-->-FAM83HA-L->-->-ADTRP->-->-HERC4->--<-COX5B-L-<--<-TTL-<-->-FIGNL1->--<-IKZF1-<--<-SPATA48-<--<-VWC2-<--<-poly(rC)-binding protein 3-L-<-->-MOCOS->-

Osteoglossiformes

Scleropages formosus XP_018590092.1

->-MLH1->--<-IRRFIP2-<-->-GOLGA4->-->-ITGA9->-->-FIGNL1->--<-IKZF1-<--<-VWC2-<-->-ZNF830->--<-poly(rC)-binding protein 3-L-<-->-MOCOS->-->-ADTRP->-

Kurtiformes

Sphaeramia orbicularis XP_030012138.1

->-cytochrome P450 26B1-L->-->-D(1)-L dopamine receptor->-->-fibroblast growth factor-binding protein 2-L->-->-COL13A1->-->-alpha-synuclein-L->--<-cGMP-dependent protein kinase 2-L-<-->-IRRFIP2-L->-->-IRRFIP2-L->--<-MLH1-<-->-FAM83HA-L->-->-ADTRP->-->-HERC4->--<-COX5B-L-<-->- cytochrome P450 2J2-L->-->-cytochrome P450 2J2-L->-->-cytochrome P450 2J6-L->-->- cytochrome P450 2J2-L->--<-DNAJC12-<-->-SIRT1->-->-CACUL1->-->-MYPN->-->-ATOH7->-->-PKD2L1->--<-TIAL1-<-->-BAG3->-->-INPP5F->-

Clupeiformes

Clupea harengus XP_031435347.1

->-cytosolic phospholipase A2 zeta-L->-->-cytosolic phospholipase A2 zeta-L->--<-Sec16B-L-<--<-FGFR2-<--<-ATE1-<-->-IRRFIP2->--<-MLH1-<--<-IQANK1-<--<-putative IQ motif and ankyrin repeat domain-containing-<-->-FAM83HA-L->-->-ADTRP->-->-HERC4->--<-COX5B-L-<--<-DNAJC12-<-->-eif3l->-->-VSIR->-->-C10orf10->--<-CDH23-<--<-zinc finger protein 501-L-<-

Denticeps clupeoides XP_028825503.1

->-DRGX->--<-c2h10orf71-<--<-CALHM3-<--<-SFR1-<--<-FGFR2-<--<-FGFR2-<--<-ATE1-<-->-IRRFIP2->--<-MLH1-<-->-FAM83HA-L->-->-ADTRP->-->-HERC4->--<-COX5B-L-<--<-DNAJC12-<-->-eif3l->-->-VSIR->-->-nuclease-sensitive element-binding protein 1-L->--<-CDH23-<--<-PRXL2A-<-

Ovalentaria incertae sedis

Parambassis ranga XP_028278758.1

-<-PLPP4-<-->-CDH23->--<-C10orf105-L-<--<-VSIR-<--<-eif3l-<-->-SMOC2->-->-DACT2-L->-->-IRRFIP2->--<-MLH1-<-->-FAM83HA-L->-->-ADTRP->-->-HERC4->--<-COX5B-L-<--<-TTL-<-->-FIGNL1->--<-IKZF1-<--<-SPATA48-<--<-VWC2-<--<-poly(rC)-binding protein 3-L-<-->-MOCOS->-

Carangaria incertae sedis

Lates calcarifer XP_018541980.1

-<-PLPP4-<-->-CDH23->--<-VSIR-<--<-eif3l-<-->-SMOC2->-->-DACT2-L->-->-IRRFIP2->--<-MLH1-<-->-FAM83HA-L->-->-ADTRP->-->-HERC4->--<-COX5B-L-<-

Cyprinina

Carassius auratus(goldfish) XP_026113715.1

->-VSIR-L->--<-CDH23-L-<-->-CAPN2A-L->-->-CAPN1A-L->--<-MRPL14-<-->-IRRFIP2-L->--<-MLH1-<-->-FAM83HA-L->-->-ADTRP->-->-BTAF1->--<-CPEB3-L-<-->-MARCH5-L->--<-IDE-L-<-->-KMO-L->-

Cyprinus Carpio ENSCCRT00000066504.1

->- WD40 repeat-containing protein SMU1-like->-->-Trypsin-1-like-PINK1->-->-FAM83H-Like->-->-ADTRP->--<-CCR4-NOT transcription complex subunit 2 like-<-->-Calpain 2 catalytic subunit-like->-->-capn1a->--<-mrpl14-<-->-Irrfip2

Sinocyclocheilus grahami XP_016085624.1

->-VSIR-L->--<-CDH23-L-<-->-CAPN2A-L->-->-CAPN1A-L->--<-MRPL14-<-->-IRRFIP2-L->--<-MLH1-<-->-FAM83HA-L->-->-ADTRP->-->-BTAF1->--<-CPEB3-L-<-->-MARCH5->--<-IDE-L-<-->-KMO->-

Sinocyclocheilus rhinocerous XP_016417229.1

->-FAM83HA->-->-ADTRP->-->-BTAF1->--<-CPEB3-L-<-->-MARCH5-L->--<-IDE-L-<-->-KMO->-

Salmoniformes

Salmo trutta XP_029609120.1

->-RRP12->--<-anthrax toxin receptor 1-L-<-->-PRDM8-L->--<-GDF10A-L-<--<-tyrosine-protein phosphatase non-receptor type 13-L-<-->-KCNIP2->--<-ABCC2-<-->-IRRFIP2->--<-MLH1-<--<-IQANK1-<-->-FAM83HA-L->-->-ADTRP->-->-HERC4->--<-COX5B-L-<--<-DNAJC12-<-->-SIRT1-L->-->-CACUL1-L->-->-MYPN-L->-->-ATOH7->-->-PKD2L1->--<-TIAL1-<-->-BAG3-L->-->-INPP5F->-

Oncorhynchus mykiss(rainbow trout) XP_021462672.2

->-RRP12->--<-anthrax toxin receptor 1-<-->-PRDM8-L->--<-GDF10A-<--<-tyrosine-protein phosphatase non-receptor type 20-L-<--<-tyrosine-protein phosphatase non-receptor type 13-L-<-->-KCNIP2-L->--<-ABCC2-<-->-IRRFIP2->--<-MLH1-<--<-IQANK1-<-->-FAM83HA->-->-ADTRP->-->-HERC4->--<-COX5B-<--<-DNAJC12-<-->-SIRT1->-->-CACUL1->-->-MYPN->-->-ATOH7->-->-PKD2L1->--<-TIAL1-<-->-BAG3->-->-INPP5F->-

Oncorhynchus nerka XP_029525755.1

->-RRP12->--<-anthrax toxin receptor 1-L-<-->-PRDM8-L->--<-GDF10A-L-<--<-tyrosine-protein phosphatase non-receptor type 20-L-<-->-KCNIP2->--<-ABCC2-<-->-IRRFIP2->--<-MLH1-<--<-IQANK1-<-->-FAM83HA-L->-->-ADTRP->-->-HERC4->--<-COX5B-L-<--<-DNAJC12-<-->-SIRT1-L->-->-CACUL1-L->-->-MYPN-L->-->-ATOH7->-->-PKD2L1->--<-TIAL1-L-<-->-BAG3-L->-->-INPP5F->-

Oncorhynchus tshawytscha XP_024280153.1

->-RRP12->--<-anthrax toxin receptor 1-L-<-->-PRDM8-L->--<-GDF10A-L-<--<-tyrosine-protein phosphatase non-receptor type 13-L-<-->-KCNIP2->--<-ABCC2-<-->-IRRFIP2->--<-MLH1-<--<-IQANK1-<-->-FAM83HA-L->-->-ADTRP->-->-HERC4->--<-COX5B-L-<--<-DNAJC12-<-->-SIRT1-L->-->-CACUL1-L->-->-MYPN-L->-->-ATOH7->-->-PKD2L1->--<-TIAL1-L-<-->-BAG3-L->-->-INPP5F->-

Oncorhynchus keta XP_035609795.1

->-RRP12->--<-anthrax toxin receptor 1-L-<-->-PRDM8->--<-GDF10-L-<--<-tyrosine-protein phosphatase non-receptor type 13-L-<-->-KCNIP2-L->--<-ABCC2-<-->-IRRFIP2->--<-MLH1-<--<-IQANK1-<-->-FAM83HA-L->-->-ADTRP->-->-HERC4->--<-COX5B-<--<-DNAJC12-<-->-SIRT1-L->-->-CACUL1->-->-MYPN->-->-ATOH7->-->-PKD2L1->--<-TIAL1-<-->-BAG3->-->-INPP5F->-

Oncorhynchus kisutch XP_020335675.2

->-RRP12->--<-anthrax toxin receptor 1-<-->-PRDM8-L->--<-GDF10-L-<--<-tyrosine-protein phosphatase non-receptor type 13-<-->-KCNIP2-L->--<-ABCC2-<-->-IRRFIP2->--<-MLH1-<-->-FAM83HA->-->-ADTRP->-->-HERC4->--<-COX5B-<--<-DNAJC12-<-->-SIRT1->-->-CACUL1-L->-->-MYPN->-->-ATOH7->-->-PKD2L1->--<-TIAL1-<-->-BAG3->-->-INPP5F->-

Salmo salar XP_014009768.1

->-RRP12->--<-anthrax toxin receptor 1-L-<-->-PRDM8-L->--<-GDF10A-L-<--<-tyrosine-protein phosphatase non-receptor type 13-L-<-->-KCNIP2-L->--<-ABCC2-<-->-IRRFIP2->--<-MLH1-<--<-IQANK1-<-->-FAM83HA->-->-ADTRP->-->-HERC4->--<-COX5B-L-<--<-DNAJC12-<-->-SIRT1->-->-CACUL1-L->-->-MYPN->-->-ATOH7->-->-PKD2L1->--<-TIAL1-L-<-->-BAG3->-->-INPP5F->-

---

Tetrapoda

Mammals

Mus musculus、***rabbit***、***Primates***、***Artiodactyla***、***dog***、***cat***、***horse***

->-Dtnbp1->--<-Jarid2-<--<-Cd83-<--<-Rnf182f-<-->-Mcur1->-->-Ranbp9->--<-Nol7-<--<-Sirt5-<-->-Gfod1->-->-Tbc1d7->--<-Phactr1-<--<-Edn1-<--<-Hivep1-<-->-ADTRP->--<-TMEM170b-<-->-NEDD9->--<-SMIM13-<-->-Elovl2->--<-Sycp2l-<-->-GCM2->-->-Mak->--<-TMEM14C-<--<-Pak1ip1-<--<-GCNt2-<-->-Tfap2a->-->-Ofcc1->-->-Slc35b3->-

Microcebus murinus

->-Dtnbp1->--<-Jarid2-<--<-Cd83-<--<-Rnf182f-<-->-Mcur1->-->-Ranbp9->--<-Nol7-<--<-Sirt5-<-->-Gfod1->-->-Tbc1d7->--<-Phactr1-<--<-Edn1-<--<-Hivep1-<-->-ADTRP->--<-TMEM170b-<-->-NEDD9->--<-SMIM13-<-->-Elovl2->--<-Sycp2l-<-->-GCM2->-//

Tupaia belangeri

-<-Cd83-<--<-Rnf182f-<-->-Mcur1->-->-Ranbp9->--<-Nol7-<--<-Sirt5-<-->-Gfod1->-->-Tbc1d7->--<-Phactr1-<--<-Edn1-<--<-Hivep1-<-->-ADTRP->--<-TMEM170b-<-->-NEDD9->--<-SMIM13-<-->-Elovl2->--<-Sycp2l-<-->-GCM2->-->-Mak->--<-TMEM14C-<--<-Pak1ip1-<--<-GCNt2-<-->-Tfap2a->-->-Ofcc1->-->-Slc35b3->-

Rattus norvegicus

->-Dtnbp1->--<-Jarid2-<--<-SMIM13-<-->-Elovl2->--<-Sycp2l-<-->-GCM2->-->-Mak->--<-TMEM14C-<--<-Pak1ip1-<--<-GCNt2-<--<-Gcnt6-<--<-Edn1-<--<-Hivep1-<-->-ADTRP->--<-TMEM170b-<-->-NEDD9->-->-Phactr1->--<-Tbc1d7-<--<-Gfod1-<-->-Sirt5->-->-Nol7->--<-Ranbp9-<--<-Mcur1-<-->-Rnf182->-->-Cd83->-->-Tfap2a->-->-Ofcc1->--Slc35b->

Lagomorpha

->-Dtnbp1->--<-Jarid2-<--<-Cd83-<--<-Rnf182f-<-->-Mcur1->-->-Ranbp9->--<-Nol7-<--<-Sirt5-<-->-Gfod1->--<-Elovl2-<-->-SMIM13->--<-NEDD9-<-->-TMEM170b->--<-ADTRP-<-->-HIVEP1->-->-EDN1->-Phactr1->-->-GCM2->-->-Mak->--<-TMEM14C-<--<-Pak1ip1-<--<-GCNt2-<-->-Tfap2a->-->-Ofcc1->-->-Slc35b3->-

ferret

Ailuropoda melanoleuca（giantpanda）

-<-Cd83-<--<-Rnf182f-<-->-Mcur1->-->-Ranbp9->--<-Nol7-<--<-Sirt5-<-->-Gfod1->-->-Tbc1d7->--<-Phactr1-<--<-Edn1-<--<-Hivep1-<-->-ADTRP->--<-TMEM170b-<-->-NEDD9->--<-SMIM13-<-->-Elovl2->--<-Sycp2l-<-->-GCM2->-->-Mak->--<-TMEM14C-<--<-Pak1ip1-<--<-GCNt2-<-->-Tfap2a->-->-Ofcc1->-->-Slc35b3->-

Myotis lucifugus(little brown bat)microbat

-<-Edn1-<--<-Hivep1-<-->-ADTRP->--<-TMEM170b-<-->-NEDD9->--<-SMIM13-<-->-Elovl2->--<-Sycp2l-<-->-GCM2->-->-Mak->--<-TMEM14C-<--<-Pak1ip1-<--<-GCNt2-<-->-Tfap2a->-->-Ofcc1->-->-Slc35b3->-

Eptesicus fuscus(big brown bat) microbat

->-Dtnbp1->--<-Jarid2-<--<-Cd83-<--<-Rnf182f-<-->-Mcur1->-->-Ranbp9->--<-Nol7-<--<-Sirt5-<-->-Gfod1->-->-Tbc1d7->--<-Phactr1-<--<-Edn1-<--<-Hivep1-<-->-ADTRP->--<-TMEM170b-<-->-NEDD9->--<-SMIM13-<-->-Elovl2->--<-Sycp2l-<-->-GCM2->-->-Mak->--<-TMEM14C-<--<-Pak1ip1-<--<-GCNt2-<-->-Tfap2a->-->-Ofcc1->-->-Slc35b3->-

Pteropus vampyrus(large flying fox) megabat

->-ADTRP->--<-TMEM170b-<-->-NEDD9->--<-SMIM13-<-->-Elovl2->--<-Sycp2l-<-->-GCM2->-->-Mak->--<-TMEM14C-<--<-Pak1ip1-<--<-GCNt2-<-->-Tfap2a->-

Pteropus alecto(black flying fox)megabat

->-Dtnbp1->--<-Jarid2-<--<-Cd83-<--<-Rnf182f-<-->-Mcur1->-->-Ranbp9->--<-Nol7-<--<-Sirt5-<-->-Gfod1->-->-Tbc1d7->--<-Phactr1-<--<-Edn1-<--<-Hivep1-<-->-ADTRP->--<-TMEM170b-<-->-NEDD9->--<-SMIM13-<-->-Elovl2->--<-Sycp2l-<-->-GCM2->-->-Mak->--<-TMEM14C-<--<-Pak1ip1-<--<-GCNt2-<-->-Tfap2a->-->-Ofcc1->-->-Slc35b3->-

eulipotyphla

->-Dtnbp1->--<-Jarid2-<--<-Cd83-<--<-Rnf182f-<-->-Mcur1->-->-Ranbp9->--<-Nol7-<--<-Sirt5-<-->-Gfod1->-->-Tbc1d7->--<-Phactr1-<--<-Edn1-<--<-Hivep1-<-->-ADTRP->--<-TMEM170b-<-->-NEDD9->--<-SMIM13-<-//

Xenarthra

->-Dtnbp1->--<-Jarid2-<--<-Cd83-<--<-Rnf182f-<-->-Mcur1->-->-Ranbp9->--<-Nol7-<--<-Sirt5-<-->-Gfod1->-->-Tbc1d7->--<-Phactr1-<--<-Edn1-<--<-Hivep1-<-->-ADTRP->--<-TMEM170b-<-->-NEDD9->--<-SMIM13-<-->-Elovl2->--<-Sycp2l-<-->-GCM2->-->-Mak->--<-TMEM14C-<--<-Pak1ip1-<--<-GCNt2-<-->-Tfap2a->-->-Ofcc1->-->-Slc35b3->-

Afrotheria（Echinops telfairi）

->-Dtnbp1->--<-Jarid2-<--<-Cd83-<--<-Rnf182f-<-->-Mcur1->-->-Ranbp9->--<-Nol7-<--<-Sirt5-<-->-Gfod1->-->-Tbc1d7->--<-Phactr1-<--<-Edn1-<--<-Hivep1-<-->-ADTRP->--<-TMEM170b-<-->-NEDD9->--<-SMIM13-<-->-Elovl2->--<-Sycp2l-<-->-GCM2->-->-Mak->--<-TMEM14C-<--<-Pak1ip1-<--<-GCNt2-<-//

Elephantulus edwardii, Orycteropus afer afer

->-Dtnbp1->--<-Jarid2-<--<-Cd83-<--<-Rnf182f-<-->-Mcur1->-->-Ranbp9->--<-Nol7-<--<-Sirt5-<-->-Gfod1->-->-Tbc1d7->--<-Phactr1-<--<-Edn1-<--<-Hivep1-<-->-ADTRP->--<-TMEM170b-<-->-NEDD9->--<-SMIM13-<-->-Elovl2->--<-Sycp2l-<-->-GCM2->-->-Mak->--<-TMEM14C-<--<-Pak1ip1-<--<-GCNt2-<-->-Tfap2a->-->-Ofcc1->-->-Slc35b3->-

Loxodonta africana

->-Dtnbp1->--<-Jarid2-<--<-Cd83-<--<-Rnf182f-<-->-Mcur1->-->-Ranbp9->--<-Nol7-<--<-Sirt5-<-->-Gfod1->-->-Tbc1d7->--<-Phactr1-<--<-Edn1-<--<-Hivep1-<-->-ADTRP->--<-TMEM170b-<-->-NEDD9->--<-SMIM13-<-(another scaffold)->-Elovl2->--<-Sycp2l-<-->-GCM2->-->-Mak->--<-TMEM14C-<--<-Pak1ip1-<--<-GCNt2-<-->-Tfap2a->-->-Ofcc1->-->-Slc35b3->-

Notostraca

->-Dtnbp1->--<-Jarid2-<--<-Cd83-<--<-Rnf182f-<-->-Mcur1->-->-Ranbp9->--<-Nol7-<--<-Sirt5-<-->-Gfod1->-->-Tbc1d7->--<-Phactr1-<--<-Edn1-<--<-Hivep1-<-->-ADTRP->--<-TMEM170b-<-->-NEDD9->--<-SMIM13-<-->-Elovl2->--<-Sycp2l-<-->-GCM2->--->-Mak->--<-TMEM14C-<--<-Pak1ip1-<--<-GCNt2-<-->-Tfap2a->-->-Ofcc1->-->-Slc35b3->-

Ornithorhynchus anatinus

->-Dtnbp1->--<-Jarid2-<--<-Cd83-<--<-Rnf182f-<-->-Mcur1->-->-Ranbp9->--<-Nol7-<--<-Sirt5-<-->-Gfod1->-->-Tbc1d7->--<-Phactr1-<--<-Edn1-<--<-Hivep1-<-->-ADTRP->--<-TMEM170b-<-->-NEDD9->--<-SMIM13-<-->-Elovl2->--<-Sycp2l-<-->-GCM2->--->-Mak->--<-TMEM14C-<--<-Pak1ip1-<--<-GCNt2-<-->-Tfap2a->-->-Ofcc1->-->-Slc35b3->-

Metatheria（Monodelphis domestica、Trichosurus vulpecula、Vombatus ursinus）（Phascolarctos cinereus XP_020818694.1），（Sarcophilus harrisii XP_023352108.1 ）

->-Dtnbp1->--<-Jarid2-<--<-Cd83-<--<-Rnf182f-<-->-Mcur1->-->-Ranbp9->--<-Nol7-<--<-Sirt5-<-->-Gfod1->-->-Tbc1d7->--<-Phactr1-<--<-Edn1-<--<-Hivep1-<-->-ADTRP->--<-TMEM170b-<-->-NEDD9->--<-SMIM13-<-->-Elovl2->--<-Sycp2l-<-->-GCM2->--->-Mak->--<-TMEM14C-<--<-Pak1ip1-<--<-GCNt2-<-->-Tfap2a->-->-Ofcc1->-->-Slc35b3->-

Birds

Anas platyrhynchos(mallard), Anser cygnoides domesticus, Aythya fuligula Cygnus atratus (black swan)

-->-STARD5NL->--<-MLH-<--<-SLC12A7-<-->-Gfod1->-->-Tbc1d7->--<-Phactr1-<--<-Edn1-<--<-Hivep1-<-->-ADTRP->--<-TMEM170b-<-->-NUP153->--<-CCK-<--<-CX3CR1-<-->-SLC25A38->--<-TMC2-<-

Gallus gallus

->-Cap2->-->-FAM8A1->--<--<-NUP153-<--<-TRNAU1AP-<-->-Pak1ip1->-->-TMEM14C->-->-Gfod1->-->-Tbc1d7->--<-Phactr1-<--<-Edn1-<--<-Hivep1-<-->-ADTRP->--<-TMEM170b-<-->-NEDD9->-->-Elovl2->--<-Sycp2l-<-->-GCM2->-->-Mak->--<-TMEM14C-<--<-GCNt2-<-->-Tfap2a->-->-Ofcc1->-->-Slc35b3->-

Meleagris gallopavo

->-Cap2->-->-FAM8A1->--<--<-NUP153-<--<-TRNAU1AP-<-->-Pak1ip1->-->-TMEM14C->-->-Gfod1->-->-Tbc1d7->--<-Phactr1-<--<-Edn1-<--<-Hivep1-<-->-ADTRP->--<-TMEM170b-<-->-NEDD9->--<-SMIM13-<-->-Elovl2->--<-Sycp2l-<-->-GCM2->-->-Mak->--<-GCNt2-<-->-Tfap2a->-->-Ofcc1->-->-Slc35b3->-

Taeniopygia guttata

->-Cap2->-->-FAM8A1->--<--<-NUP153-<--<-TRNAU1AP-<-->-Pak1ip1->-->-TMEM14C->-->-Gfod1->-->-Tbc1d7->--<-Phactr1-<--<-Edn1-<--<-Hivep1-<-->-ADTRP->--<-TMEM170b-<-->-NEDD9->--<-SMIM13-<-->-Elovl2->--<-Sycp2l-<-->-GCM2->-->-Mak->--<-TMEM14C-<--<-GCNt2-<-->-Tfap2a->-->-Ofcc1->-->-Slc35b3->-

Reptiles

Anolis carolinensis

-<-Edn1-<--<-Hivep1-<-->-ADTRP->--<-TMEM170b-<-->-NEDD9->--<-SMIM13-<-->-Elovl2->--<-Sycp2l-<-->-GCM2->-->-Mak->--<-TMEM14C-<--<-GCNt2-<-->-Tfap2a->-->-Ofcc1->-->-Slc35b3->-

Chrysemys picta (Painted turtle)

->-Cap2->-->-FAM8A1->--<--<-NUP153-<--<-TRNAU1AP-<-->-Pak1ip1->-->-TMEM14C->-->-ADTRP->--<-TMEM170b-<-->-NEDD9->--<-SMIM13-<-->-Elovl2->--<-Sycp2l-<-->-GCM2->-->-Mak->--<-GCNt2-<-->-Tfap2a->-->-Ofcc1->-->-Slc35b3->-

Alligator mississippiensis

->-Cap2->-->-FAM8A1->--<--<-NUP153-<--<-TRNAU1AP-<-->-Pak1ip1->-->-TMEM14C->-->-Gfod1->-->-Tbc1d7->--<-Phactr1-<--<-Edn1-<--<-Hivep1-<-->-ADTRP->--<-TMEM170b-<-->-NEDD9->--<-SMIM13-<-->-Elovl2->--<-Sycp2l-<-->-GCM2->-->-Mak->--<-TMEM14C-<--<-GCNT2

Amphibian Batrachia

Xenopus laevis

->-Dtnbp1->--<-Jarid2-<--<-Cd83-<--<-Rnf182f-<-->-Mcur1->-->-Ranbp9->--<-Nol7-<--<-Sirt5-<-->-Gfod1->-->-Tbc1d7->--<-Phactr1-<--<-Edn1-<--<-Hivep1-<-->-ADTRP-L->--<-TMEM170b-<-->-NEDD9->-->-SMIM13->-->-Elovl2->--<-Sycp2l-<-->-GCM2->-->-Mak->--<-TBC1D7-<-->-TMEM14C-<--<-GCNt2-<-->-Dtnbp1->--<-Jarid2-<--<-Cd83-<--<-Rnf182f-<-->-Mcur1->-->-Ranbp9->--<-Nol7-<--<-Sirt5-<-->-Gfod1->--<-Phactr1-<--<-Edn1-<--<-Hivep1-<-->-ADTRP-S->--<-TMEM170b-<-->-NEDD9->-->-SMIM13->-->-Elovl2->-

Xenopus tropicalis

->-Dtnbp1->--<-Jarid2-<--<-Cd83-<--<-Rnf182f-<-->-Mcur1->-->-Ranbp9->--<-Nol7-<-
-<-Sirt5-<-->-Gfod1->-->-Tbc1d7->--<-Phactr1-<--<-Edn1-<--<-Hivep1-<-->-ADTRP-
>--<-TMEM170b-<-->-NEDD9->--<-SMIM13-<-->-Elovl2->--<-Sycp2l-<-->-GCM2->-
->-Mak->-->-TMEM14C->--<-PAK1IP1-<--<-GCNt2-<-->-Tfap2a->-->-Ofcc1->-->-
Slc35b3->

AIG-L

Fish

Lampetra japonicum

Petromyzon marinus XP_032829735.1

-<-DCTD-<--<-IK-<--<-GRB2-associated-binding protein 1-L-<-->-FGG->--<-ARHGAP6-L-<--<-EDN-3-<--<-JMY-L-<--<-HIVEP3-<-->-AIG-L->-->-cAMP-dependent protein kinase inhibitor alpha-L->-->-tribbles homolog 2-L->--<-LRATD2-L-<--<-MYC2-<--<-DDX1-<-->-RTCA->-->-venom phosphodiesterase 1-L->--<-CCN2-<-

Chondrichthyes

Amblyraja radiata XP_032897540.1

-<-ZNF335-<--<-MMP9-<-->-SCL12A5->--<-NCOA5-<--<-TNNC2-<-->-SNX21->-->-NEURL2->-->-MYB120-L->--<-SYCP2-<--<-EDN-3-L-<-->-AIG-L->--<-TTLL9-<--<-WFDC3-<-->-regulator of G-protein signaling 9-binding protein-L->--<-UBE2C-<--<-DNTTIP1-<-->-BLCAP->--<-SRC-<--<-MANBAL-<-

Callorhinchus milii XP_007882691.1

-<-UBE2C-<--<-DNTTIP1-<--<-PCIF1-<-->-bone morphogenetic protein 7-L->-->-EAPP->-->-SYCP2->--<-PHACTR3-<--<-free fatty acid receptor 2-L-<--<-EDN-3-L-<--<-ZNF831-<-->-AIG-L->-->-PPP1R3D->--<-TTLL9-<-->-RAE1->-->-RBM38-L->-->-PCK1-L->--<-hosphoenolpyruvate carboxykinase, cytosolic [GTP]-L-<-->-GNAS->-->-NELFCD->--<-CTSZ-<--<-SYS1-<--<-PMEPA1-<-

***Coelacanth** XP_005993636.1*

-<-CDH26-L-<--<-cadherin-L protein 26-<--<-FAM217B-<-->-PPP1R3D->-->-SYCP2->-->-PDRG1-L->--<-PHACTR1-<--<-EDN3--<--<-ZNF813-<-->-AIG-L->-->-AURKB-L->-->-PRELID3B->-->-ATP5E->--<-TUBB1-<-->-CTSZ->--<-NELFCD-<--<-GNAS-<-->-piezo-type mechanosensitive ion channel component 2-L->--<-NPEPL1-<--<-STX16-<-

Polypteriformes XP_028676644.1

->-perlwapin-L->--<-PIGT-<-->-PHACTR3->--<-PPP1R3D-<-->-FAM217B->-->-PDRG1->--<-probable tubulin polyglutamylase TTLL9-<--<-TTLL9-<--<-EDN3-<--<-ZNF831-<-->-AIG-L->-->-CSTF1->-->-CASS4->--<-RBM38-<--<-RAE1-<--<-SPO11-

<-->-BMP7->-->-dual specificity protein phosphatase 15-L->-->-IRF10-L->-->-dysbindin-L->-

Spotted garspotted gar XP_006639616.2

-<-PTGIS-<-->-SNX21->--<-ACOT8-<-->-WFDC2->--<-PIGT-<-->-PHACTR3->--<-SYCP2-<--<-PPP1R3D-<-->-FAM217B->-->-PDRG1->--<-TTLL9-<--<-EDN3-<--<-ZNF831-<-->-AIG-L->-->-CSTF1->-->-CASS4->--<-RBM38-<--<-RAE1-<--<-SPO11-<-->-BMP7->-->-IRF10->-->-dysbindin-L->-->-cadherin-L protein 26->-

Danio rerio

Danio rerio(Danio rerio) NP_001017719.1

-<-AAR2-<-->-translocating chain-associated membrane protein 1-L 1->-->-ARL6IP5B->-->-SRSF6A->-->-si:ch211-10d23.5->-->-L3MBTL1->-->-SGK2A->-->-MYBL2A->-->-AIG-L->-->-PTPDC1B->--<-FBXW12-<-->-WNT7A-L->-->-NUP210->-->-IQSEC1-L->-->-IQSEC1A->-->-GP9->--<-CNBP-<-->-RAF1A->--<-SYN2A-<-

Siluriformes

Ictalurus punctatus XP_017343453.1

-<-MAFB-<--<-RNF114-<-->-SPATA2->-->-OPN7B->-->-trichohyalin-L->--<-NUP210-<-->-FBLN2->--<-WNT7AB-<-->-FBXW12->-->-AIG-L->-->-PTPDC1B-L->-->-bifunctional apoptosis regulator-L->-->-PRAF3->-->-SRSF6-L->-->-L3MBTL1->-->-SGK2->--<-nuclear receptor coactivator 3-L-<--<-translocating chain-associated membrane protein 1-L 1-<-->-AAR2->-->-EMILIN-3-L->-

Pangasianodon hypophthalmus XP_026800702.2

->-PPARG-L->--<-RAF1A-<-->-CNBPA->--<-GP9-<--<-IQSEC1A-<--<-NUP210-<-->-FBLN2-L->--<-WNT7AB-<-->-FBXW12->-->-MYBL2A->-->-AIG-L->-->-PTPDC1B->-->-bifunctional apoptosis regulator-L->-->-ARL6IP5B->-->-SRSF6->-->-L3MBTL1->-->-SGK2->--<-nuclear receptor coactivator 3-L-<--<-translocating chain-associated membrane protein 1-L 1-<-->-AAR2->-->-EMILIN-3-L->-

Tachysurus fulvidraco XP_027019397.1

-<-WNT7AB-L-<-->-FBXW12->-->-AIG-L->-->-PTPDC1B-L->-->-bifunctional apoptosis regulator-L->-->-PRAF3-L->-->-SRSF6-L->--<-PCDH18-<--<-SLC7A11-<-->-fibroblast growth factor receptor-L 1->-

Characiformes

Astyanax mexicanus XP_022532505.1

-<-EMILIN-3-<-->-cell surface glycoprotein 1-L->--<-AAR2-<-->-translocating chain-associated membrane protein 1-L 1->-->-nuclear receptor coactivator 3-L->-->-bifunctional apoptosis regulator-L->-->-PRAF3->-->-SRSF6-L->-->-L3MBTL1->-->-SGK2->-->-AIG-L->-->-PTPDC1B-L->--<-FBXW12-<-->-WNT7A->--<-FBLN2-L-<--<-FBLN2-L-<-->-NUP210->-->-IQSEC1A-L->-->-GP9->--<-CNBPA-L-<--<-RAF1A-L-<--<-PPARG-L-<--<-SYN2A-L-<-

Colossoma macropomum XP_036447151.1

-<-EMILIN-3-<--<-AAR2-<-->-translocating chain-associated membrane protein 1-L 1->-->-nuclear receptor coactivator 3-L->-->-bifunctional apoptosis regulator-L->-->-ARL6IP5B->-->-SRSF6->-->-L3MBTL1->-->-SGK2->-->-MYBL2A->-->-AIG-L->-->-PTPDC1B->--<-FBXW12-<-->-WNT7A->--<-FBLN2-L-<-->-NUP210->-->-IQSEC1A->-->-GP9->--<-CNBPA-<--<-RAF1A-<--<-PPARG-L-<--<-SYN2A-<-

Gadiformes

Gadus morhua XP_030218440.1

->-MBD4->--<-CRELD1-<--<-interleukin-17 receptor E-L-<--<-BRPF1-<--<-WNK2-L-<--<-FGD3-<--<-BICD2-L-<-->-AIG-L->-->-PTPDC1B-L->--<-FBXW12-<-->-WNT7A->--<-FBLN2-<-->-NUP210->-->-IQSEC1A->-->-GP9-L->--<-haloacid dehalogenase-L hydrolase domain-containing 5-<--<-CNBP-L-<-->-RAF1->--<-SYN2A-L-<-

Gonorhynchiformes

Chanos chanos XP_030625604.1

-<-sodium- and chloride-dependent creatine transporter 1-L-<-->-secreted frizzled-related protein 5-L->--<-transmembrane prolyl 4-hydroxylase-L-<--<-deoxyribonuclease gamma-L-<--<-nuclear receptor coactivator 3-L-<--<-translocating chain-associated membrane protein 1-L 1-<-->-protein kinase C-binding protein 1-L->-->-SRSF6->-->-L3MBTL1->-->-SGK2->-->-AIG-L->-->-PTPDC1B-L->--<-FBXW12-<-->-WNT7A-L->--<-FBLN2-L-<-->-NUP210->-->-IQSEC1A-L->-->-GP9-L->--<-CNBP-L-<-->-RAF1-L->--<-PPARG-L-<--<-SYN2A-L-<-

Anguilliformes

Anguilla anguilla(European eel) XP_035236387.1

-<-FRS3-<--<-GUCA1B-<--<-transmembrane and coiled-coil domains protein 2-L-<-->-ubiquinol-cytochrome-c reductase complex assembly factor 2->--<-TSPO-<--<-bcl-2 homologous antagonist/killer-L-<-->-TOMM6->--<-c11h1orf74-<-->-FAM50A->-->-AIG-L->-->-ALAS2->--<-IMP4-<-->-receptor tyrosine-protein kinase erbB-3-L->-->-proliferation-associated protein 2G4-L->--<-carcinoembryonic antigen-related cell adhesion molecule 1-L-<--<-immunoglobulin superfamily member 3-L-<-->-zinc finger CCCH domain-containing protein 10-L->-->-ESYT1A->-->-FMNL3->--<-low-density lipoprotein receptor-related protein 1-L-<-->-binding motif, single-stranded-interacting protein 2-L->-

Elopiformes

Megalops cyprinoides XP_036389293.1

-<-MYBL2A-<--<-L3MBTL1-<--<-SRSF6A-<-->-SNX21->-->-WFDC2->--<-PIGT-<-->-PHACTR3->--<-SYCP2-<--<-TTLL9-<-->-AIG-L->--<-ALAS2-<--<-SARS1-<-->-PPIL1->--<-sodium- and chloride-dependent GABA transporter 2-L-<--<-sodium- and chloride-dependent GABA transporter 2-L-<-->-IMP4->-->-PROK1->-->-pepsin A-L->-->-pepsin A-L->-->-pepsin A-L->-

Holocentriforme

Myripristis murdjan XP_029912547.1

-<-CRELD1-<--<-BRPF1-<--<-WNK2-L-<-->-NINJ1-L->--<-CARD19-L-<--<-FGD3-L-<--<-BICD2-L-<-->-AIG-L->-->-PTPDC1B-L->--<-FBXW12-<-->-WNT7AB-L->--<-FBLN2-L-<-->-NUP210->-->-IQSEC1-L->-->-GP9-L->--<-haloacid dehalogenase-L hydrolase domain-containing 5-<--<-CNBP-L-<-->-RAF1A->--<-SYN2A-L-<-

Gymnotiformes

Electrophorus electricus XP_026857542.2

->-SMPD2A->-->-ARL6IP5B->-->-SRSF6A->-->-L3MBTL1->-->-SGK2A->-->-MYBL2A->-->-AIG-L->--<-FBXW12-<-->-WNT7A->--<-fibulin-2-L-<-->-NUP210->-->-IQSEC1-L->-->-GP9->--<-CNBP-<-->-RAF1A->--<-SYN2A-<-

Osteoglossiformes

Paramormyrops kingsleyae XP_023660738.1

-<-L3MBTL1-<--<-SRSF6-L-<-->-SNX21->-->-WFDC2-L->--<-PIGT-<-->-PHACTR3-L->--<-SYCP2-L-<-->-FAM217B-L->--<-TTLL9-<--<-EDN3-L-<-->-AIG-L->-->-PHF2-L->--<-homeobox protein BarH-L 1-<-->-FBXW12->-->-WNT7AB-L->-->-NUP210->-->-IQSEC1-L->-->-GP9-L->-->-GP9-L->--<-haloacid dehalogenase-L hydrolase domain-containing 5-<--<-CNBP-L-<-->-RAF1A-L->--<-SYN2A-L-<-

Scleropages formosus XP_018593069.1

->-CD209 antigen-L protein C->-->-C-type lectin domain family 4 member M-L->--<-lipopolysaccharide-induced tumor necrosis factor-alpha factor homolog-<--<-PIGT-<-->-PHACTR3-L->--<-SYCP2-<-->-FAM217B->--<-TTLL9-<--<-TTLL9-<--<-TTLL9-<-->-AIG-L->-->-PHF2-L->--<-homeobox protein BarH-L 1-<-->-PTPDC1B-L->-->-FBXW12->-->-WNT7AB-L->-->-NUP210->-->-IQSEC1-L->-->-GP9-L->-->-GP9-L->--<-haloacid dehalogenase-L hydrolase domain-containing 5-<--<-CNBP-L-<-->-RAF1A-L->--<-SYN2A-L-<-

Clupeiformes

Denticeps clupeoides XP_028848591.1

->-protein phosphatase 1 regulatory subunit 3D-L->-->-synaptonemal complex protein 2-L->--<-PHACTR3-L-<-->-PIGT->--<-WFDC2-L-<--<-SNX21-L-<-->-serine/arginine-rich splicing factor 6-L->-->-L3MBTL1->-->-SGK2->-->-AIG-L->-->-PTPDC1B-L->--<-FBXW12-<-->-WNT7AB-L->--<-HSD17B10-<--<-FAM120C-<-->-isocitrate dehydrogenase [NAD] subunit gamma, mitochondrial-L->-->-AVPR2AB-L->--<-SEPHS2-L-<-->-RIIAD1->--<-SAT2B-L-<--<-SSR4-L-<-

Esociformes

Esox lucius XP_010893603.1

-<-CRELD1-<--<-interleukin-17 receptor C-<--<-interleukin-17 receptor C-<--<-BRPF1-<--<-WNK2-<-->-NINJ1->--<-caspase recruitment domain-containing protein 19-<--<-sushi domain-containing protein 3-<--<-FGD-<--<-BICD2-<-->-AIG-L->-->-PTPDC1B->--<-FBXW12-<-->-WNT7AB->--<-fibulin-2-<-->-NUP210->-->-IQSEC1-L->--<-haloacid dehalogenase-L hydrolase domain-containing 5-<--<-CNBP-<-->-RAF1A->--<-PPARG-<--<-SYN2A-<-

Acipenseriformes

Acipenser ruthenus XP_033897210.2

-<-synaptonemal complex protein 2-<-->-phosphatase and actin regulator 3-L->--<-synaptonemal complex protein 2-L-<--<-protein phosphatase 1 regulatory subunit 3D-L-<-->-PDRG1-L->-->-PDRG1-L->--<-probable tubulin polyglutamylase TTLL9-<--<-EDN-3-L-<-->-AIG-L->-->-cleavage stimulation factor subunit 1->-->-cas scaffolding protein family member 4-L->--<-RNA-binding protein 38-L-<--<-export factor isoform X2-<--<-meiotic recombination protein SPO11-L-<-->- bone morphogenetic protein 7-L->-->-IRF10-L->-->-dolichyl-diphosphooligosaccharide--protein glycosyltransferase subunit 2-L->--<-GHRH-<-

Acipenser ruthenus XP_033911083.2

-<-synaptonemal complex protein 2-L-<-->-phosphatase and actin regulator 3-L->--<-synaptonemal complex protein 2-<--<-synaptonemal complex protein 2-L-<--<-protein phosphatase 1 regulatory subunit 3D-<-->-PDRG1-L->--<-probable tubulin polyglutamylase TTLL9-<--<-EDN-3-L-<--<-ZNF813-<-->-AIG-L->-->-cleavage stimulation factor subunit 1->-->-cas scaffolding protein family member 4-L->--<-RNA-binding protein 38-L-<--<-export factor isoform X2-<--<-meiotic recombination protein SPO11-L-<-->- bone morphogenetic protein 7-L->-->-IRF10-L->--<-zinc finger CCCH domain-containing protein 18-L-<-->-dolichyl-diphosphooligosaccharide--protein glycosyltransferase subunit 2-L->-->-mitochondrial tRNA methylthiotransferase CDK5RAP1-L->-

Salmoniformes

Oncorhynchus keta XP_035597206.1

->-MBD4->--<-CRELD1-L-<--<-LOC115541628-<--<-interleukin-17 receptor C-<--<-BRPF1-L-<--<-WNK2-L-<--<-LOC118361414-<--<-FGD3-L-<--<-BICD2-L-<-->-AIG-L->-->-PTPDC1B->--<-FBXW12-<-->-WNT7A-L->--<-FBLN2-L-<-->-NUP210->-->-IQSEC1A-L->-->-GP9->--<-haloacid dehalogenase-L hydrolase domain-containing 5-<--<-CNBPA-<--<-RAF1A-<--<-SYN2A-<-

Oncorhynchus keta XP_035594065.1

-<-IFT122-<--<-CRELD1-<--<-interleukin-17 receptor C-L-<--<-interleukin-17 receptor C-L-<--<-BRPF1-L-<--<-WNK2-L-<-->-NINJ1-L->--<-CARD19-L-<--<-FGD3-L-<--<-BICD2-L-<-->-AIG-L->-->-PTBDC1-L->-->-WNT7AB->--<-FBLN2-L-<-->-NUP210-L->-->-NUP210-L->-->-IQSEC1-L->--<-haloacid dehalogenase-L hydrolase domain-containing 5-<--<-CNBP-L-<-->-RAF1A-L->--<-PPARG-L-<--<-SYN2A-L-<-

Oncorhynchus kisutch XP_020361527.1

->-MBD4->--<-CRELD1-<--<-interleukin-17 receptor C-L-<--<-BRPF1-L-<--<-WNK2-<--<-zinc finger protein 37-<-->-BRD3-L->-->-SNAP29->--<-CIZ1B-<--<-

LOC109907755-<--<-FGD3-<--<-BICD2-<-->-AIG-L->-->-PTPDC1B->--<-FBXW12-<-->-WNT7A->--<-FBLN2-<-->-NUP210->-->-IQSEC1A->-->-GP9->--<-haloacid dehalogenase-L hydrolase domain-containing 5-<--<-CNBPA-<--<-RAF1A-<--<-PPARG-<--<-SYN2A-<-

Oncorhynchus kisutch XP_020314827.2

-<-IFT122-<--<-CRELD1-<--<-interleukin-17 receptor C-<--<-interleukin-17 receptor C-<--<-BRPF1-L-<--<-WNK2-<-->-NINJ1-L->--<-CARD19-<--<-FGD3-L-<--<-BICD2-L-<-->-AIG-L->-->-protein tyrosine phosphatase domain-containing protein 1-L->-->-WNT7AB->--<-FBLN2-L-<-->-IQSEC1-L->--<-haloacid dehalogenase-L hydrolase domain-containing 5-<--<-CNBP-<-->-RAF1A->--<-PPARG-L-<--<-SYN2A-<-

Oncorhynchus nerka XP_029519793.1

->-MBD4->-->-LOC115131856->--<-CRELD1-<--<-interleukin-17 receptor C-L-<--<-BRPF1-L-<--<-zinc finger protein 37-L-<-->-BRD3-L->-->-SNAP29->--<-CIZ1B-L-<--<-CARD19-L-<--<-LOC115131866-<--<-FGD3-L-<--<-BICD2-L-<-->-AIG-L->-->-PTPDC1B-L->--<-FBXW12-<-->-WNT7A-L->--<-FBLN2-L-<-->-NUP210->-->-IQSEC1A-L->-->-GP9-L->--<-haloacid dehalogenase-L hydrolase domain-containing 5-<--<-CNBPA-L-<--<-RAF1A-L-<--<-PPARG-L-<--<-SYN2A-L-<-

Oncorhynchus nerka XP_029477517.1

-<-IFT122-<-->-methyl-CpG-binding domain protein 4-L->--<-CRELD1-<--<-BRPF1-L-<--<-WNK3-L-<--<-WNK2-L-<-->-NINJ1-L->--<-CARD19-<--<-FGD3-L-<--<-BICD2-<-->-AIG-L->-->-PTBDC1->-->-WNT7AB->--<-FBLN2-L-<-->-IQSEC1-L->--<-haloacid dehalogenase-L hydrolase domain-containing 5-<--<-CNBP-L-<-->-RAF1A->--<-PPARG-L-<--<-SYN2A-L-<-

Oncorhynchus tshawytscha XP_024230885.1

-<-CRELD1-<--<-LOC115131859-<--<-BRPF1-L-<--<-WNK2-L-<--<-neurotrophin receptor-interacting factor 2-L-<-->-BRD3-L->-->-SNAP29->-->-NYNRIN-L->--<-CIZ1B-L-<--<-pheromone-regulated protein PRM7-L-<--<-LOC112215751-<--<-FGD3-<--<-BICD2-L-<-->-AIG-L->-->-PTPDC1B-L->--<-FBXW12-<-->-WNT7A->--<-FBLN2-L-<-->-NUP210->-->-IQSEC1A-L->-->-GP9-L->--<-haloacid dehalogenase-L hydrolase domain-containing 5-<--<-CNBPA-L-<--<-RAF1A-L-<--<-PPARG-L-<--<-SYN2A-L-<-

Oncorhynchus tshawytscha XP_024240695.1

-<-IFT122-<-->-methyl-CpG-binding domain protein 4-L->--<-CRELD1-L-<--<-BRPF1-L-<--<-WNK3-L-<--<-WNK2-L-<-->-NINJ1-L->--<-CARD19-L-<--<-FGD3-L-<--<-BICD2-L-<-->-AIG-L->-->-PTBDC1-L->-->-WNT7AB-L->--<-FBLN2-L-<-->-IQSEC1-L->--<-haloacid dehalogenase-L hydrolase domain-containing 5-<--<-CNBP-L-<-->-RAF1A-L->--<-PPARG-L-<--<-SYN2A-L-<-

Salmo trutta XP_029575706.1

-<-transcription initiation factor TFIID subunit 10-L-<-->-ZGC:154075->--<-coiled-coil domain-containing protein 114-L-<-->-ERBB receptor feedback inhibitor 1-L->-->-PAX7B-L->-->-KCNAB2-L->--<-LOC115166124-<--<-FGD3-L-<--<-BICD2-L-<-->-AIG-L->-->-PTPDC1B-L->--<-FBXW12-<-->-WNT7A-L->--<-FBLN2-L-<-->-NUP210-L->-->-IQSEC1A-L->-->-GP9-L->--<-haloacid dehalogenase-L hydrolase domain-containing 5-<--<-CNBPA-L-<--<-RAF1A-L-<--<-PPARG-L-<--<-SYN2A-L-<-

Salmo trutta XP_029579514.1

-<-IFT122-<-->-methyl-CpG-binding domain protein 4-L->--<-CRELD1-L-<--<-BRPF1-L-<--<-WNK2-L-<-->-NINJ1-L->--<-CARD19-L-<--<-sushi domain-containing protein 3-L-<--<-histidine-rich glycoprotein-L-<--<-histidine-rich glycoprotein-L-<--<-FGD3-L-<--<-BICD2-L-<-->-AIG-L->-->-PTBDC1-L->-->-WNT7AB->--<-FBLN2-L-<-->-IQSEC1-L->--<-haloacid dehalogenase-L hydrolase domain-containing 5-<--<-CNBP-L-<-->-RAF1A-L->--<-PPARG-L-<--<-SYN2A-L-<-

Salmo salar XP_014000908.1

->-methyl-CpG-binding domain protein 4-L->--<-CRELD1-L-<--<-interleukin-17 receptor E-L-<--<-BRPF1-L-<--<-C7orf26-<--<-bcl10-interacting CARD protein-L-<--<-LOC106571891-<--<-FGD3-L-<--<-BICD2-L-<-->-AIG-L->-->-PTPDC1B-L->--<-FBXW12-<-->-WNT7A-L->--<-FBLN2-L-<-->-NUP210-L->-->-IQSEC1A-L->-->-GP9-L->--<-cat eye syndrome critical region protein 5-L-<--<-CNBP-<--<-RAF1A-L-<--<-PPARG-<--<-SYN2A-<-

Salvelinus alpinus XP_023846417.1

-<-DOCK3-<--<-DDB1- and CUL4-associated factor 1-<-->-60S ribosomal protein L32->-->-glutamate receptor-interacting protein 2->-->-glutamate receptor-interacting protein 2->--<-sodium- and chloride-dependent taurine transporter-L-<-->-transmembrane and coiled-coil domains protein 1-L->-->-plexin-D1-L->-->-plexin-D1->--<-rhodopsin-L-<--<-sushi domain-containing protein 3-<--<-FGD3-<--<-BICD2-<-->-AIG-L->-->-PTPDC1B-L->--<-FBXW12-<-->-WNT7A->--<-FBLN2-L-<--<-FBLN2-<-->-NUP210->-->-IQSEC1A->-->-GP9-L->--<-haloacid dehalogenase-L hydrolase domain-containing 5-<--<-CNBPA-<--<-RAF1A-<--<-PPARG-<-->-VGLL4A->-

Salvelinus alpinus XP_023862004.1

-<-CRELD1-L domain protein 1-<--<-interleukin-17 receptor C-L-<--<-BRPF1-<--<-WNK2-L-<-->-NINJ1-L->--<-caspase recruitment domain-containing protein 19-<--<-FYVE, RhoGEF and PH domain-containing protein 3-L-<--<-BICD2-<-->-AIG-L->-->-PTPDC1B1-L->-->-protein Wnt-7a-L->--<-fibulin-2-L-<-->-NUP210-L->-->-IQSEC1-L->-->-GP9-L->--<-haloacid dehalogenase-L hydrolase domain-containing 5-<--<-CNBP-<-->-RAF1A->--<-synapsin-2-<-

Oncorhynchus mykiss(rainbow trout) XP_036803289.1

-<-IFT122-<--<-CRELD1-<--<-interleukin-17 receptor C-<--<-interleukin-17 receptor C-L-<--<-BRPF1-L-<--<-WNK2-L-<-->-NINJ1-L->--<-CARD19-<--<-FGD3-L-<--<-BICD2-<-->-AIG-L->-->-PTBDC1-L->-->-WNT7AB->--<-FBLN2-<-->-NUP210->-->-IQSEC1-L->--<-haloacid dehalogenase-L hydrolase domain-containing 5-<--<-CNBP-<-->-RAF1A->--<-PPARG-L-<--<-SYN2A-<-

Oncorhynchus mykiss(rainbow trout) XP_021471213.1

-<-CRELD1-<--<-BRPF1-<--<-WNK2-<-->-cip1-interacting zinc finger protein->--<-SNAP29-<--<-BRD3-<-->-AZF1->--<-LOC118936440-<--<-LOC110531980-<--<-FGD3-L-<--<-BICD2-<-->-AIG-L->--->-PTPDC1B->--<-FBXW12-<-->-WNT7A->--<-FBLN2-L-<-->-NUP210->-->-IQSEC1->-->-GP9->--<-haloacid dehalogenase-L hydrolase domain-containing 5-<--<-CNBP-<--<-RAF1A-<--<-PPARG-<--<-SYN2A-<-->-VGLL4A->-

Cyprinina

Carassius auratus(goldfish) XP_026097278.1

->-small integral membrane protein 29-L->--<-high mobility group protein HMG-I/HMG-Y-L-<--<-SLC9A8-<-->-B4GALT5-L->--<-EMILIN-3-L-<--<-AAR2-<-->-translocating chain-associated membrane protein 1-L 1->--<-SRSF6A-L-<--<-ARL6IP5B-L-<-->-SGK2A-L->-->-AIG-L->-->-PTPDC1B-L->--<-FBXW12-<-->-WNT7A-L->-->-slc34a2b-L->-->-UTRN-L->--<-FBXO30B-L-<--<- fibronectin type III domain-containing protein 7-L-<--<- fibronectin type III domain-containing protein 7-L-<--<-fibronectin type III domain-containing protein 7-L-<--<-fibronectin type III domain-containing protein 7-L-<-->-STXBP-L3->-->-RABGGTA->-->-NACHT, LRR and PYD domains-containing protein 12-L->-->-SMC1AL->-->-6-phosphofructo-2-kinase/fructose-2,6-bisphosphatase-L->--<-NDNL2-<-

Cyprinus carpio(common carp) XP_018960320.1

-<-mitochondrial fission regulator 1-L-<-->-ARMC1-L->-->-SHROOM2-L->-->-claudin-34-L->--<-CLDN24-<--<-L3MBTL1-<--<-SRSF6A-L-<--<-ARL6IP5B-L-<-->-SGK2A-L->-->-AIG-L->--<-FBXW12-L-<--<-BHLHE22-L-<--<-YTHDF3-L-<-->-PDHA1->-->-RPS6KA3-L->-->-RPS6KA3B->-->-ukaryotic translation initiation factor 1A, X-chromosomal-L->-->-CXorf23-L->--<-lipase member H-L-<-->-lysozyme C-L->-

Sinocyclocheilus anshuiensis XP_016336758.1

-<-AAR2-<-->-translocating chain-associated membrane protein 1-L 1->-->-ARL6IP5B-L->-->-SRSF6A-L->-->-LOC107684905->-->-L3MBTL1->-->-SGK2A-L->-->-AIG-L->-->-PTPDC1B-L->--<-FBXW12-<-->-WNT7A-L->--<-craniofacial development protein 2-L-<-->-NUP210-L->-->-IQSEC1-L->-->-IQSEC1-L->--<-CNBP-L-<-->-RAF1A-L->--<-SYN2A-L-<-

Sinocyclocheilus grahami XP_016094196.1

-<-AAR2-<-->-translocating chain-associated membrane protein 1-L 1->-->-ARL6IP5B-L->-->-SRSF6A-L->-->-LOC107555797->-->-L3MBTL1->-->-SGK2A-L->-->-AIG-L->-->-PTPDC1B-L->--<-FBXW12-<-->-WNT7A-L->-->-NUP210-L->-->-IQSEC1-L->-->-IQSEC1-L->--<-CNBP-L-<-->-RAF1A-L->--<-SYN2A-L-<-

Sinocyclocheilus rhinocerous XP_016374857.1

->-SGK2A-L->-->-AIG-L->-->-PTPDC1B-L->--<-FBXW12-<-->-WNT7A-L->-->-NUP210-L->-->-IQSEC1-L->-->-IQSEC1-L->--<-CNBP-L-<-->-RAF1A-L->--<-SYN2A-L-<-

---

Tetrapoda

Metatheria （Monodelphis domestica、Trichosurus vulpecula、Vombatus ursinus） XP_020837054.1, XP_012395107.1

-<-Phactr3-<--<-Edn3-<--<-ZNF831->-AIG-L->-->-PRELID3B->-->-ATP5F1E->--<-TUBB1--<-

Birds, reptiles, amphibians and Coelacanthimorpha

Palaeognathae （Apteryx mantelli mantelli， Apteryx rowi,Nothoprocta perdicaria, Struthio camelus australis, Tinamus guttatus,）

-<-Phactr3-<--<-Edn3-<--<-ZNF831->-AIG-L->-->-PRELID3B->-->-ATP5F1E->--<-TUBB1--<-
